# Autophagy formation, microtubule disorientation, and alteration of ATG8 and tubulin gene expression under simulated microgravity in *Arabidopsis thaliana*

**DOI:** 10.1038/s41526-024-00381-9

**Published:** 2024-03-18

**Authors:** Alla Yemets, Ruslana Shadrina, Rostyslav Blume, Svitlana Plokhovska, Yaroslav Blume

**Affiliations:** Institute of Food Biotechnology and Genomics, National Academy of Sciences of Ukraine, Baidy-Vyshnevetskoho St., 2a, Kyiv, 04123 Ukraine

**Keywords:** Cell biology, Plant sciences, Molecular biology

## Abstract

Autophagy plays an important role in plant growth and development, pathogen invasion and modulates plant response and adaptation to various abiotic stress stimuli. The biogenesis and trafficking of autophagosomes involve microtubules (MTs) as important actors in the autophagic process. However, initiation of autophagy in plants under microgravity has not been previously studied. Here we demonstrate how simulated microgravity induces autophagy development involving microtubular reorganization during period of autophagosome formation. It was shown that induction of autophagy with maximal autophagosome formation in root cells of *Arabidopsis thaliana* is observed after 6 days of clinostating, along with MT disorganization, which leads to visible changes in root morphology. Gradual decrease of autophagosome number was indicated on 9^th^ and 12^th^ days of the experiment as well as no significant re-orientation of MTs were identified. Respectively, analysis of α- and β-tubulins and *ATG8* gene expression was carried out. In particular, the most pronounced increase of expression on both 6^th^ and 9^th^ days in response to simulated microgravity was detected for non-paralogous *AtATG8b, AtATG8f, AtATG8i*, and *AtTUA2, AtTUA3* genes, as well as for the pair of β-tubulin duplicates, namely *AtTUB2* and *AtTUB3*. Overall, the main autophagic response was observed after 6 and 9 days of exposure to simulated microgravity, followed by adaptive response after 12 days. These findings provide a key basis for further studies of cellular mechanisms of autophagy and involvement of cytoskeletal structures in autophagy biogenesis under microgravity, which would enable development of new approaches, aimed on enhancing plant adaptation to microgravity.

## Introduction

One of the fundamental problems in space biology is the creation of autotrophic link technologies in the life support systems of astronauts on long-term space flights. For this purpose, it is also necessary to use plants that complete their entire ontogenesis in one cycle under the conditions of space^1^. So far, seeds of several genera including *Arabidopsis*, *Brassica* and *Triticum* have been collected in spaceflight^1,2^. *Arabidopsis* seeds formed during space flight were no different from ground control, but molecular analysis collected from *Arabidopsis* seedlings exposed to microgravity showed significant effects on post-transcriptional regulation, plastid gene transcription, redox homeostasis, cell wall synthesis, and MT dynamics^3,4^. Thus, the study of cellular mechanisms that are involved in responses to microgravity stress and possible ways of their regulation pose a challenge of significant importance in the field of space biology.

As sessile organisms, plants have developed numerous response mechanisms to adapt to diverse stress growing conditions, such as soil salinity, drought, and extreme temperatures. One of the approaches is to utilize the damaged protein and organelles which can be produced in plant cells^5^. Autophagy is a fundamental catabolic process in cells, responsible for the degradation and recycling of long-lived macromolecules and organelles^6–8^. Essential autophagy occurs without stress conditions and can be quickly induced by nutrient deprivation and various stress factors to ensure the removal of extinct organelles and damaged proteins from the cytosol. Basal autophagy supports cellular homeostasis, leaf senescence, root meristem maintenance, pollen germination, and seed development^9^. During autophagy, impaired organelles and protein aggregates are delivered to double-membrane cisternae called autophagosomes for processing into a vacuole^7^. The autophagosome contains hydrolases and proteases which metabolize proteins and fats into amino acids and fatty acids, respectively. There are a few general types of autophagy. Microautophagy occurs by invagination of the tonoplast to capture cytoplasmic aggregates collected on the surface of the vacuole to form autophagic bodies inside the vacuole^10^. Macroautophagy (referred to herein as autophagy) involves the formation of autophagosomes - double-membrane vesicles that envelop cytoplasmic cargo^11^. Megaautophagy is triggered either by programmed cell death or by pathogen invasion, whereupon the vacuole releases its contents into the cytoplasm, allowing hydrolases to degrade all organelles and the cell wall leading to cell death. To date, only microautophagy and macroautophagy processes have been reported in plants^12^.

Autophagy is a highly regulated dynamic process mediated by AuTophaGy (ATG) proteins that control the formation of autophagosomes^13^. Almost half of the more than 40 identified autophagic proteins is part of the core autophagic machinery conserved across all kingdoms, including such plant as *Arabidopsis thaliana*^14^. Research over the past decade has shown that autophagy is highly selective and occurs in three main steps: recognition of autophagic cargo, it’s recruitment to the phagophore, and then removal of the captured cargo by fusion of the autophagosome with the vacuole^15,16^. Most of the ATG proteins in plants can be divided into 5 subcomplexes: the ATG1 complex, phosphoinositide 3-kinase (PI3K) complex, ATG9 and two ubiquitin-like conjugation systems (ATG5-ATG12 and ATG8)^17^.

Previously autophagy was mainly considered a non-selective type of bulk processing. Recent evidence indicates that selective autophagy degrades specific targets, proteins and unwanted organelles^7^. The ATG8 protein is essential for both selective and bulk autophagy^18^. In selective autophagy, ATG8 plays a role in component binding, serving as a docking platform to adapters and receptors that harvest the cargo for further degradation in ubiquitin-like manner. Mature ATG8, which is processed by the ATG4 protease, binds to the membrane lipid phosphatidylethanolamine (PE) in *A*. *thaliana*. As a result, the ATG8-PE complex assists autophagosome biogenesis via membrane reshaping and tethering^19,20^. ATG8 can be found on both inner and outer membranes of autophagosome, which makes ATG8 a convenient marker for monitoring autophagy in the cells^21^. Fusions of green fluorescent protein (GFP) and ATG8 protein are commonly used to track autophagy fluxes^22^. There are nine *ATG8* (*ATG8a–i*) genes in *Arabidopsis*. Various ATG8 proteins are predicted to have spare functions, whereas single atg8 mutations display no visible phenotype nor affect autophagic activity^13^.

Autophagosomes are mobile structures that require MTs to move. Nocodazole, an efficient inhibitor of MT polymerization in animal cells, induces autophagosome accumulation, indicating inhibition of autophagosome fusion with late endosomes or lysosomes^23^. Similarly, conflicting data have been obtained on the effect of vinblastine, another inhibitor of MT polymerization, on autophagosome fusion, and an increase in the number of autophagosomes has been shown after treatment of cells with vinblastine^24^. MT disruption leads to a significant reduction in the number of mature autophagosomes but does not affect their life expectancy or fusion with lysosomes. MTs are suggested to deliver only mature autophagosomes for degradation and thus create a spatial barrier between phagophores and lysosomes^25^. There is mounting evidence for the role of MTs in autophagy in yeast and mammalian cells, but data in plants are scarce. In yeast and animal cells, MTs ensure maturation and movement of autophagosomes through their dynamic state changes and post-translational modifications of tubulin^26,27^. MTs form a complex interconnected network that often functions as pathways for intracellular metabolism controlled by specific motor proteins that are part of the kinesin or dynein protein families^28^.

At present, the functions of plant MTs and tubulin as their main constituent in autophagy development have not been sufficiently studied. In *A. thaliana*, the tubulin genes form a large family consisting of six α-tubulins (*TUAs*) and nine β-tubulins (*TUBs*)^29–31^. It was demonstrated earlier that the modulation of core proteins in the autophagy machinery by posttranslational modifications is widely involved in the initiation and progression of autophagy^32^. In particular, we obtained original evidence about the role of α-tubulin acetylation in the development of autophagy upon abiotic stimuli in *A. thaliana*. Increased acetylation of α-tubulin upon sucrose starvation, UV-B irradiation, osmotic, and salt stress indicate functional involvement of MTs during autophagy in plants^33^. The interaction between MTs kinesins and ATG8 during initial stages of autophagy development is important because MTs and their motors have been shown to regulate two major complexes involved in the initiation of autophagy in animal cell^6^: mTORC1 and class III PI 3-kinase complex. When nutrients are not available, the cytoplasmic pH rises, and kinesins KIF2A and KIF1B are released from lysosomes promoting their centripetal movement and mTORC1 inactivation^34^.

Microgravity represents a stress factor affecting plant morphology and growth^35^. Numerous space biology experiments have revealed abnormalities in the growth and reproductive development of *Arabidopsis* under microgravity conditions^2,36^. Some available data demonstrate the influence of microgravity on mesophyll cell formation in *Arabidopsis* and pea seedlings^36,37^. Even it is well known that numerous stimuli can induce autophagy in plants, but the possibility of similar impact of microgravity as well as potential mode of involvement of MT machinery on the development of this process in plant cells has not been elucidated yet. In this study we demonstrated the involvement of autophagy in the response to simulated microgravity in *A. thaliana* root cells and described initial steps of autophagosome formation. To establish possible links between the development of autophagy and the involvement of MTs in simulated microgravity response, we analysed in detail the patterns of co-expression of all *AtATG8, AtTUA* and *AtTUB* genes during this process. Identification of key genes responsible for interaction of MTs with autophagosomes during the intial steps of autophagy induced by simulated microgravity may ultimately lead to a better understanding of plant adaptation and their cultivation in extreme conditions, such as outer space.

## Results

### Clinostating affects the root growth and morphology of *A. thaliana*

**Fig. 1:**
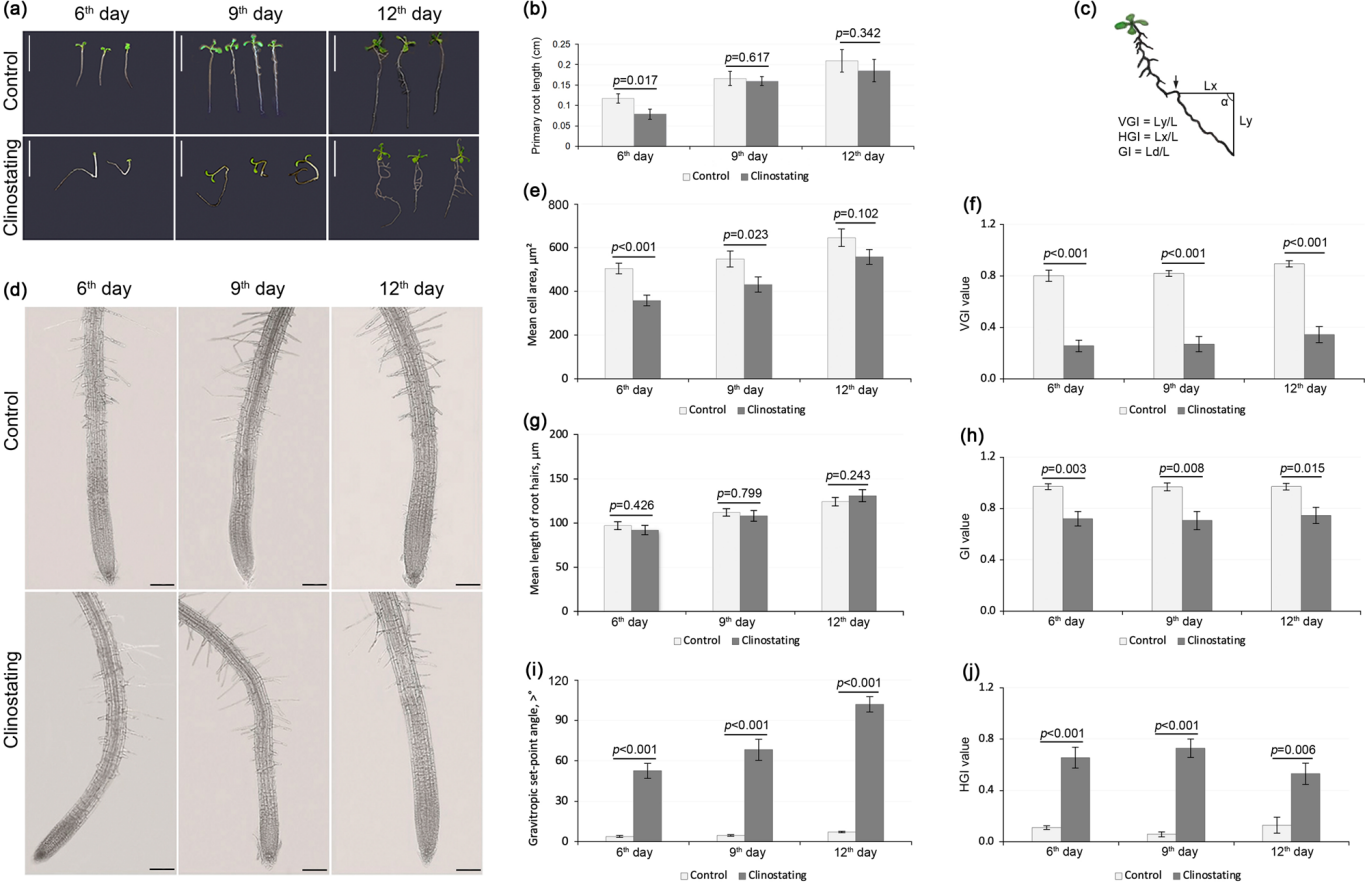
Effect of clinostating on *A. thaliana* primary root growth and morphology of 6-, 9-, and 12-days-old seedlings. **a** Photographs of seedlings in different periods of clinostating; bar – 1 cm; **b** Root growth, expressed as root length after 6-12 day of clinostating; two-way ANOVA, *p* > 0.05, n represents the number of plants, *n* = 30; **с** Schematic representation of measurement of root growth parameter; **e** Quantitative analysis of mean cell area of epidermal cells of the transition zone (*n* = 50); **f** VGI - Vertical Growth Index (*n* = 30)**;** **g** Growth of root hairs in seedlings under experimental conditions (*n* = 100); **d** Root appearance with diferent zones under clinostating; bar –100 µm; **h** GI - Gravitropic Index (*n* = 30)**;** **i** Root angle, expressed as the absolute value of integral average angular declination; **j** HGI - Horizontal Growth Index (*n* = 30). The level of the significance of the observed differences (*p*) for graphs (**b**) and **e**−**j** is indicated above the error bars (STD).

The influence of simulated microgravity on the growth and development of *A. thaliana* seedlings was investigated on the 6^th^, 9^th^, and 12^th^ days of clinostating, as these days are considered as key milestones of the autophagy development^38^. The study was conducted on 6-, 9-, and 12-day-old seedlings grown in horizontal clinostat (4 rpm), while control plants were germinated under normal conditions (without clinostating). The main morphological parameters of the seedlings were also evaluated, with special focusing on root development under experimental conditions (Fig. 1).

It was established that clinostating did not affect seed germination, as well as did not cause any significant deviations in shoot morphology of seedlings. A delay in the development of shoots was observed only on the 6^th^ and 9^th^ days of clinostating; on the 12^th^ day, the shoots were practically morphologically indistinguishable from the control plants. The 12-days-old seedlings had a regular leaf rosette that consisted of 4–6 oval green leaves. At the same time, the seedlings grown under experimental conditions, unlike the control ones, showed disoriented root growth, resulting from a constant change of the gravity vector (Fig. 1a). The average length of the primary roots (Fig. 1b) and root hairs (Fig. 1g) of the clinostated *A. thaliana* seedlings did not differ significantly from the control ones at all studied time intervals. However, it was found that the number of root hairs in plants increased during clinostating, and this was especially noticeable with the duration of their cultivation. In particular, on the 12^th^ day, the number of root hairs increased by 15% compared to the control.

Lateral roots play a significant role in the formation of a branched root network and therefore determine the plant’s capabilities in searching and consuming resources and mechanical attachment to the substrate. As a result of clinostating, more intensive formation and growth of lateral roots was observed after 12 days. In contrast to the control, the most characteristic of them was either a curved shape, or they formed and grew perpendicular to the primary roots.

When analyzing the morphology and development of primary roots during clinostating, some differences were found in their growth zones. Zones such as elongation and transition zones were shorter than those of control plant roots (Fig. 1d). In particular, the transition zone of the roots after 6 days of clinostating was 1.8-fold shorter, and after 9^th^ and 12^th^ days, respectively, 2-fold and 2.1-fold shorter compared to the control. There were also slight differences in cell sizes in the transition zone of primary roots. For example, when conducting a quantitative analysis of the mean cell area of epidermal cells in the transition zone (Fig. 1e), it was found that the area of epidermal cells after 6 days of clinostating decreased by 29%, and after 9 days - by 21% compared with control epidermal cells. However, after 12 days the difference in the mean cell area of root epidermal cells between the clinostated and control groups was approximately 14% that is statistically insignificant.

Phenotypic changes in primary roots caused by clinostating show that primary root development deviates from a model which can be described as vertical downward growth with a gravitropic set-point angle (GSA) equal to 0. Quantified evaluation of growth angle (Fig. 1i) showed a significant increase of GSA in clinostated *A. thaliana* seedlings that testifies about deviating from the normal vertical orientation of roots in control. This deviation is time-dependent, reaching a peak on the 12^th^ day of clinostating, which indicates gravitropic response of the root.

We also analyzed in more detail the root morphology using three values described before by *Villacampa* et al.^39^, in particular, gravitropic index (GI), vertical growth index (VGI) and horizontal growth index (HGI) (Fig. 1c, f, h, j). The GI is the shortest distance from the base of the root to the root tip (Ld) divided by the root length (L). GI is also called the straightness parameter and the closer the value is to one, the straighter the root. When analyzing control and clinostated seedlings, we obtained values of 0.9 and 0.7, respectively (Fig. 1h).

Statistical analysis indicated that VGI is a sensitive morphometric parameter enabling the detection of weak gravitropic defects. VGI is defined as the ratio between the straight-line distance from the base of the root to the root tip (Ly) and the root length (L). VGI in control plants is 0.9 at all time intervals (Fig. 1f). This indicates that the root grows downward (GSA = 0°) and exhibits positive gravitropism, its VGI approaches +1. In clinostating plants, VGI = 0.3 showed that the mean deviation of the roots from the vertical was higher. This indicates that roots have a diagravitropic phenotype (GSA = 90° or 270°), and its VGI approaches 0. In the case when a root grows upward, displaying negative gravitropism (GSA = 180°), VGI will be equal to −1.

VGI characterizes the deviation of root morphology from the model gravitropic phenotype, while HGI describes predominant lateral growth directions. HGI is the result of dividing the distance in the horizontal line between the base of the root and the root tip (Lx) by the root length (L). We obtained the opposite values when analyzing the HGI, in particular, for the control plants the HGI = 0.1 (Fig. 1j). Since the HGI is almost equal to 0, this indicates that the root grows vertically and has positive gravitropism (GSA = 0°). The reverse pattern was observed for wedge-shaped clinostated seedlings, HGI = 0.7 which indicates the deviation of the root tip to the right or left from the vertical. If the HGI approaches 1, this indicates diagravitropic phenotypes with horizontal root growth patterns.

### Effect of clinostating on MT organization in *A. thaliana* root cells

**Fig. 2:**
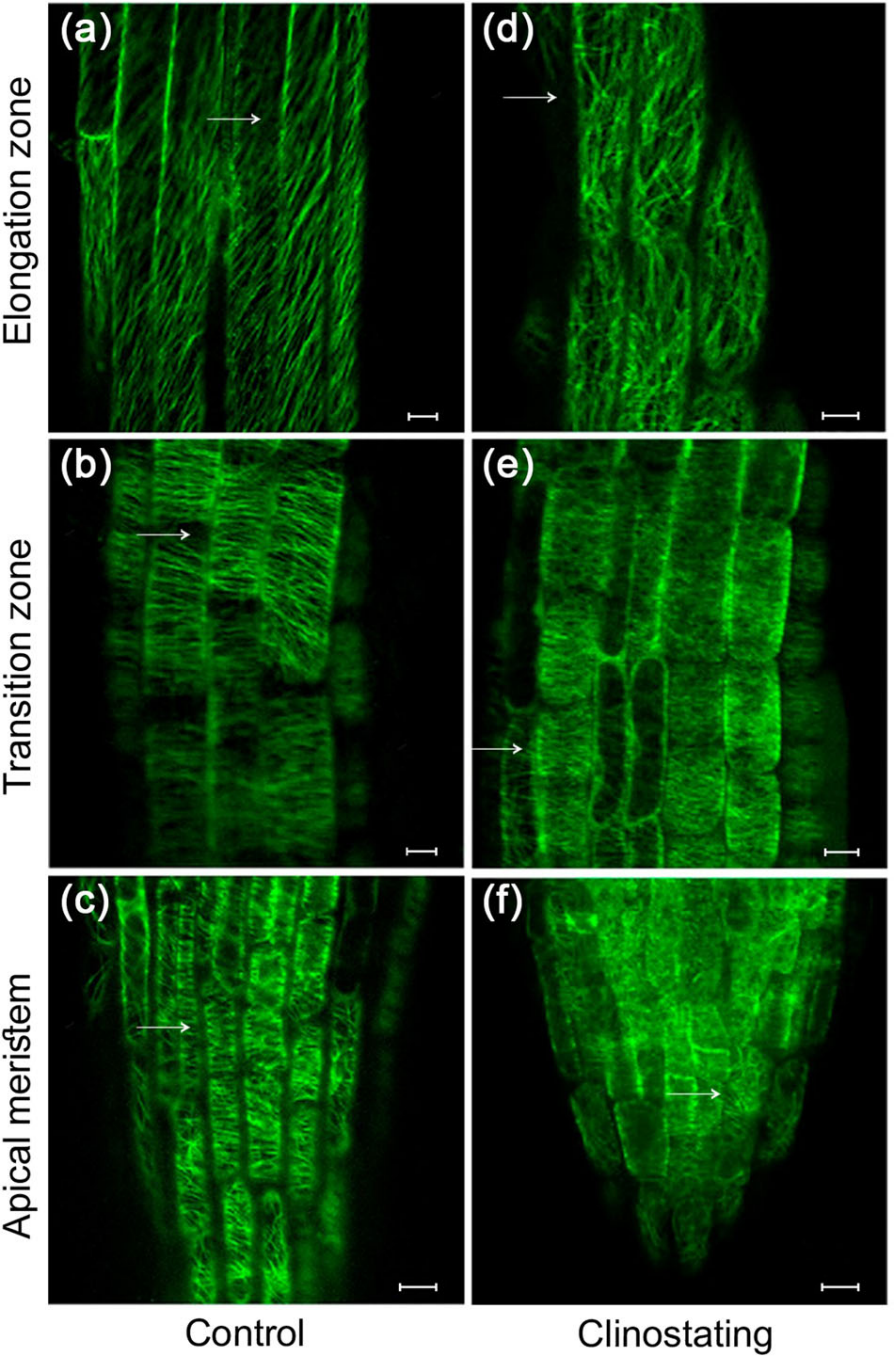
Effect of clinostating on MTs orientation in different root cells of *A. thaliana* GFP-MAP4 line on the 6^th^ day of growth under microgravity. **a**−**c** control, **d−f** clinostating. First row (**a**, **d**) – elongation zone, second row (**b**, **e**) – transition zone, third row (**c**, **f**) – root apex. Вar - 10 µm. Arrows indicate the examples of microtubule (re)orientation.

The negative effects of clinostating on cell division and elongation, leading to primary root growth deviations, may be related to the disturbances in mictubule (MT) organization. The expression of the chimeric gene *GFP-MAP4* in the *A. thaliana* line (GFP-MAP4) allowed us to study the tissue-specific organization of MTs in living root cells when this line was grown under altered gravity conditions (Fig. 2).

The changes in the typical orientation and organization of cortical MTs in different types of root cells were observed on the 6^th^ day of clinostaining. In control plants, MTs in epidermal cells of the meristem zone predominantly had transverse orientation (Fig. 2c). Additionaly, transversely oriented MTs, endoplasmic MTs, radially extending from the nucleus were also visualized in interphase meristem cells. In the dividing cells of the meristem, all types of mitotic MTs were found: the preprophase band, the mitotic spindle, and the phragmoplast. In the epidermal cells of the transition zone, cortical MTs of mainly transverse orientation were visualized (Fig. 2b); whereas in the zone of elongation, an oblique orientation of MTs was detected (Fig. 2a).

It was found that in 6-day-old plants grown under simulated microgravity, the organization of MTs had been significantly altered in the cells of some root growth zones. In particular, in the epidermal cells of the root apex and the meristem zone (Fig. 2f), the cortical MTs changed their orientation from transverse to a disordered one. Changes in the orientation of cortical MTs from transverse to chaotic were visualized in the epidermal cells of the transition zone (Fig. 2e), as well as in the elongation zone of *Arabidopsis* roots (Fig. 2d). It was also found that clinostating led to a reorientation of the MTs from typical oblique orientation to disordered one in the epidermal cells of the elongation zone (Fig. 2d). After 9^th^ and 12^th^ days of clinostating, stochastically occuring MT disorganization was rarely detected in the cells of all root growth zones. However, these changes in MT organization did not lead to dramatic disturbances in root growth and morphology, as we pointed out above.

Thus, the obtained data show that simulated microgravity cause disturbances in MT organization due to constant changes in plant exposition relative to the vector of gravity. All these suggest that MTs themselves can participate in gravity perception, and their structural reorganization is one of the elements of the gravity sensitivity of root cells, as it was also discussed earlier^40,41^.

### Microgravity-induced development of autophagy in *A. thaliana* root cells

**Fig. 3:**
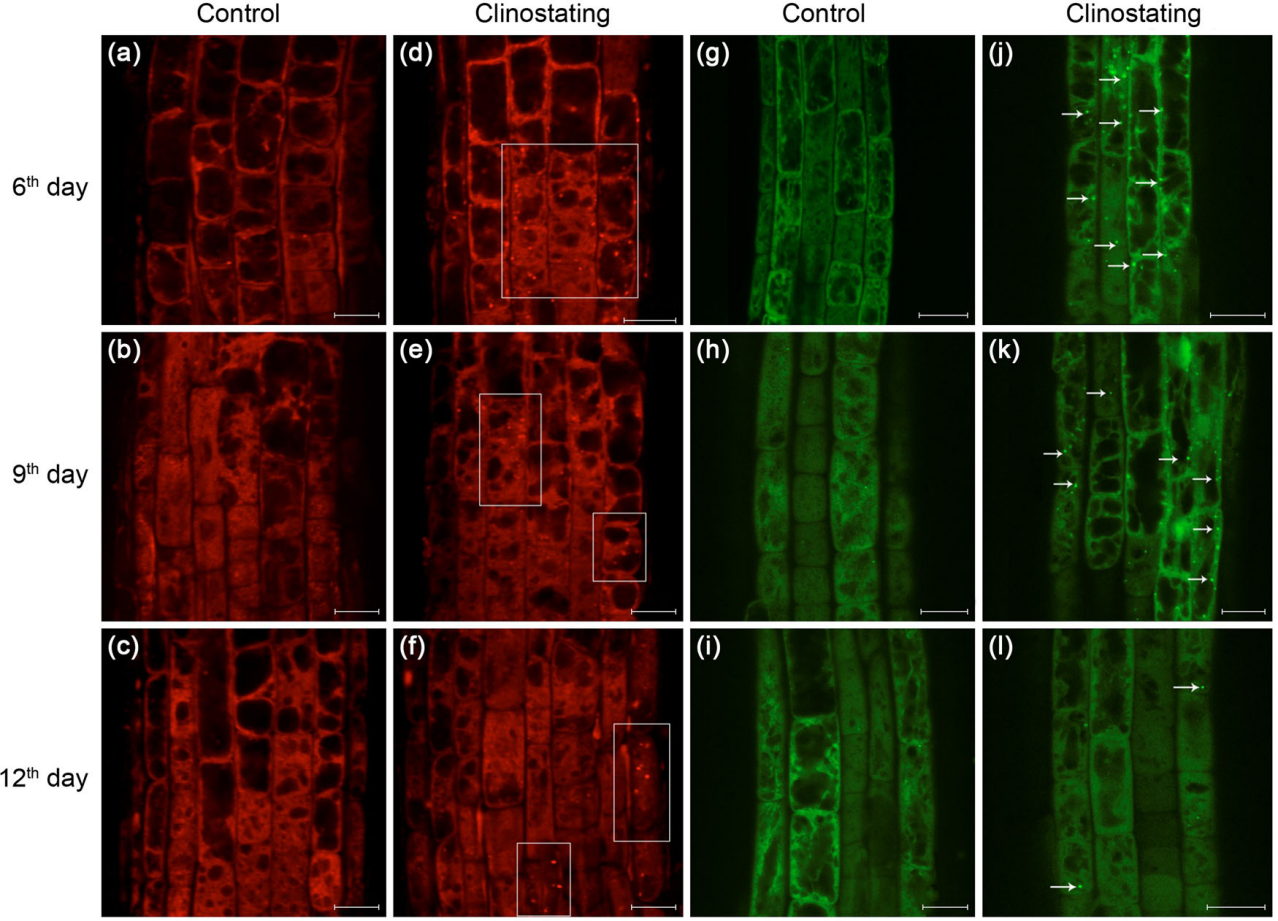
Induction of autophagy by microgravity-like clinostating conditions on 6^th^, 9^th^ and 12^th^ day of cultivation in root cells of *A. thaliana.* **a**−**f** roots treated with LysoTracker™ Red for visualization of autophagosomes (framed) in epidermal cells of the transition zone; **g**−**l** cells of the transition zone *A. thaliana* transgenic line with GFP-ATG8a-labeled autophagosomes (indicated by arrows). Вar - 20 µm.

In order to detect more precisely the development of autophagy in *A. thaliana* roots induced by simulated microgravity, a combination of methods was used. To perform that, 6-, 9-, and 12-days-old WT seedlings were treated with LysoTracker™ Red (LTR) dye, while plants of GFP-ATG8a line, grown under the same conditions, were used for parallel screening of clinostating-mediated autophagy induction (Fig. 3), since LTR may also stain different types of acidic organelles. It should be noted that individual autophagosomes were observed in root cells even in control plants, although their number increased with clinostating. As shown in (Fig. 3a, d), on the 6^th^ and 9^th^ days, the increase of autophagosome number was observed in the epidermal cells of the root transition zone, whereas few were found in similar root cells grown under control conditions. These data indicate that simulated microgravity can promote autophagy. On the 12^th^ day, only a small number of autophagosomes was observed in the epidermal cells of the root transition zone (Fig. 3f). Hence, obtained data could indicate the adaptation of plants to stressful conditions, caused by gravitational changes.

To verify the data obtained with LTR staining we also used the transgenic line of *A. thaliana* (GFP-ATG8a) to study dynamic changes of autophagy development under clinostating conditions. We obtained comparable results using this transgenic line. In particular, in plants, grown under simulated microgravity, the number of autophagosomes (GFP-ATG8a-labeled structures) increased in the epidermal cells of the root transition zone on the 6^th^ and 9^th^ days in comparison to control (Fig. 3j, k). The diameter of the autophagosomes was approximately 1 µm, and the presence of these puncta was highlighted by white arrows on supported images. A decrease in the number of autophagosomes in epidermal cells of transition zone of roots of GFP-ATG8a line has been detected on the 12^th^ day of clinostating (Fig. 3l). These results are consistent the described above findings, obtained using the LTR staining.

**Fig. 4:**
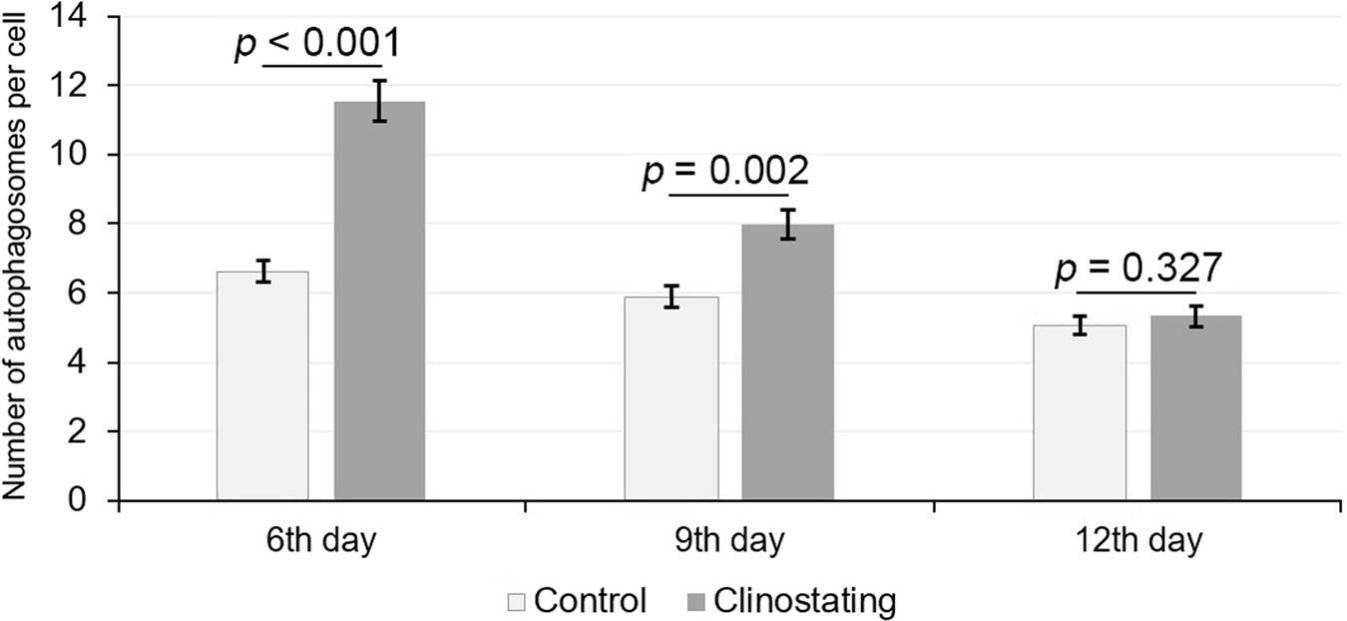
Number of autophagosomes in plants of the transgenic GFP-ATG8a *A. thaliana* line after 6, 9, and 12 days of growth under control and microgravity conditions. The level of the significance of the observed differences (*p*) in autophagosome number per cell (*n* = 30) is indicated above the error bars (STD).

Both GFP-ATG8a-labeling and LTR staining results showed the induction of autophagy in roots of young *A. thaliana* plants as a response to simulated microgravity. The maximal level of autophagosome formation in root cells was detected in clinostated plants on the 6^th^ day, which was followed by a gradual decrease of autophagosomes count on 9^th^ and 12^th^ days of experiment. The described findings were further confirmed statistically (Fig. 4). It has been determined that the number of autophagosomes (per cell) in clinostated plants was almost 2-fold increased on the 6^th^ day, compared to the control. A robust decrease of the number of autophagosomes was noted on the 9^th^ day, which was, however, significantly higher than the autophagosome counts in control plants. Despite that, on the 12^th^ day, the number of autophagosomes in the root cells of plants grown under clinostating conditions and in control plants was not significantly different. This confirms the observed gradual adaptation of plants to simulated microgravity, expressed in the decrease of autophagosome number in root cell within the later growth stages.

### The majority of *AtATG8* genes show similar expression patterns under autophagy induced by simulated microgravity

**Fig. 5:**
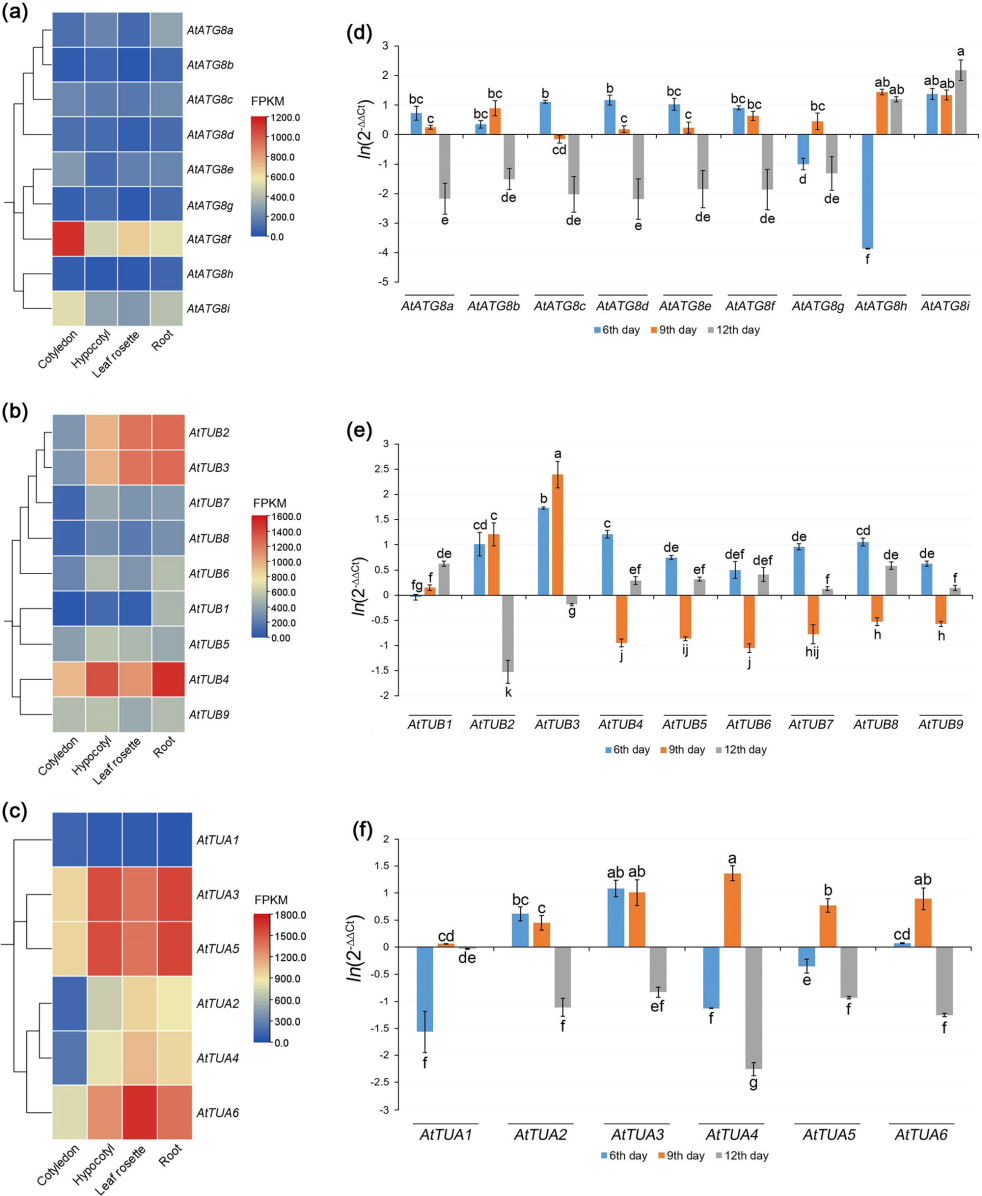
Expression of *ATG8*, *TUB* and *TUA* genes under normal conditions and simulated microgravity. Expression of *ATG8* (**a**)*, TUB* (**b**)*, TUA* (**c**) genes in *A. thaliana* under normal conditions in different tissues and changes in expression of *ATG8* (**d**)*, TUB* (**e**)*, TUA* (**f**) genes after 6, 9, and 12 days of growth under clinostating. Different letters (**a**, **b**, etc.) denote the significantly different datasets (at *p* < 0.05, Fischer LSD), error bars are SEM. Heatmaps (**a**−**c**) were generated based on the transcriptomic data for *A. thaliana*, obtained from a publicly available database (https://bar.utoronto.ca/eplant)^42^.

Since the ubiquitin-like protein ATG8 is crucial for successful autophagosome formation, we investigated the expression patterns of all 9 genes of ATG8 isotypes (ATG8a-i), present in *A. thaliana*, in order to better understand autophagy development under simulated microgravity (Fig. 5). Based on the previous research data (available in BAR ePlant database: https://bar.utoronto.ca/eplant)^42^, we found that under the normal conditions, the majority of the *AtATG8* are being higher expressed in roots (hypocotyl) (Fig. 5a). Only *AtATG8e*, *AtATG8f* demonstrate higher or comparable with roots expression in other tissues. Notably, *AtATG8i* shows change of its expression pattern during the development: it is being higer expressed in cotyledons, but upon the formation of vegetative leaf rosette the expression of *AtATG8i* becomes 2-fold higher in roots, rather than in stem.

The obtained here results suggest that different genes of ATG8 isotypes may be differentially expressed throughout the clinostating experiment (Fig. 5d). For the majority of these genes (six out of nine) the general pattern was following: moderate increase of the expression on the 6th day of growth, compared to control; slight increase of the expression (sometimes non-significant) on the 9^th^ day, followed by significant 3.7−8.8-fold decrease of expression of the 12^th^ day (in the range of *ln*(2^−ΔΔCT^) = −1.5 to −2.18, shown in Fig. 5d). To ensure reliable normalization, the housekeeping gene AtEFα was utilized in our analyses.

In particular, after 6 days of plant growth under simulated microgravity conditions, *AtATG8a*, *AtATG8b*, *AtATG8c*, *AtATG8d*, *AtATG8e*, *AtATG8f*, *AtATG8i* were significantly upregulated by 1.42−3.93-fold, compared to control (Fig. 5d). The greatest increase of expression on the 6^th^ day was recorded for *AtATG8i* (*ln* = 1.37) and paralogous *AtATG8c* and *AtATG8d*, increased by 3.03−3.21-fold (*ln* = 1.11−1.17). At the same time, *AtATG8g* and *AtATG8h* were found to be down regulated by 2.72- and 47.46-folds, respectively. Noteworthy, that these two genes were the only ATG8 genes downregulated on the 6^th^ day of growth under clinostating condition, while the *AtATG8h* showed the maximal downregulation (*ln* = −3.93), recorded for any of the *AtATG8* representatives.

At the 9^th^ day of cultivation, the expression of *AtATG8c*, *AtATG8d*, *AtATG8e*, *AtATG8g* in clinostated plants was not significantly different from control ones. The *AtATG8a* showed shallow, however significant, 1.28-fold increase of expression in experimental plants. *AtATG8b*, *AtATG8h* and *AtATG8i* showed similar increase by 2.43-, 4.22- and 3.81- fold, respectively (*ln* = 0.89−1.44, Fig. 5d). Later, on the 12^th^ day the expression of the majority of ATG8 genes was significantly decreased by 3.72−8.76-fold. Individual differences between downregulated *AtATG8a-g* were not significant on the 12^th^ day of the experiment. Remarkably, paralogous *AtATG8h* and *AtATG8i* were upregulated by the 12^th^ day of clinostating (*ln* = 1.19−2.18). Increase of the *AtATG8i* expression was the highest among all *AtATG8* at any stage and was increased by 8.85-fold, compared to control.

### Expression profiles of tubulin genes under simulated microgravity

The formation of autophagosomes is a complicated process which requires coordination of a number of proteins, including α- and β-tubulins^33,34^. In this study, the expression of all 15 *A. thaliana* α- and β-tubulin genes under simulated microgravity was also investigated (Fig. 5). Using the *A. thaliana* gene expression database^42^, we established that all β-tubulins showed predominant expression in hypocotyls (1.4−9.2-fold higher) at the early stages of the growth, while only *AtTUB9* was expressed on comparable levels in cotyledons and hypocotyls (only 5.4% higher in hypocotyl) (Fig. 5b). Upon the formation of leaf rosette the majority of β-tubulin genes were equally expressed in leaf rosette and roots. Only *AtTUB1*, *AtTUB4*, *AtTUB6* and *AtTUB8* were still higher expressed in the roots by 1.4−9.7-fold (Fig. 5b).

Unlike *AtTUA*, the vast majority of β-tubulin genes showed high similarity of the expressional patterns (Fig. 5e). The genes *AtTUB4-9* were equally upregulated at the 6^th^ day by 1.65−3.36-fold (only upregulation of *AtTUB4* differed from the group significantly), which was followed by significant 1.7−2.58-fold decrease of expression of these genes at the 9^th^ day, while on the 12^th^ day of clinostating these genes were, again, significantly higher expressed, 13.9−79.7% higher compared to the control. On contrary, *AtTUB2* and *AtTUB3*, which both are contained in common locus, showed different expression patterns in response to clinostating. Both genes were upregulated on the 6^th^ day, showing 2.75-fold increase of *AtTUB2* expression and 5.65-fold for *AtTUB3*, compared to the control (Fig. 5e). On the 9^th^ day the genes were even higher expressed demonstrating 3.34- and 10.97-fold increase of expression respectively, which was followed by 4.58- and 1.21-fold downregulation on the 12^th^ day. Remarkably, *AtTUB1* was the only the β-tubulin gene, which showed almost no change in expression on the 6^th^ day, being not significantly different from control, but later was upregulated by 16% and 86.7% on 8^th^ and 12^th^ day respectively (Fig. 5e).

As the result of investigating *A. thalaina* gene expression data in the mentioned above database^42^, all α-tubulin genes were found to be also predominantly expressed in hypocotyls 1.6−4.8-fold higher, than in cotyledons, with the exception of *AtTUA1*, which higher expressed in cotyledons (Fig. 5c). However, upon the formation of the leaf rosette only *AtTUA3* and *AtTUA5* kept higher expression within roots, while the expression of other α-tubulin genes was comparable or 12−25% lower than in vegetative leaf rosette (Fig. 5c).

No specific expressional patterns under clinostating were found for *AtTUA* panel. For instance, in 6 days of *A. thaliana* growth under clinostating conditions, it was found that *AtTUA1*, *AtTUA4* and *AtTUA5* genes were significantly downregulated compared to control. The expression of *AtTUA1* and *AtTUA4* was not significantly different, *ln* = −1.57 and −1.13, respectively (4.79- and 3.09-fold decrease on the 6th day), while the expression of *AtTUA5* remained higher *ln* = −0.35. On contrary, the expression of *AtTUA2* and *AtTUA3* significantly increased by 1.85−2.95-fold (Fig. 5f). At the same time the increase of *AtTUA6* expression was statistically significant, however, negligible – only 1.07-fold. Interestingly that group of paralogous genes had opposite expression dynamics. For instance, upregulation of *AtTUA3* expression was accompanied by decrease of *AtTUA1* and *AtTUA5* expression, while the downregulation of *AtTUA4* was observed along with the high expression of *AtTUA2*.

At the 9^th^ day clinostating all *AtTUA* genes were upregulated, however at the different level (Fig. 5f). Genes *AtTUA3*, *AtTUA4* and *AtTUA6* were upregulated at the same level, by 2.44−3.93-fold, while *AtTUA2* and *AtTUA5* were upregulated by 1.56-and 2.16-fold, respectively. The change of the *AtTUA1* expression was very small, however statistically significant, only increased by 6%, compared to control. After 12 days of cultivation under clinostating conditions the expression of all α-tubulin genes rapidly decreased (Fig. 5f). The most significant downregulation was recorded for *AtTUA4*, expression of which decreased by 9.55-fold (*ln* = −2.26). Another four genes, *AtTUA2*, *AtTUA3*, *AtTUA5* and *AtTUA6*, were downregulated at the same level, about 2.29−3.51-fold. The change of the *AtTUA1* expression was, again, very low – the gene was expressed only by 1.7% that in control (Fig. 5f).

### Expansion of ATG8 gene family in *A. thaliana*

At the next stage of our study, the evolutionary divergence of *AtATG8* genes was investigated, since particular genes may be represented by ancient or relatively young duplicates. This can have the direct impact on variation of their expression patterns since such divergence is one of the possible routes for the subfunctionalization of ohnologous genes.

**Fig. 6:**
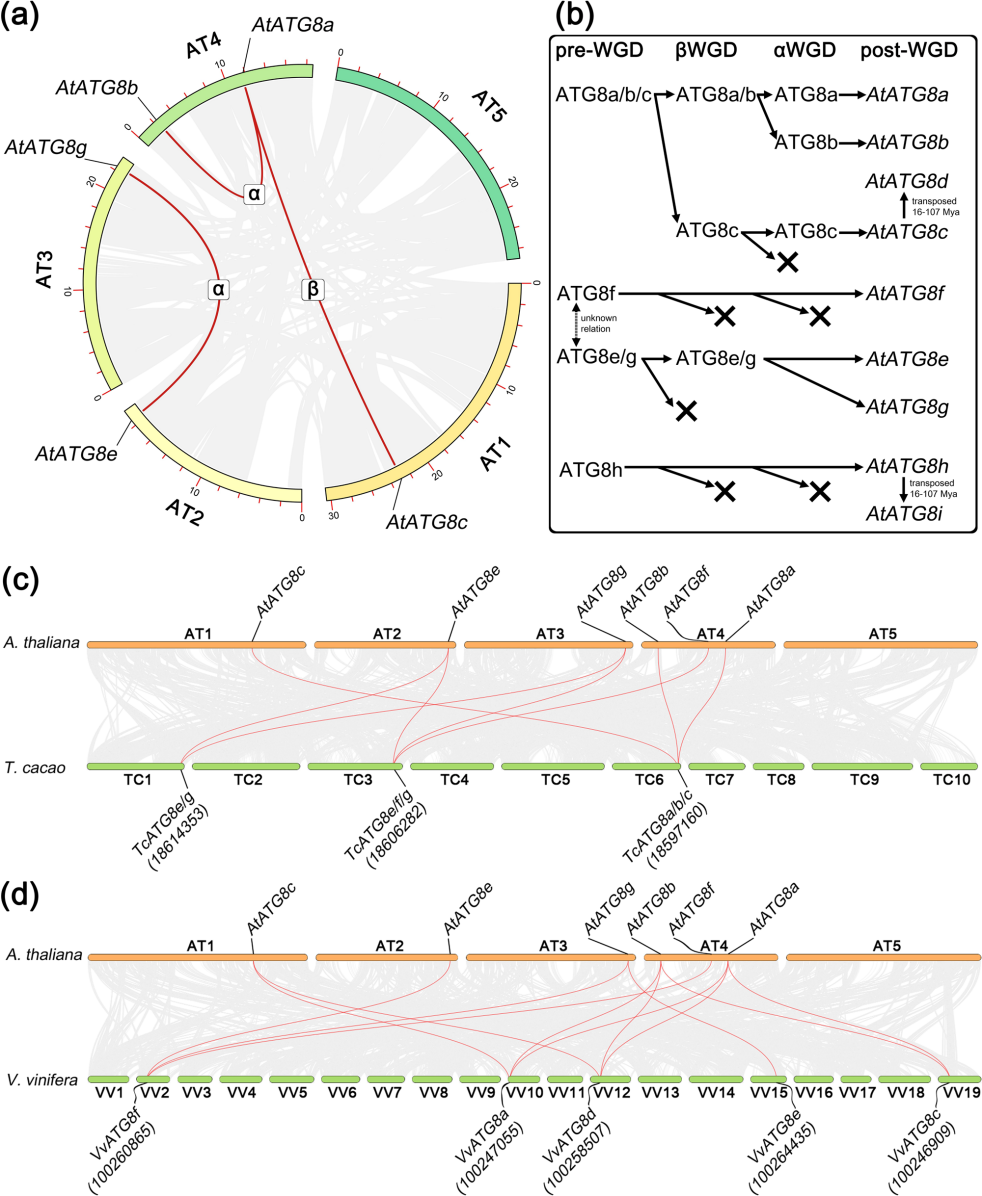
The evolutionary pathway of ATG8 genes family expansion in *A. thaliana.* **a** Interchromosomal synteny of *AtATG8* genes, syntelogs are dated to corresponding WGD event; **b** Reconstruction of possible evolution of *AtATG8* gene family in *A. thaliana*, based on the conducted analyses and previously published data; **c** Intergenomic synteny between *A. thaliana* and *T. cacao*; **d** Intergenomic synteny between *A. thaliana* and *V. vinifera*. Respective syntenic pairs are highlighted with red threads, NCBI ID of *T. cacao* and *V. vinifera* ATG8 genes are given in rounded brackets.

Firstly, the interchromosomal synteny analysis, involving *A. thaliana* genome, was performed (Fig. 6a). In general, three *ATG8* syntelog pairs were found within the genome of *A. thaliana*. Among them, three genes formed two syntenic pairs: *AtATG8a*-*AtATG8b* and *AtATG8a*-*AtATG8c*, while *AtATG8e* and *AtATG8g* formed the third pair. Formation of these syntelog pairs was assigned to a particular whole genome duplication (WGD) event, based on the previously published data on the duplication history of *A. thaliana* genes^43^. In particular, divergence between *AtATG8c* and *AtATG8a*-*AtATG8b* was dated to the β-WGD event, whereas the divergence between *AtATG8a* and *AtATG8b* – to the more recent α-WGD event. Thus, it may be concluded that all three mentioned genes appear to be the ancient ohnologs that evolved from a common ancestral gene, which is reflected in Fig. 6b.

The divergence of the other pair, *AtATG8e*-*AtATG8g* was also dated to the α-WGD event, while other possible paleoohnologs, resulting from the β-WGD event, have not preserved to the date (Fig. 6b). Other *AtATG8* genes emerged as paralogs, but not as the result of α- or β-WGD events. Such genes as *AtATG8d* and *AtATG8i* were recognized as transposed duplicates of *AtATG8c* and *AtATG8h*, respectively. Moreover, *AtATG8d* and *AtATG8i* were not identified as syntelogs during the interchromosomal synteny analysis (Fig. 6a), since they were not contained in any genomic block (with minimal size of 5 genes), collinear to the loci of *AtATG8c* and *AtATG8h*. These findings indicate that *AtATG8d* and *AtATG8i* originated as locally duplicated paralog, apart from any WGD, segmental or tandem duplication events. Origin of these paralogs was dated to 16−107 mya, according to previously published data^43^. Thus, *AtATG8d* likely emerged after α-WGD event, taking in account that this gene originates from relatively young *AtATG8c*.

The described findings are supported by results of intergenomic synteny, performed for *A. thaliana* - *Theobroma cacao* and *A. thaliana* - *Vitis vinifera* (Fig. 6c, d). Particular species represent genomes, which have not faced the mentioned α- and β-WGD events, specific for Brassicaceae. In this case, *T. cacao* represents the closest non-Brassicales relative, while *V. vinifera* – basal relative to Rosids clade. It was found that each of *AtATG8a, AtATG8b* and *AtATG8c* genes form syntenic pairs with one ATG8 gene in *T. cacao* genome – *TcATG8a/b/c* (NCBI gene ID - 18597160), which further confirms common origin of these ohnologous genes in *A. thaliana* (Fig. 6c). Interestingly, genes *AtATG8e, AtATG8f* and *AtATG8g* formed syntenic pairs with *TcATG8e/f/g* (18606282, located on TC3 chromosome), while only two of them (*AtATG8e* and *AtATG8g*) were syntelogs for *TcATG8e/g* (18614353, TC1 chromosome). The second case is clearly explained by joint origin of *AtATG8e* and *AtATG8g*, resulting from α-WGD. On the other hand, the discovered syntelog status of *AtATG8f* for *AtATG8e* and *AtATG8g* points out that the divergence between ancient *ATG8f* and *ATG8e/g* may have occurred prior to the speciation of Brassicaceae clade, resulting from earlier duplication event (possibly γ-WGD).

Similar results were observed during the comparison of *A. thaliana* - *V. vinifera* genomes, except the fact that *V. vinifera* contained five *ATG8* syntelogs (Fig. 6d). In particular, *VvATG8a* (100247055) and *VvATG8d* (100258507) formed syntenic pairs with all three ohnologous *AtATG8a, AtATG8b* and *AtATG8c*, while *VvATG8c* (100246909) was syntelogous only for two of *A. thaliana* ATG8 genes – *AtATG8a* and *AtATG8b*. Other syntelog pairs were formed between *VvATG8f* (100260865) and *AtATG8e, AtATG8g*, and, again, *AtATG8f*. This finding additionally evidences for the common origin of *AtATG8e, AtATG8g*, *AtATG8f*. An additional pair of syntelog was formed by *VvATG8e* (100264435) and *AtATG8g*, which indicate that the later gene may be closer to its ancestral form of *AtATG8e/g*.

Such unequal syntelog recognition may indicate that some of *V. vinifera ATG8* genes might be closer to common ancestral *ATG8a/b/c* and *ATG8e/g* (Fig. 6b). It is also unclear whether the *ATG8* genes of *V. vinifera* were duplicated independently. Notably, none of the transposed duplicates *AtATG8d* or, *AtATG8i* were recognized as syntelogs of any of *T. cacao* or *V. vinifera* ATG8 genes. Finally, no syntelogs were found for *AtATG8h*, thus its origin remains unclear.

### Divergence among α- and β-tubulin gene panels in *A. thaliana*

**Fig. 7:**
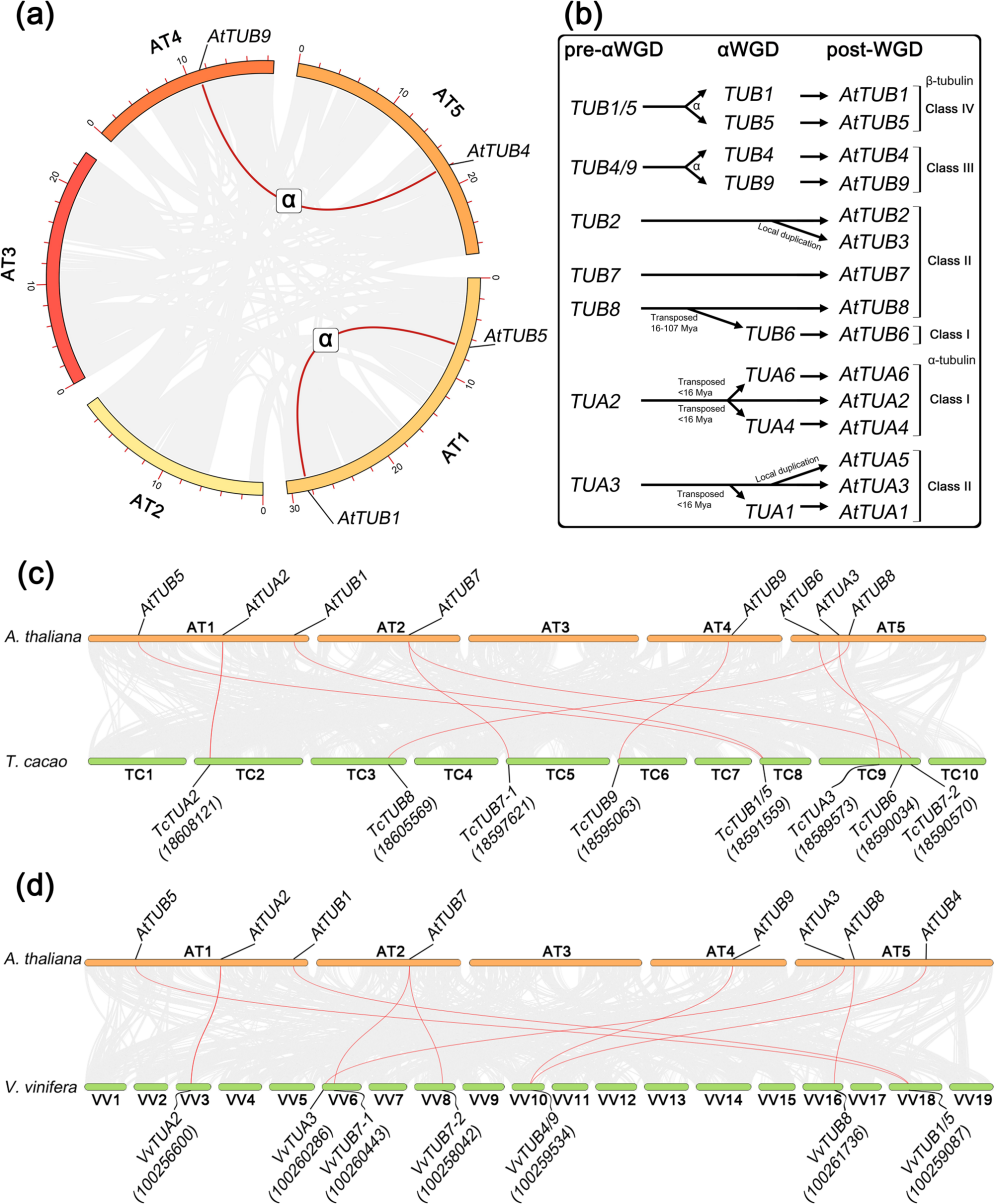
The evolutionary pathway of α- and β-tubulin gene subfamilies expansion in *A. thaliana.* **a** Interchromosomal synteny of *AtTUB* genes, syntelogs were defined as resulting from α-WGD event (no such pairs for *AtTUA* were found); **b** Reconstruction of possible evolution of *AtTUA* and *AtTUB* genes in *A. thaliana*, based on the conducted analyses and previously published data (phylogenetic Classes of α- and β-tubulins were assigned, based on our previously published data). **c** Intergenomic synteny between *A. thaliana* and *T. cacao*; **d** Intergenomic synteny between *A. thaliana* and *V. vinifera*. Respective syntenic pairs are highlighted with red threads, NCBI ID of *T. cacao* and *V. vinifera* tubulin genes are given in rounded brackets.

In parallel, we have investigated the evolutionary divergence of all α- and β-tubulin genes, in order to explain their diversity and clarify the nature of expression pattern difference/similarities. The interchromosomal synteny analysis of *A. thaliana* genome revealed revealing two syntelog pairs of β-tubulin genes: *AtTUB1*-*AtTUB5* and *AtTUB4*-*AtTUB9* (Fig. 7a). The origin of these ohnologous pairs was dated to the α-WGD event based on the previously reported data on gene divergence dates in *A. thaliana*^43^, which is reflected in Fig. 7b. Noteworthy, *AtTUB1*-*AtTUB5* and *AtTUB4*-*AtTUB9* gene pairs are the only palaeoohnologs within the α- and β-tubulin gene sets, while the other gene duplicates are paralogs.

*AtTUB2* and *AtTUB3*, as well as *AtTUA3* and *AtTUA5*, are local duplicates (paralogs), sharing the same loci (genes are adjacent to each other). The further synteny analyses of *A. thaliana* genome with *T. cacao* and *V. vinifera* (Fig. 7c, d) have not revealed presence of such paralogous pairs in these two species. This suggests that *AtTUB2*-*AtTUB3* and *AtTUA3*-*AtTUA5* might be a specific Brassicacae feature (or specific to a wider phylogenetically related group). *AtTUB7* was the only gene for, which duplicates were not present in *A. thaliana*. However, *T. cacao* and *V. vinifera* genomes contained two syntelogs each: *TcTUB7-1* (18597621) and *TcTUB7-2* (18590570), as well as *VvTUB7-1* (100260443) and *VvTUB7-2* (100258042). Interestingly, *AtTUB6* appears to be a transposed duplicated of *AtTUB8*, while the two genes belong to distinct phylogenetic classes of β-tubulin^44^. Syntelogs (most likely orthologs) have been identified within the *T. cacao* genome: *TcTUB6* (18590034) and *TcTUB8* (18605569). At the same time, *V. vinifera* genome contains only *AtTUB8* ortholog – *VvTUB8* (100261736), while *AtTUB6* was not recognized as the syntelog of *VvTUB8*.

At the same time, each class of α-tubulin isotypes was almost completely comprised of paralogous genes. For instance, *AtTUA4* and *AtTUA6* (Class I of α-tubulins) were defined as transposed duplicates of *AtTUA2* (Fig. 7b). No syntelogs for these duplicates were found neither in *T. cacao* genome, nor in *V. vinifera* (Fig. 7c, d). At the same time, interchmosomal synteny analysis of *A. thaliana* genome has shown that the α-tubulin genes of the Class I did not form any syntenic pair. These α-tubulin genes did not share the same genomic landscape and were flanked by different non-homologous genes, therefore they were not recognized as syntelogs under the certain conditions of the analysis (min. block size of 5 genes). The same was observed in the case of other α-tubulin Class II member, *AtTUA1*, which was also identified as the transposed paralog of *AtTUA3* (Fig. 7b). The obtained results suggest that *AtTUA4*, *AtTUA6* and *AtTUA1* most likely have originated via the duplication of their ‘parental‘ gene solely, rather than via the duplication of certain genomic region, since these paralogs are observed out of their genomic context.

## Discussion

The results of our study show that simulated microgravity affects the growth and development of *A. thaliana* plants. Morphological analysis shows that although clinostating does not affect seed germination and does not cause significant morphological deviations, it leads to disorientation of root growth due to a constant change in the gravity vector. The main disturbances in the morphology and development of the root were observed on the 6^th^ and partially on the 9^th^ day; further, no significant deviations in the development of the primary roots, lateral roots, and root hairs were observed, which indicates the involvement of adaptation mechanisms in response to stress caused by clinostating. Previously space flight experiments demonstrated that the absence of gravitation vector leads to shortening and twisting of *A. thaliana* roots^45^. Our experiments revealed the skewing and waning response of *A. thaliana* roots to clinostating, which had been also shown^18,46^.

Growth and morphology alterations of *A. thaliana*, caused by microgravity, can affect various cellular processes, and also may determine a specific regulation of autophagy in plants, as originally suggested^18,47^. Experiments on *A. thaliana* seedlings under the simulated altered gravity on Earth showed similar effects on plant growth to those observed in space^48,49^. They also revealed significant transcriptomic and proteomic changes in the plants exposed to the altered gravity. Particularly, the changes were identified within the genes (and proteins) involved in various cellular processes, such as responses to various abiotic stress and cell cycle regulation under such conditions^50^.

Based on these previous findings, we also investigated changes in the expression of the important autophagy genes (*AtATG8* gene family) and the key MT protein genes (α- and β-tubulin subfamilies) under simulated microgravity. In the experiments described here, three lines of *A. thaliana* were used, two of which express the chimeric genes *GFP-MAP4* and *GFP-ATG8A*. It is noteworthy, that plant lines expressing such chimeric genes are very simple and appropriate models for studying intracellular structures and their functional state in living cells under microgravity conditions^51^. These two lines (GFP-MAP4 and GFP-ATG8a) were used in a more detailed study of the role of MTs in the development of autophagy in plant cells. MTs are known to be responsible for maturation and movement of autophagosomes through dynamic changes in their state^52^. Experiments in which *A. thaliana* plants were exposed to various stresses, in particular, cold, heat, salt, and UV radiation, have already been conducted to investigate the relationship between the development of autophagy and cytoskeleton functioning in response to stress^34,53–55^. However, there is still a shortage of data on the role of MTs in the development of autophagy in plant cells under microgravity.

Since the growth and development of plants also depend on the correct organization and functioning of the cytoskeleton, and in particular MTs, we studied the effect of clinostating on organization and dynamics of MTs in living root cells. Our experiments with *A. thaliana* GFP-MAP4 line demonstrated that MT orientation in root cells changes or MTs become disorganized after 6 days of exposure to simulated microgravity, which correlates with visible significant changes in plant morphology. However, after 9−12 days, no dramatic disturbances in the orientation and organization of MTs were found, which might be due to adaptation of the plants to clinostating at certain level. These data further support the evidence that MTs, being an intracellular target, are directly involved in the microgravity response. Noteworthy, re-orientation of MTs may affect assembly and overall movement of autophagosomes, since this cytoskeletal structure often play an important role in trafficking of such vesicles.

One of the approaches for studying autophagy development is to observe cell acidity, since lysosomes/vacuoles fuse with autophagic cells during autophagy. For this purpose, we used LTR, a dye, which stains acidic cellular compartments and is commonly used in autophagy studies^56^. As previously shown, this dye can be used as evidence of autophagic cargo delivery to lysosomes^57,58^. Moreover, LTR has been used to provide correlative data on autophagy in *D. melanogaster* fat body cells^59^. Our data, obtained using LTR, showed that active autophagy processes occur under simulated microgravity after 6−9 days. The LTR-staining itself may indicate vesicles, other than autophagosomes, however, its combination with other approaches is considered enough precise and reliable^60^. Previous studies have demonstrated that additional approaches, such as lines expressing GFP-ATG8/LC3, should be used to confirm the results obtained with acidotropic dyes^22,58,61^. Thus, using *A. thaliana* GFP-ATG8a transgenic line to study the development of autophagy in plants under clinostating conditions, we showed the active development of autophagy after 6 and 9 days. This GFP-ATG8a line was previously used in a study that demonstrated the role of autophagy in chloroplast degradation^60^. In addition, previously we have reported the similar results using MDC-staining, which are consistent with the described here findings and further confirms the observed effects of clinostating on the autophagy development^38^.

It should be noted that studies of the effect of microgravity on the induction of autophagy in plants have not been conducted earlier. It is only known that Ryu et al. studied autophagy under microgravity using HEK293T cells stably expressing GFP-LC3^47^. They found an induction of autophagy after 72 h of clinostating. Summarizing the results of our microscopic analysis using the GFP-ATG8a line and LTR-staining, we can conclude that the most active induction of autophagy occurs after 6–9 days under clinostating, while after 12 days we observed a decrease in the number of autophagosomes, indicating an important role of autophagy processes under the influence of simulated microgravity. We can assume that such a decrease in the development of autophagy (similar to the control) after a prolonged period of cultivation under simulated microgravity may be related to the plant adaptation to the absence of a stable gravity vector.

Along with that, here we proposed to analyse expression of the *AtATG8* and tubulin genes, accounting the context of their evolutionary origin, in order to explain the observed similarities/differences in expression patterns. Recent phylogenetic studies suggested that plant ATG8 proteins could be dissected into three major groups^62^. These groups include *AtATG8a-d*, *AtATG8e-g*, and *AtATG8h-i*, respectively. Later, it was shown that *AtATG8a-d* and *AtATG8e-g* are phylogenetically closer to each other, rather than to *AtATG8h-i*. At the same time, *AtATG8a-d* is the youngest ATG8 group, which is present in angiosperms only, while the other two may be found within mosses, ferns, and gymnosperms^13^. Interestingly that ATG8s of only *AtATG8e-g* group are present in green algae, whereas in fungi only proteins of *AtATG8h-i* group can be found.

Our findings are consistent with the described above results. The synteny analysis suggests the joint origin of *AtATG8a-d* genes via the series of WGD-events and duplication with transposition. The common origin of *AtATG8h-i* genes was also confirmed, as well as the origin of *AtATG8e* and *AtATG8g* via the WGD, while the origin of *AtATG8f* remains unclear. Most likely, *AtATG8f* arose from earlier WGD event (e.g., γ-WGD) or as a result of ancient local duplication. Previous attempts to unravel the origin of *AtATG8a-d* genes resulted in describing these genes as segmental duplicates, which was based on the assessment of *A. thaliana*-*V. vinifera* synteny and phylogeny reconstruction with very restricted number of taxa (arabidopsis, grape and rice only)^63^. Our revision, involving wider synteny analyses and other previously published data^43^, suggests that the expansion of *AtATG8a-d* groups was mostly driven by series of WGD-events.

More detailed phylogenetic revision of *ATG8* genes among flowering plants revealed that potentially orthologous genes to *AtATG8h-i* are present within Poacaeae (namely Clade II of ATG8), while the *AtATG8a-d* and *AtATG8e-g* groups (Clade I) were placed as the sister clade to Poaceae ATG8^20^. It was also shown that Poaceae have specific lineage of ATG8s, which appear to be basal to all ATG8s of dicots from Clade I. Such findings suggest that evolution of ATG8 family was highly independent within monocots and dicots, while later tend to have a broader panel of these genes. Overall, autophagy-study results, obtained with model species like *A. thaliana* should be extrapolated very carefully on a phylogenetically distant species, due to the complex evolution of such genes, as *ATG8*s.

More evidence has recently been obtained regarding the role of the ATG8 protein in the formation of autophagosomes^13,21^. The pattern of *AtATG8* gene expression in *Arabidopsis* under different abiotic stresses has also been published recently^64^. We also investigated earlier changes in *AtATG8* gene expression in *A. thaliana* under starvation, salinity, and osmotic stresses^33^. Comparing the results obtained in this study, we found that the members of Clade I (*AtATG8a-g*) showed highly similar expression patterns. Particularly, six genes (*AtATG8a-f*) demonstrated somehow identical dynamics of expressional changes within the experiments: upregulation on the 6^th^ day, lower upregulation, or non-different expression from the control on the 9^th^ day, followed by significant downregulation on 12^th^ day. Only *AtATG8g* differed from the group, showing the significant downregulation on the 6^th^ day. Representatives of Clade II (*AtATG8h-i*) showed rather different expression within the experiment. Both genes were significantly upregulated on the 9^th^ and 12^th^ days, while on the 6^th^ day the genes were differentially expressed: *AtATG8h* was dramatically downregulated by 47.5-fold, while *AtATG8i* was expressed on higher levels, comparing to the control. Interestingly that, despite the duplicated nature of *AtATG8* genes, they were expressed almost identically within their evolutionary groups. Overexpression of *ATG8a, ATG8b, ATG8h, ATG8i* genes was fixed under carbon starvation^32^ as well as a similar pattern of gene expression was obtained under microgravity conditions.

Previously, it was shown that *AtATG8f* and *AtATG8h* are crucial for maintaining the autophagic activity under phosphate starvation, while loss of these genes leads to an increased ATG8 lipidation^65^. Surprisingly, both genes showed similar expression patterns in the root stele under phosphate starvation, despite they belong to different lineages. Therefore, microgravity can induce autophagy, which is beneficial for plants trying to adapt to stress conditions. Considering ATG8 as an obligate structural part of an autophagosomes, we can assume that *ATG8* genes are involved in the development of autophagy under microgravity conditions in the same way.

Classification of the tubulins greatly relies on the early phylogenetic studies of this gene family^44^. It is widely accepted that α-tubulins in *A. thaliana* are represented by two classes, while more diverse β-tubulins may be grouped into four or five distinct classes^44,66,67^, based on their phylogeny. However, this view often does not allow to sufficiently explain the diversity of tubulin genes in the other phylogenetically-distant species, e.g., *Prunus* sp.^68^. Here, we revealed that the observed tubulin diversity in *A. thaliana* may be highly specific for this particular taxon, or Brassicaceae family generally.

Our results suggest that a major part of the tubulin isotype diversity, observed within the classes, arise from WGD and gene duplication events, specific to the *A. thaliana* (and, possibly, to related species). For instance, α-tubulin Classes I and II are comprised only out of paralogous genes, which arose less than 16 Mya and do not have orthologs in phylogenetically distant species, like *T. cacao* or *V. vinifera*. Similarly, β-tubulin Classes III and IV arose as groups of ohnologous genes in *A. thaliana*. The members of these Classes appear to be sub-orthologs in relation to Class II and IV β-tubulins in *T. cacao* and *V. vinifera*. Along with that it was established that β-tubulin Class I arises directly from the TUB8 isotype gene (Class II) via the ancient duplication event, which explains the joint grouping of these Classes in numerous phylogeny reconstructions^44,66–68^. Presence of β-tubulin Class V^44^, or Class I-like^67–69^ remains a mystery. Representatives of this ambiguous Class are absent in *A. thaliana*, but present in Rosaceae (*Prunus* sp.)^68^, Salicaceae (*Populus trichocarpa*, *Salix arbutifolia*)^44,69^, Fabaceae (*Medicago truncatula*)^44,69^, Myrtaceae (*Eucalyptus grandis*)^68,69^, Linaceae (*Linum usitatissimum*)^67,70^, Malvacaeae (*Gossypium* sp.)^44,71^ and, possibly, in Poaceae (*Zea mays*, *Hordeum vulgare*, *Triticum aestivum*, *Oryza sativa*)^44,72,73^. However, this group of β-tubulins could be comprised out of the genes, orthologs of which are absent in *A. thaliana* genome.

We have not observed any common expression pattern for *AtTUA* paralogous genes under clinostating treatment. Such genes tended to have different expression on early stages of autophagy development: upregulation of *AtTUA3* was accompanied by decrease of its paralogs, *AtTUA1* and *AtTUA5*, expression, while the downregulation of *AtTUA4* was observed along with the high expression of *AtTUA2*. Such differences suggest that *AtTUA* gene duplicates are being highly specialized, at least in terms of their expression. Interestingly, the expression of *AtTUA1* was significantly decreased by the 6^th^ day of the experiment, while later it was not different from the control. Such unusual expression pattern may be related to the *AtTUA1* function, since this gene almost exclusively expressed in pollen and during the pollen tube growth^44^ and was shown here to be shallowly expressed in the roots.

On the other hand, six out of nine β-tubulin genes showed similar expression patterns. Ohnologous *AtTUB4* and *AtTUB9*, paralogous *AtTUB8* and *AtTUB6*, as well as the other two genes, *AtTUB5* and *AtTUB7*, showed upregulation at the 6^th^ day, downregulation at 9^th^ day, followed by less robust upregulation at 12^th^ day. On the contrary, ohnologous *AtTUB1* and *AtTUB5* showed different expression dynamics within the experiment. At the same time, tandem duplicates *AtTUB2* and *AtTUB3* showed the most significant differences in expression, compared to the control, and shared the same expression pattern. Since these two genes are contained in common loci, they might be jointly regulated by the same promoter region, however such statement requires further investigations. The observed expression similarity of numerous β-tubulin duplicate genes might have a particular adaptation and evolutionary advantage since the sequences of these genes retain highly identical (over 95% similarity). Similar effects were observed in flax, β-tubulin duplicates of which commonly were divergent in sequences and shared similar expression rates in same tissues^67^. It was also suggested that availability of diverse tubulin isotypes may serve as an intracellular pool of monomers, suitable for precise setup of MTs, specialized for particular vesicle transport^67^. Such ‘tuning’ of MTs could be also performed via the introduction of post-translational modifications^74^. Earlier we have shown that the increased level of acetylated α-tubulin K40 are observed in plant autophagic cells^75^.

Although the details of relationships between functioning of MTs and autophagy development is still under investigation, it has already been shown that microgravity can influence cytoskeletal architecture and promote functional cellular remodeling^76,77^. Recent research suggests that the cytoskeleton may be the first sensor of microgravity^78^. As MTs are important regulators of organelle positioning and movement, they can also be involved in functioning of autophagosomes in cells. Currently, it is not well known how MTs interact with autophagosomes: through ATG8, motor proteins or any other microtubule-associated proteins.

The results of the expression analysis potentially confirm the interplay between the structural units of autophagosomes like ATG8 and MTs. In particular, our data demonstrate relationship between MT function and autophagy development as we observed similar expression profiles for *AtATG8b, AtATG8f*, and *AtTUA2, AtTUA3*, and *AtTUB2* and *AtTUB3*, which all showed upregulation on 6^th^−9^th^ days with the decrease of expression on 12^th^ day. It should be noted that somehow all *AtATG8a-f* might be included into the mentioned group, however, *AtATG8a*, *AtATG8c*, *AtATG8d*, *AtATG8e* showed no significant difference in expression on the 9^th^ day. It is also worth noting that *AtTUA4*, *AtTUA5* and *AtTUB4-9* showed opposite dynamic of expression changes. For instance, the α-tubulin expression showed ‘down-up-down’ pattern on the reference days (6^th^, 9^th^, and 12^th^), while the mentioned β-tubulin genes possessed the opposite ‘up-down-up’ pattern of expression.

We recently reported co-expression of *AtTUA1* and *AtATG8e* under glucose starvation, *AtTUA3* and *AtATG8f* under salt stress, and *AtTUA3, AtATG8f*, and *AtATG8e* under osmotic stress^33^, which also indicates the specificity of these protein pairs in developing autophagy induced by various stimuli^79^. The involvement of β-tubulin in the induction of autophagy is still poorly understood, as β-tubulin genes have been widely used as housekeeping genes^80,81^. Noted differential co-expression of various *ATG8* and tubulin genes could be related to precise tuning of MT and autophagic systems to each other for the proper functioning. The findings on the expression of β-tubulin genes require further in-depth investigation, as their expression profiles differ from those of the *TUA* genes. It remains unclear why the expression of the six β-tubulin genes increased again after prolonged simulated microgravity, although this was not observed for any of the *TUA* genes. It can be assumed that this is due to the fact that β-tubulin is involved in a number of post-translational modifications necessary for binding of MAPs or motor proteins, as well as they might modulate the function of MTs in response to extracellular stimuli^82^. It also may be assumed that the potential interplay between cytoskeletal and autophagy-related protein genes could be associated with lipid metabolism, lipophagy in particular. For example, MTs being the tracks for vesicle transport are involved MTs in lipid (lipid droplets) transport to the tip of the grown pollen tube^83^, where the triacylglycerols are being degraded via lipophagy^84^. Moreover, it has been shown that lipophagy is involved in early seedling development in *A. thaliana*^84^, while loss of particular ATG8 genes may lead to increase of lipidation under certain conditions^65^. Even if MTs and autophagic system are not interact directly, their interplay is important for successful passage of crucial plant development stages.

By evaluating the expression profiles of all the genes studied in this work, it can be argued that *AtATG8b, AtATG8f*, and *AtTUA2, AtTUA3*, and *AtTUB2* and *AtTUB3* genes are co-expressed in response to clinostating exposure. We hypothesize that these genes may be key elements in plant adaptation to long-term exposure to microgravity. These findings provide a key basis for further studies of cellular mechanisms of autophagy and involvement of cytoskeletal structures in autophagy biogenesis under microgravity, which would enable development of new approaches, aimed on enhancing plant adaptation to microgravity.

The results obtained in this study clearly demonstrate that simulated microgravity affects plant growth and induces autophagy development in *A. thaliana* cells. We can suggest that ATG8 proteins, a structural unit of autophagosomes, are directly involved in the development of microgravity-induced autophagy in plant cells during the first 6−9 days of the clinostating experiments. Since plant growth and development are directly dependent on the correct functioning of the cytoskeleton, we found significant changes in MT organization and in the gene expression profiles of α- and β-tubulin under simulated microgravity. The results of a molecular analysis of six isotypes of α-tubulin and nine isotypes of β-tubulin show that some of these genes can be identified as being more specific in the implementation of autophagy under these stress conditions. In particular, we found the most pronounced increased expression pattern of *AtATG8b, AtATG8f*, and *AtTUA2, AtTUA3, AtTUB2* and *AtTUB3* genes in response to clinostating. It should be noted that the main responses to stress were established after 6−9 days, whereas after 12 days an adaptive response to microgravity-induced stress was observed in *A. thaliana*.

The obtained data are the basis for further research into the cellular mechanisms of involvement of different ATG8 isotypes and their interaction with other molecular components, particularly MTs, in the development of autophagy under microgravity conditions. Identification and characterization of these proteins provide a basis for molecular understanding of autophagy processes in plants. By using various drugs that can affect the polymerization/depolymerization of MTs, post-translational modifications of tubulin, the functioning of motor proteins, as well as the development of autophagy processes, it is possible to increase the resistance of plants to microgravity stress and improve the growth of plants on board the space station.

## Methods

### Plant material and growth conditions

*A. thaliana* ecotype Columbia-0 (Col-0), two transgenic lines: GFP-ATG8a **(**stock number CS39996**)** (Allison Thompson, 2005) and GFP-MAP4 **(**stock number CS799990**)** were used in this study. Seeds of these lines were obtained from the Nottingham Arabidopsis Stock Centre (NASC, UK). Surface sterilization of *A. thaliana* seeds was performed with a 10% solution of NaOCl and 0.5% Tween-20 for 15 min, followed by washing five times with sterile water.

Seedlings of *A. thaliana* lines were grown under sterile conditions on a half-strength MS medium^85^ containing vitamins (Duchefa, Haarlem, Netherlands), supplemented with 10 g/L sucrose and solidified with 4 g/L Gelrite (Duchefa) at pH 5.7. After overnight incubation at 4 °C^82^, the dishes were placed vertically in a climate chamber at 24 °C in a 16/8 h (light/dark) photoperiod^86^ and also grown on a horizontal clinostat (4 rpm), as described by us earlier^18^.

### Measurement of *A. thaliana* root growth

To study the microgravity effects on root growth and morphology, *A. thaliana* seedlings were analyzed after 6, 9, and 12 days of exposure. Series images of growing *A. thaliana* seedling were taken by Canon Power Shot G6 digital camera (Canon, Taiwan) in the macro mode.

Plant root length, root hairs and cell area were measured with Image J software (version 1.38d; http://rsb.info.nih.gov/ij/). The results were recorded as the mean ± standard deviation of the triplicate experiment. Calculations of root growth rates were done as described earlier^87^. Average angular declination, gravitropic index (GI), vertical growth index (VGI) and HGI were calculated as described by *Villacampa* et al.^39^. The comparison between the root length measurements was evaluated with a student *t* test. Experiments were repeated at least three times with 30 seedlings for each time point.

### Comparative genomics analyses

The analysis of infragenomic (interchromosomal) synteny of *A. thaliana* genes was performed in TBtools v1.0971 software^88^, using the MCScanX algorithm^89^. The most recent genome assembly for *A. thaliana*, available in NCBI database – TAIR10.1 (GCA_000001735.2), was used. Identification of the syntenic pairs was conducted, based on the translated protein sequences of *A. thaliana* genes, with minimal block size – 5. In order to reveal potential singleton duplicated genes (independently transposed duplicates), a database, containing list and history of *A. thaliana* genes duplication, was used^43^. The results of both analyses were correlated to identify the nature of identified syntelogs (local, segmental, α- or β-WGD duplicates). The results were further visualized as circos plots.

In order to explore the syntenic relationships of *AtTUB*, *AtTUA*, *AtATG8* genes with their potential orthologs in other species, additional intergenomic synteny analyses were performed, using the genomes of *Theobroma cacao* (GCA_000208745.2) and *Vitis vinifera* (GCA_000003745.2), which both are believed to have lower level of autopolyploidy, than *A. thaliana*^90^. The intergenomic synteny analysis was performed using same algorithm and software, as for infragenomic one.

The results of both infra- and intergenomic synteny analyses were used to reconstruct potential evolutionary pathways of *TUB*, *TUA*, *ATG8* gene families expansion in *A. thaliana*.

### RNA isolation and RT-qPCR

Total RNA was isolated from 6, 9, and 12-day-old *A. thaliana* (Col-0) seedlings by innuSOLV RNA Reagent (Analytik Jena AG, Germany) according to the manufacturer’s instructions. The quality of isolated RNA was checked in a 1% agarose gel with formamide. The RNA concentration and purity were measured using NanoDrop Lite UV-Vis (Thermo Scientific, USA) and adjusted to 100 ng/μl. Reverse transcription reactions were performed with Revertasa Máxima (Thermo Fisher Scientific, USA), 5x solution for PCR, including 5 mM dNTPs, 2.5 mM MgCl_2_ (Ukrgentech, Ukraine), 1 μl oligonucleotides OligodT(18) (Thermo Fisher Scientific, USA), 500 ng of total RNA, and water free from RNase. PCR reaction was performed by Biorad CFX96 (USA). All samples were collected at least in triplicate from biologically independent plant material.

### Tubulin and *ATG8* gene expression analysis

The reaction mix for PCR-RT was composed of 2X Magic SYBR Mix (Procomcure Biotech, Austria), 50 ng of cDNA, 0.5 μl of reverse and forward primers with the final concentration of 10 pmol each. The reaction was performed in Biorad CFX96 USA under the following conditions: initial denaturation at 94 °C for 12 min; 44 cycles of amplification (94 °C for 15 s; 55−65 °C for 10 sec; 72 °C for 40 s), and final elongation at 72 °C for 10 min. The design of primers was carried out taking into account the following criteria: GC content ~40−60%; absence of non-specific secondary structures, e.g., hairpins and dimers; close annealing temperatures of the primer pair. Additionally, pairs of discriminative primers were designed not to target paralogous or ohnologous genes. The list of primers is provided in the Supplementary Table 1, while the primer annotations on the *ATG8*, *TUA* and *TUB* sequence alignments are listed as Supplementary Notes 1–3, respectively. Elongation factor α (*AtEFα*) was assessed as a control for RT-qPCR analysis. Relative expression values were calculated as 2^−ΔΔCT^. Up- or downregulation of the investigated genes were further represented as natural logarithm of relative expression (*ln*(2^−ΔΔCT^)). The mean value and standard error of mean were determined for each sample. At least five biological replicates were made to study each gene.

### Study of MT organization and autophagosome localization

Changes in the primary and lateral roots of 6, 9, and 12-day-old *A. thaliana* seedlings were detected using a light microscope Axioscope 40 (CarlZeiss, Germany), equipped with lenses Plan-Neofluar 10x/0.30, 20x/0.5 and 40x/1.30 OilDIC. Visualization of autophagosomes and changes in the MT organization upon microgravity were conducted in vivo using transgenic *A. thaliana* lines *GFP-ATG8a* and *GFP-MAP4*, respectively. The fluorescent signal from GFP was detected with a confocal laser scanning microscope (LSM 510 META, Carl Zeiss, Germany) and 40× Plan-Apochromat oil-immersion objective using the 488 line of the Argon laser (excitation 488/543 nm; emission 505/530 nm).

To study intracellular localization of autophagosomes, the root cells of *A. thaliana* seedlings were treated with LysoTracker™ Red DND-99 (LTR, Invitrogen, USA). The cells were incubated with 1 µM LTR at 37 °C for 1 min and then washed with phosphate-buffered saline (PBS, pH 7.4) three times^57^. The fluorescent signal from LTR was visualized with LSM 510 META using the 543 line of the HeNe laser (excitation 488/543 nm; emission 560 nm *long pass*).

*A. thaliana* line *GFP-ATG8a* was used to study the formation of autophagosomes. Each group of samples consisted of at least three independent biological replicates. At least 25 cells were counted per each replicate^91^. The GFP-labeled autophagosome structures were counted using Image J software.

### Gene expression analyses based on transcriptomic data

Transcriptomic data for *A. thaliana* was obtained from a publicly available database (https://bar.utoronto.ca/eplant)^42^. The expression levels of the identified genes in two different tissues at two developmental stages were taken for the analysis. The expression was analyzed in the cotyledon and hypocotyls of 7-day-old plants and in root and vegetative leaf rosette of 14-day-old plants. Expression heatmaps were constructed using Heatmap tool in TBtools v1.0971 software^88^.

### Statistical analysis

Statistical data processing was performed using OriginPro 2019b software. In particular, the reliability of the results was confirmed with the Student’s *t* test. Variation of means were expressed as a standard deviation (STD) or as a standard error of mean (SEM) for gene expression data. To determine whatever the datasets are significantly different, one-way or two-way ANOVA test were performed, which included the calculation of Fisher’s least significant differences (LSDs). The LSDs were used to determine homogeneous dataset groups at different level of significance (*p* < 0.05, *p* < 0.01, *p* < 0.001).

### Reporting summary

Further information on research design is available in the Nature Research Reporting Summary linked to this article.

### Supplementary information

SUPPLEMENTAL MATERIAL

Reporting Summary

## Data Availability

All data generated or analyzed during this study are included in this published article and its supplementary information files. Any detailed data supporting the findings of this study are available from the corresponding authors upon reasonable request.
