# Supplementary material for: Autophagy formation, microtubule disorientation, and alteration of ATG8 and tubulin gene expression under simulated microgravity in Arabidopsis thaliana
Source: NPJ Microgravity. 2024 Mar 18;10:31. doi: 10.1038/s41526-024-00381-9 (PMC10948825; doi:10.1038/s41526-024-00381-9)
Supplement: Supplementary file 1 — SUPPLEMENTAL MATERIAL [file 41526_2024_381_MOESM1_ESM.pdf]

Autophagy formation, microtubule disorientation, and alteration of ATG8 and tubulin gene expression under simulated microgravity in *Arabidopsis thaliana*

Alla Yemets*, Ruslana Shadrina, Rostyslav Blume*, Svitlana Plokhovska, Yaroslav Blume*

Institute of Food Biotechnology and Genomics, National Academy of Sciences of Ukraine, Baidy-Vyshnevetskoho St., 2a, Kyiv, 04123, Ukraine

*Corresponding authors' e-mail: yemets.alla@nas.gov.ua; blume.rostislav@gmail.com; blume.yaroslav@nas.gov.ua

*Alla Yemets, yemets.alla@nas.gov.ua, orcid.org/0000-0001-6887-0705

Ruslana Shadrina, ruslanashadrina@gmail.com, orcid.org/0000-0001-5666-5034

*Rostyslav Blume, blume.rostislav@gmail.com, orcid.org/0000-0003-4936-1803

Svitlana Plokhovska, svetaplohovska@gmail.com, orcid.org/0000-0001-6178-3529

*Yaroslav Blume, blume.yaroslav@nas.gov.ua, orcid.org/0000-0001-7078-7548

Supplementary Tables

Supplementary Table 1. The list of primers used in the study

Target gene	Direction	Primer sequence (5´-3´)	Product length, b.p.
AtATG8a	F	GGGTTTGTTTCTCCCCCGAT	358
	R	AGCAGTTGGAGGCAATGTGT	
AtATG8b	F	ACCCTAAATCCATCCTCTCTGTT	413
	R	TGGCTTTTTCAGCTCCAAGC	
AtATG8c	F	ACAGGACAAATGGCAACAAAGG	425
	R	GCACAAAAGCATTGGAAGGCA	
AtATG8d	F	TCTTGTGTTCCGTGGTCGAG	559
	R	ACACCATGAACGGAAGACCT	
AtATG8e	F	CAGAGTTTTATCCTTATCCGTTCCA	453
	R	AGCATTAAAACGTGGCGGAT	
AtATG8f	F	GTAGTCTACAGGCGTGGAAGG	480
	R	AGGTGGCTTGCTTGCTTAGTA	
AtATG8g	F	GTCAGCTTCAGGCAGGATCA	470
	R	ACCGATTGGTTGTGCCTACAT	
AtATG8h	F	TGCCAGACATGGAGAAGAACA	454
	R	CGTGTGAATCATCATCACTTGGT	
AtATG8i	F	CCTTTTGACTTCGCCGCTTG	429
	R	ATCAACCAAAGGTTTTCTCACTGC	
AtTUA1	F	GGGAGAGCCAACAAAACAACC	560
	R	GGACAAGCGCTCCAACAGTA	
AtTUA2	F	CTGAGGTTCGATGGTGCCTT	654
	R	TCCCACATAAAACCGACAACCT	
AtTUA3	F	TTGTTGACTGGTGCCCAACT	585
	R	TAGCGCACCGATAGACGAGA	
AtTUA4	F	TTTCTTCCACGAAAATGAGAGAGTG	437
	R	AACAGCGTTGAAGACGAGGA	
AtTUA5	F	GCAGAGCATCGCTCGAATTA	470
	R	CCACCACCAACAGCATTGAA	
AtTUA6	F	ACAGCATCAAACACCTTCCTCA	459
	R	TGTAATGACCACGGGCGAAA	
AtTUB1	F	GGTGATTCCGCCGATCTTCA	78
	R	ACGAGGAACGTAACGACCAC	
AtTUB2	F	ACCAAGATGCAACTGCGGAT	171
	R	ACTGGTAGTTGAGGTGTTCCT	
AtTUB3	F	GAGGCTCGAGAGAAAACCGT	239
	R	ACGAGGAACGAATCTACCGC	
AtTUB4	F	GGCTCCCTCGGATTCGTAAG	241
	R	CCGAGCGAAATGCAACACAA	
AtTUB5	F	AGACAAGGAAACGACTCTTGGA	185
	R	AGCTTGAGAAGCAAGGACGTA	
AtTUB6	F	TCTCACCATCCAACGGCATC	149
	R	CGACCGGAGTAGGAGAGGAA	
AtTUB7	F	GTGAGCGAAGATGCGTGAGA	97
	R	CCCGTGCTCGAGATTAACCA	
AtTUB8	F	AAGTGCAGGAGGAGCAATAACA	153
	R	AAATGCACCATCACACATAGCC	
AtTUB9	F	AAACAGAGGGAGTCGCCAAA	141
	R	CCGCACTGACCTCCTTGAAT	
AtEFα	F	ATTTTCGCCTAACAGATGGATG	208
	R	CCTCAAGAAGAGTTGGTCC	

Supplementary Note 1

The alignment of *AtATG8* gene mRNAs with denoted primers location:

Forward primer - ---

Reverse primer - ---

```
                    cov    pid    1 [         .         .         .         .         :         .         .         . 80
1 ATG8a          100.0% 100.0%      --------------------------------------------------------------------------------
2 ATG8b           92.8%  44.5%      TATATCTTTTCAATTATTTTTAATTATGTAGTCTAAAAATAAATAATATTTAGTATCCATTTAGTTTTGGTTCTTTCGGT
3 ATG8c           85.1%  38.8%      --------------------------------------------------------------------------------
4 ATG8d           82.3%  45.0%      --------------------------------------------------------------------------------
5 ATG8e           74.6%  43.4%      --------------------------------------------------------------------------------
6 ATG8f           90.0%  31.7%      --------------------------------------------------------------------------------
7 ATG8g           79.3%  31.9%      --------------------------------------------------------------------------------
8 ATG8h           72.5%  35.7%      --------------------------------------------------------------------------------
9 ATG8i           74.7%  29.5%      --------------------------------------------------------------------------------

                    cov    pid   81           .         1         .         .         .         .         :         . 160
1 ATG8a          100.0% 100.0%      --------------------------------------------------------------------------------
2 ATG8b           92.8%  44.5%      TCTTTCATTTATAGAATTTAGTATCCATTCGAATATTTTCCAATTTTTGGTTTGCTTTGATTTAATTAGTTTTGCTTTGA
3 ATG8c           85.1%  38.8%      ----------ATAAAATAAATCAACCA--------------------------------AATTAATGAGTC---------
4 ATG8d           82.3%  45.0%      --------------------------------------------------------------------------------
5 ATG8e           74.6%  43.4%      -----------------------------------------------------------ATCCAATCA------------
6 ATG8f           90.0%  31.7%      ------------------GAATTTTTGTAGGTTGCTTATTTGCTTTTTCTTTCATATTTTTTCGTTAATTGTTGTTA
7 ATG8g           79.3%  31.9%      -----------------------------------------------------------ATTCACTAATTC---------
8 ATG8h           72.5%  35.7%      -----------------------------------------------------GTTTCTTCTCGAACAATC---------
9 ATG8i           74.7%  29.5%      --------------------------------------------------------------------------------

                    cov    pid  161           .         .         .         2         .         .         .         . 240
1 ATG8a          100.0% 100.0%      --------------------------------------------------------------------------------
2 ATG8b           92.8%  44.5%      TTTGATTCGGTATATTCGAGTTGGTTTATTTGTCCACGCCTAGTAGTCCTGATCATGTAAATG-----------------
3 ATG8c           85.1%  38.8%      -------------------------------------------------------------AAG----------------
4 ATG8d           82.3%  45.0%      --------------------------------------------------------------------------------
5 ATG8e           74.6%  43.4%      -------------------------------------------------------------TAG----------------
6 ATG8f           90.0%  31.7%      TCTTCAACGATAGCGTTGTGCTCTGGAGAAGTGTTTGATCGCAGAAAAGCTTCGTTCTTTGTAGTCTACAGGCGTGGAAG
7 ATG8g           79.3%  31.9%      ------------------------------------------------GATAATCTTTCGGTAA----------------
8 ATG8h           72.5%  35.7%      --------------------------------------------------------------------------------
9 ATG8i           74.7%  29.5%      --------------------------------------------------------------------------------

                    cov    pid  241           :         .         .         .         .         3         .         . 320
1 ATG8a          100.0% 100.0%      --------------------------------------------------------------------------------
2 ATG8b           92.8%  44.5%      -----ACAGAAAAGCCCAAACGAGATCCAAACCTATAAAGGCCCAATAAATAGTTC---ACAAACTCATGACCACTACAA
3 ATG8c           85.1%  38.8%      -----ACAAAAAAGTTCAACGAGAACCGAATGTTATTCTAAACCAAACCATAATCCGGTACAAATTTCCGGTT-------
4 ATG8d           82.3%  45.0%      --------------------------------------------------------------------------------
5 ATG8e           74.6%  43.4%      -----ACGAAAAG-------------------------------------------------------------------
6 ATG8f           90.0%  31.7%      GAATGGCAAAAAGCT-----------------------------------------CGTTCAAGCAAGAGCATGACTTAG
7 ATG8g           79.3%  31.9%      -----ACAAAAAA---------------------------------------------GG---------------------
8 ATG8h           72.5%  35.7%      --------AAAAA-------------------------------------------------------------------
9 ATG8i           74.7%  29.5%      --------------------------------------------------------------------------------

                    cov    pid  321           .         .         :         .         .         .         .         4 400
1 ATG8a          100.0% 100.0%      ------------------------------ATTAGGAAAACCCTAACTAAATTCCATTA---------------------
2 ATG8b           92.8%  44.5%      TCCAACGACGTCGTATCATCTCGCTGACGTAAGATAAGCATCGTAACTAATTAAAATCCAAGTAATAAGTTTGGGTGCCG
3 ATG8c           85.1%  38.8%      -----------------------CTGCAGCAGGACAA--ATGGCAACAAAGGAAAGTAACGATGATAAGTTTG-------
4 ATG8d           82.3%  45.0%      ---------------------------------ATAAACATTCCAAATAACTA---------------------------
5 ATG8e           74.6%  43.4%      --------------------------------------------------------------------------------
6 ATG8f           90.0%  31.7%      AGAAGAGAAGGGCAGAGGCTGCTAGGATTAGAGAGAAGTATCCTGATAGGATTCCGGTGAGACTTTCAGTGTCTCTCTTA
7 ATG8g           79.3%  31.9%      --------------------------------------------------------------------------------
8 ATG8h           72.5%  35.7%      ----------------------------------------------CGAACTAA--------------------------
9 ATG8i           74.7%  29.5%      --------------------------------------------------------------------------------

                    cov    pid  401           .         .         .         .         :         .         .         . 480
1 ATG8a          100.0% 100.0%      ----------------------------------------TTAATTTCTTTTATA---ATCCC-TTTCTCTTGACATTGA
2 ATG8b           92.8%  44.5%      TGCAGGTAACAATCACTAAACCCTAAATCCATCCTCTCTGTTT----CCTTTATATATATTCT-CTCCTTTT--------
3 ATG8c           85.1%  38.8%      -----------ATCGAAAG---------------------TTTCAGACTTCAATA---AGTTT-TCAGTTTCGTGCATAG
4 ATG8d           82.3%  45.0%      ----------------------------------------TCTCCATTCTTTGGA---GTTAC-CCAATTTCGACGTTGA
5 ATG8e           74.6%  43.4%      --------------AAAAA---------------------------------------GGTTC-CTTTTTTTGACTTTGT
6 ATG8f           90.0%  31.7%      TACATTGATCGATCATACA----------------------------TTTCTAAA---GTTATATGTGATTAGCATATTG
7 ATG8g           79.3%  31.9%      --------------AAAAG---------------------------------------GTTACACGAATTGTATATATAC
8 ATG8h           72.5%  35.7%      ----------------------------------------------------------------CCAAACATAAAATTGA
9 ATG8i           74.7%  29.5%      ----------------------------------------------------------ATTTTCTCAATTATTCCTT---

                    cov    pid  481           .         5         .         .         .         .         :         . 560
1 ATG8a          100.0% 100.0%      CTTCTCTCTC-----TCGATTCTTCTTCTCCAGTTT------------CAATCAAACTCAATTTCTTCTCAACGAACCCT
2 ATG8b           92.8%  44.5%      -TTCATTTT-----TTGA-TCCTCTTCTTCTTCTA------------CAATCAAAATTCATTTTTTTT-----CCTCT
3 ATG8c           85.1%  38.8%      ATTAGGAATC-----TCGA-AACCCTAATTCTGTTTTCTACACGAAACCCATCAAAATCGAATTTT--------------
4 ATG8d           82.3%  45.0%      ACACAAACCC-----TTGCTTCTCTCTTCTCTGTTT----CTCTCTCGCAGCGAAAAATCATG-----------------
5 ATG8e           74.6%  43.4%      ATCCG----------TAGA-TCATCTTCTTCTTCTT------------CTTCCAGAGT----------------------
6 ATG8f           90.0%  31.7%      TTTCCCTTTCTATGTTCGGATCAATCTTTTGTGTTT----GACTGAGTTTATGCAGGTTAAGTTGTTGT------GGTTT
7 ATG8g           79.3%  31.9%      ACATATAACT-----GTGA-----------------------------CGATCGCAGTCGAAG-----------------
8 ATG8h           72.5%  35.7%      ACACA---------------TCCTCTTCCTCTGCTCTCTG--------CAAACAAAAA----------------------
9 ATG8i           74.7%  29.5%      ---------------TTGACTTCGCCGCTTGCTTTTGAGAGACGGAGACAAAAACAC-----------------------
                    cov    pid  561           .         .         .         6         .         .         .         . 640
1 ATG8a          100.0% 100.0%      AATTTCCAACAAATCGGATTCAGTTTCTTC-TTCGGGTATGTTTCTTCTC--------------ACCAATAAATTCGAGA
2 ATG8b           92.8%  44.5%      GATCATCATCGAACC----CTAGTTTCGTCTTTTGGGTACGTTTGATTTC--------------GCCTCTCCACTTTGTC
3 ATG8c           85.1%  38.8%      -----------GATCGTTTCTAATTTCTGCCATT----TGATTCTCTTC--------------ATCAGT----------
4 ATG8d           82.3%  45.0%      -------------------GCGATTAGCTCCTTCAAGCATGAACATCCTCTTGTGGAGATGAATATTGAT----------
5 ATG8e           74.6%  43.4%      -----------------------------------------TTTATCCTT--------------ATCCGT----------
6 ATG8f           90.0%  31.7%      ATGGCCCAGCAATATGGTAGTAATGAACTTATTGGGATATAATGAACTTATTGGGATAGC----ATCTGG----------
7 ATG8g           79.3%  31.9%      ----------------------------------------------------------------ATTAAT----------
8 ATG8h           72.5%  35.7%      -------------------AACACCGATTCGGTCAGGAATCATTCGTCGTGAA-----------ATCTGT----------
9 ATG8i           74.7%  29.5%      ----------------------------------------------------------------ACACGA----------
```

```
                     cov    pid  641      :         .         .         .         .         7         .         . 720
1 ATG8a           100.0% 100.0%  TCTTTTAGGGTTT------TCTGGGTTTGTTTCTCCCCCGATCTAGCAGA--CGATCGAGTTTATTAATTATGAT-----
2 ATG8b            92.8%  44.5%  AATTTCGAGTTTTTGTGG-TTTGGGTTTTTTT----CCCGATTTAGCCTAACAGATAAAGTTT--------CGAGT----
3 ATG8c            85.1%  38.8%  -------------------------------------CACATTTAGGGGAAACGA-GAAGCCC--------TAATC----
4 ATG8d            82.3%  45.0%  ---------------------------------------------GTTGAAGTTACTAAGTTT-----------CA----
5 ATG8e            74.6%  43.4%  ---------------------------------------------------TCCATCAAATTC-----------------
6 ATG8f            90.0%  31.7%  ------------------TTTTAAATGGACACCCTACCCGACTAAAGGATGGTTATCAAGTTATTATTGTTTGATTAGCT
7 ATG8g            79.3%  31.9%  --------------------------------TGAATCACACAAAGTCGAATCCA--AAGTTT--------TTGAT----
8 ATG8h            72.5%  35.7%  --------------------------------------------------------------------------------
9 ATG8i            74.7%  29.5%  ----------------------------------------------------CGAAGAAATCT-----------------

                     cov    pid  721      .         .         :         .         .         .         .         8 800
1 ATG8a           100.0% 100.0%  ------------------CTTTGCTTGCTTGAAATTCGCAGAGACTAATCGAATCGCAATGGCTAAG-------------
2 ATG8b            92.8%  44.5%  ------------------TTTGTTTCTCTTAATCATCGTAGATACTAACCGAATCATCATGGAGAAG-------------
3 ATG8c            85.1%  38.8%  ------------------TTTGATTCTTT----------------------AATCGCCATGGCTAAT-------------
4 ATG8d            82.3%  45.0%  ------------------CTTGTCTCTCTTGTGTTCCG-------TGGTCGAGTCTCTGTTGTTCACGGGGCGTAGAATC
5 ATG8e            74.6%  43.4%  ------------------------TCTCT---------------CTAAGCAAA----GATGAATAAAGGA----------
6 ATG8f            90.0%  31.7%  TCATGCTTGTGATATATGTTTGCCACTGTTTCAACTACATGGAGTTGATTGAGTTATAGTGACTATAACAACATT-----
7 ATG8g            79.3%  31.9%  ------------------CTTAACCAAGTCGCATAATCCAGAGAGGAATCAAAAGAAGATGAGTAACGTC----------
8 ATG8h            72.5%  35.7%  ---------------------------------------------------AATGGGGATTGTTGTC-------------
9 ATG8i            74.7%  29.5%  ------------------------------------------------CCGGGCGGTCGAAGAAGATG------------

                     cov    pid  801      .         .         .         .         :         .         .         . 880
1 ATG8a           100.0% 100.0%  --------AGTTCCTTCAAGATCTCTAAC----CCTCTCGAGGCAAGGATGAGTGAATCT-----------------TCT
2 ATG8b            92.8%  44.5%  --------AACTCCTTCAAGCTTTCTAAT----CCTCTGGAGATGAGAATGGCTGAGTCT-----------------ACT
3 ATG8c            85.1%  38.8%  --------AGCTCTTTCAAGTTGGAACAC----CCACTAGAGAGGAGACAAATTGAATCT-----------------TCT
4 ATG8d            82.3%  45.0%  GCTATTTCAGTCCCTTGGAATCCTAAGATATTATCACTGAAAAAGAGACAAGCTGAAGCA-----------------GCA
5 ATG8e            74.6%  43.4%  --------AGCATCTTTAAGATGGACAAC----GATTTCGAAAAGAGAAAAGCTGAAGCT-----------------GGA
6 ATG8f            90.0%  31.7%  --------AGGACTTTGAATTTAGTTGTC---ATTTTTTGGAACGTGACTTTCTGAAACTCGATTCTTACTGAGTACTTA
7 ATG8g            79.3%  31.9%  --------AG---CTTCAGGCAGGATCAT----GATTTCGAGAAGAGGAAAGCTGAGGCT-----------------TTA
8 ATG8h            72.5%  35.7%  --------AAGTCTTTCAAGGATCAATTC----TCCTCTGATGAGAGATTGAAGGAGTCG-----------------AAC
9 ATG8i            74.7%  29.5%  --------AAATCGTTCAAGGAACAATAC----ACGTTGGATGAAAGGCTCGCGGAGTCG-----------------CGG

                     cov    pid  881      .         9         .         .         .         .         :         . 960
1 ATG8a           100.0% 100.0%  CGAATCAGA----------------------------------------------------------------GAGAAG-
2 ATG8b            92.8%  44.5%  CGTATCAGA----------------------------------------------------------------GCGAAA-
3 ATG8c            85.1%  38.8%  CGCATCAGG----------------------------------------------------------------GAGAAG-
4 ATG8d            82.3%  45.0%  CGTATCAGG----------------------------------------------------------------GAGAAG-
5 ATG8e            74.6%  43.4%  AGGATCAGG----------------------------------------------------------------GAGAAA-
6 ATG8f            90.0%  31.7%  CGGCTTAGATAAGTAGATTTGCATGATTCATGTGGCTACGTGCTTTATGATTCACATGGTAAAAATGACACTTGAGATGA
7 ATG8g            79.3%  31.9%  AGGATCAGA----------------------------------------------------------------GAGAAG-
8 ATG8h            72.5%  35.7%  AACATCATT----------------------------------------------------------------GCGAAA-
9 ATG8i            74.7%  29.5%  GAGATAATC----------------------------------------------------------------GCTAAG-

                     cov    pid  961      .         .         .         0         .         .         .         . 1040
1 ATG8a           100.0% 100.0%  -----------------TACCCTGACAGAATTCCCGTGATTGTGGAGAAGGCTGGACAAAGTGATGTTCCTGACATTGAC
2 ATG8b            92.8%  44.5%  -----------------TACCCTGAAAGAGTTCCCGTGATTGTGGAAAAAGCTGGACAGAGTGATGTTCCTGACATTGAC
3 ATG8c            85.1%  38.8%  -----------------TATCCAGACAGAATTCCAGTGATCGTAGAGAGAGCTGAAAGAAGTGATGTTCCCAATATCGAC
4 ATG8d            82.3%  45.0%  -----------------TATCCAGACAGAATACCAGTCATTGTCGAAAGAGCCGAGAAGAGTGATGTCCCGGATATTGAT
5 ATG8e            74.6%  43.4%  -----------------TACCCTGATCGAATTCCTGTGATTGTGGAAAAGGCTGAGAAAAGTGAAGTCCCAAATATAGAC
6 ATG8f            90.0%  31.7%  TGATTTCCACGCATTTATATTTTTGTGGGATCCAGGTGATTGTTGAGAAGGCTGAGAAGAGTGATATACCAACCATCGAC
7 ATG8g            79.3%  31.9%  -----------------TATTCTGACAGAGTCCCGGTGATTGTGGAGAAGTCAGAGAAAAGTGATATCCCAAACATTGAC
8 ATG8h            72.5%  35.7%  -----------------TATCCAGATAGAATACCTGTGATCATTGAGAAATATTCAAACGCAGATCTGCCAGACATGGAG
9 ATG8i            74.7%  29.5%  -----------------TACCCTACTCGGATTCCAGTAATTGCTGAGAAGTATTGCAAAACGGATCTGCCTGCCATCGAG

                     cov    pid  1041     :         .         .         .         .         1         .         . 1120
1 ATG8a           100.0% 100.0%  AAGAAGAAGTATCTTGTACCAGCTGATCTAACAGTGGGACAATTTGTATACGTGGTTCGTAAAAGAATCAAGCTTGGAGC
2 ATG8b            92.8%  44.5%  AAGAAGAAGTATCTTGTACCAGCTGATCTAACCATTGGCCAATTTGTGTACGTTGTGAGGAAAAGAATCAAGCTTGGAGC
3 ATG8c            85.1%  38.8%  AAGAAGAAGTACCTTGTTCCAGCTGATCTAACTGTTGGGCAATTTGTGTATGTTGTCCGTAAAAGAATCAAGCTGAGTGC
4 ATG8d            82.3%  45.0%  AGGAAAAAGTACTTGGTCCCAGCTGATTTGACTGTTGGCCAGTTTGTTTATGTTGTACGGAAGCGGATCAAGCTCAGTCC
5 ATG8e            74.6%  43.4%  AAGAAGAAGTACCTTGTGCCATCAGACCTAACAGTTGGTCAATTTGTGTATGTGATTCGGAAGAGAATCAAACTAAGTGC
6 ATG8f            90.0%  31.7%  AAGAAAAAGTACCTAGTCCCGGCTGATCTGACTGTGGGGCAGTTTGTGTATGTCATTCGCAAAAGAATCAAATTGAGTGC
7 ATG8g            79.3%  31.9%  AAGAAGAAATACCTTGTCCCAGCTGATCTAACGGTAGGCCAGTTTGTGTATGTGATTCGTAAGAGAATCCAACTTAGTGC
8 ATG8h            72.5%  35.7%  AAGAACAAATACTTGGTCCCACGAGACATGACTGTTGGACATTTCATCCATATGCTAAGCAAAAGGATGCAGTTAGATCC
9 ATG8i            74.7%  29.5%  AAAAAGAAGTTTCTGGTTCCAAGAGATATGTCAGTTGGCCAATTCATCTACATATTGAGTGCTAGGTTACATTTGTCTCC

                     cov    pid  1121     .         .         :         .         .         .         .         2 1200
1 ATG8a           100.0% 100.0%  TGAGAAAGCTATTTTTGTCTTTGTTAAGAACACATTGCCTCCAACT----------------------------------
2 ATG8b            92.8%  44.5%  TGAAAAAGCCATCTTTGTCTTTGTCAAGAACACATTACCACCAACT----------------------------------
3 ATG8c            85.1%  38.8%  CGAAAAGGCTATCTTCGTCTTTGTGAAGAACACATTGCCTCCAACTGGTAAACACACAACTTCTTAGCATAAAAGCTGGT
4 ATG8d            82.3%  45.0%  CGAGAAAGCCATCTTCATTTTTGTCAAGAACATTCTACCACCAACT----------------------------------
5 ATG8e            74.6%  43.4%  AGAGAAAGCTATCTTCATTTTCGTAGACAATGTTCTTCCTCCAACA----------------------------------
6 ATG8f            90.0%  31.7%  AGAAAAGGCTATCTTCATATTCGTGGACAATGTTCTTCCTCCAGCA----------------------------------
7 ATG8g            79.3%  31.9%  AGAGAAAGCTATCTTCATCTTTGTTGATAATGTTCTTCCTCCCACC----------------------------------
8 ATG8h            72.5%  35.7%  ATCCAAAGCTCTCTTTGTTTTCGTACACAACACTCTTCCTCAAACC----------------------------------
9 ATG8i            74.7%  29.5%  TGGTAAAGCCTTATTCGTGTTTGTCAACAACACTCTCCCTCAAACT----------------------------------

                     cov    pid  1201     .         .         .         .         :         .         .         . 1280
1 ATG8a           100.0% 100.0%  --------------------------------------------------------------------------------
2 ATG8b            92.8%  44.5%  --------------------------------------------------------------------------------
3 ATG8c            85.1%  38.8%  TTATGTTAATTTCGCTCTCATGAAATCCGCTGCTAACTTAGTACTGTAATCTACGCATACACTTCTCTGCAAATTCGAAA
4 ATG8d            82.3%  45.0%  --------------------------------------------------------------------------------
5 ATG8e            74.6%  43.4%  --------------------------------------------------------------------------------
6 ATG8f            90.0%  31.7%  --------------------------------------------------------------------------------
7 ATG8g            79.3%  31.9%  --------------------------------------------------------------------------------
8 ATG8h            72.5%  35.7%  --------------------------------------------------------------------------------
9 ATG8i            74.7%  29.5%  --------------------------------------------------------------------------------

                     cov    pid  1281     .         3         .         .         .         .         :         . 1360
1 ATG8a           100.0% 100.0%  -----------------------------------GCTGCATTGATGTCTGCGATCTATGAAGAACACAAAGATGAGGAT
2 ATG8b            92.8%  44.5%  -----------------------------------GCGGCATTGATGTCTGCAATCTACGAAGAACACAAAGACGAAGAC
3 ATG8c            85.1%  38.8%  CAATGATAAAAGATTTCTTTATTGATGTTTTTGCAGCTGCAATGATGTCTGCAATCTATGATGAGAACAAAGACGAAGAT
4 ATG8d            82.3%  45.0%  -----------------------------------GCGGCAATAATGTCTGCAATTTATGAAGAGCACAAAGACGAAGAC
5 ATG8e            74.6%  43.4%  -----------------------------------GGAGAGCTAATGTCAAGCGTTTACGAGGATAAGAAAGACGAAGAT
6 ATG8f            90.0%  31.7%  -----------------------------------GGTGCGCTCATGTCTTCTGTGTACGAAGAGAAAAAGGATGATGAT
7 ATG8g            79.3%  31.9%  -----------------------------------GGAGCGATGATGTCAACCATTTACGATGAGAATAAGGAAGAAGAC
8 ATG8h            72.5%  35.7%  -----------------------------------GCTAGTCGCATGGACTCTCTCTACAATACTTTCAAGGAAGAAGAC
9 ATG8i            74.7%  29.5%  -----------------------------------GCTGCTCTGATGGACTCGGTCTACGAATCTTACAAAGATGATGAT
```

```
                 cov    pid 1361         .         .         .         4         .         .         .         . 1440
1 ATG8a       100.0% 100.0%     GGGTTCCTCTACATGACTTACAGTGGAGAGAACACTTTTGGA---------------------------TCTCTTACCGT
2 ATG8b        92.8%  44.5%     GGGTTTCTCTACATGACATACAGTGGAGAGAACACATTTGGT-----------------------GGATCTTTCTACTGC
3 ATG8c        85.1%  38.8%     GGGTTTCTCTACATGACTTACAGTGGAGAGAACACCTTTGGTTTGGTTTAAATGTGTGCCTTCCAATGCTTTTGTGCTGA
4 ATG8d        82.3%  45.0%     GGGTTTCTATACATGAGTTACAGTGGTGAGAACACGTTCG-------------------------GGATCTTC-------
5 ATG8e        74.6%  43.4%     GGCTTCCTTTACATCACTTACAGTGGCGAGAACACATTCGGT-----------------------GCTTCTTCAATCTAA
6 ATG8f        90.0%  31.7%     GGGTTCCTCTATGTCACTTACAGCGGAGAAAACACATTTGGATTT--------------------GGATCTCCATAATAG
7 ATG8g        79.3%  31.9%     GGCTTCTTGTATGTTACCTACAGTGGGGAAAACACTTTTGGATCGTCAATGACTTAATT------TGGTGTTCTTGCTTT
8 ATG8h        72.5%  35.7%     GGGTTCTTGTACATGTGCTACAGCACCGAGAAAACTTTCGGCTAACAAAAGATTT----------GTCGCTTCATATCGA
9 ATG8i        74.7%  29.5%     GGATTCGTTTACATGTGCTATAGCAGTGAGAAAACCTTTGGTTGATCCAATAA------------TGATGTTAGTGTCTG

                 cov    pid 1441         :         .         .         .         .         5         .         . 1520
1 ATG8a       100.0% 100.0%     TGCTTGAATA------AAAATCGATCTT-----------------------------------------------TGGAT
2 ATG8b        92.8%  44.5%     TAATTACCTC------AGCTTCTACGTC-----------------------------------------------T-GAT
3 ATG8c        85.1%  38.8%     TGTGTATATT------ATATGCTCTTTT-----------------------------------------------ATAAC
4 ATG8d        82.3%  45.0%     --------------------------TT-----------------------------------------------CTAAT
5 ATG8e        74.6%  43.4%     TCTCTGACCTCTGAGAATCCGCCACGTT-----------------------------------------------TTAAT
6 ATG8f        90.0%  31.7%     AGACCTCTTGCT----GGATATTTCATT-----------------------------------------------TTACT
7 ATG8g        79.3%  31.9%     TAATTGAATG---TAAATATTCCATTTT-----------------------------------------------CTTTT
8 ATG8h        72.5%  35.7%     AATCGAAGCGCACTCTGAATAATCCATT-----------------------------------------------TGTTT
9 ATG8i        74.7%  29.5%     TGTACATATACTTTAAACATAAGATGTTCACTACATCAAAAGACATCAAACACTTGTTAAGCTTTATATGACTGCTGCTT

                 cov    pid 1521         .         .         :         .         .         .         .       6 1600
1 ATG8a       100.0% 100.0%     GACT---------------TTGATGTACATACATAAATCAGGAAGAT------------AAAGA----------------
2 ATG8b        92.8%  44.5%     CCTC---------------TTGATGATTGTACATTCTCGTCGACCTT------------AATAA----------------
3 ATG8c        85.1%  38.8%     ACTA---------------TGAATATGCAAAAGTTATATGTACTACT------------ATTAA----------------
4 ATG8d        82.3%  45.0%     GCGC---------------TTGGAATATGTGGATGCATCAGATTATC---------------AA----------------
5 ATG8e        74.6%  43.4%     GCTTCTTCCCAACTTTCAATTTGTGTGTATAGTTTGTTCTCAATATTTATCATTTCTCAAATAA----------------
6 ATG8f        90.0%  31.7%     CCTC---------------TCTATTTTCACCAAATATTTCATCTTCTTATTAATTACTAAGCAA-------GCAAGCCAC
7 ATG8g        79.3%  31.9%     CTTGTCCGTGAACTTG---CTTAGATGCGGATATGATTATTATGGCTTATGTAGGCACAACCAATCGGTTTGCAAACAAA
8 ATG8h        72.5%  35.7%     GATT---------------CCTATGTTTGTGAATAACGTCAGCTCTT---------------------------------
9 ATG8i        74.7%  29.5%     GTTTTATCCTTAGACTTTGTTCATCTGTGCACAATCGTTCTACTACAGTCTCAGTTCCAACTAG---------TATTTCC

                 cov    pid 1601         .         .         .         .         :         .         .         . 1680
1 ATG8a       100.0% 100.0%     -TGTACA-------TTGCTTCCT-----TCTTTTCTCTGGCTTTTAAC--------------TTTGCTTTGG---ATGTT
2 ATG8b        92.8%  44.5%     -TGT-CA-------TT------T-----TACTTTCTTGG---TTTAAC--------------C-TT--TTGAGCTCTCTT
3 ATG8c        85.1%  38.8%     -TGTAAA-------TG---------------TTGCTTTGAATTTAAGT--------------TGTTAGTTTGGGTT----
4 ATG8d        82.3%  45.0%     -TGTACAGTCTGTCTATTTCTCT-----GCTTGTCTCTAGTTTTCA----------------TGACATTGGACGAAGCTT
5 ATG8e        74.6%  43.4%     -TTGTGA-------TTACTATCT-----TCTTTTCACTTG--TTAAAA--------------TGTACATAGACTAATCTT
6 ATG8f        90.0%  31.7%     CTATGTA------CTTATTCTCTAAGGACCTTTGGTCGGGCGTTAATA--------------TGTA--------------
7 ATG8g        79.3%  31.9%     GTGTTGGTTTTTTTATCCTCTGT-----TTTTTTCTTTGGTCTATATC--------------TATC--------TTTCTC
8 ATG8h        72.5%  35.7%     -------------GTTGTTACTG--------TTATTGTTGTTGTTAGC--------------TGACATTGTA--------
9 ATG8i        74.7%  29.5%     CCTTAACGCTCTCTTTGTCATCATTGTTTATCTTCCTGAAAATGCAAAAGACCAATGGATTGTTTTACTAGGGGAAGAGT

                 cov    pid 1681         .         7         .         .         .         .         :         . 1760
1 ATG8a       100.0% 100.0%     TGGATATCT-TTCCCT-----TTGGGTTTATTAATC---------------------------------------GTCAG
2 ATG8b        92.8%  44.5%     ACTATCTC--TTGCAT-----TTGAAGATGGTA-TTTGAAACA-------------------------------------
3 ATG8c        85.1%  38.8%     ----------TTACTT-----TTGGACACACAA-CTTGTGTCATCCAACT-------------------------ATTAT
4 ATG8d        82.3%  45.0%     TTGAAAC---TTGCAT-----GCTATGTTATTAACATGATAAGGTCTTCC-------------------------GTTCA
5 ATG8e        74.6%  43.4%     ATAATATTATCTTCCT-----TTGGTATTATAAGTTTGTGT---------------------------------------
6 ATG8f        90.0%  31.7%     ---------------------CAGAAAACATAA------------------------------------------AATAA
7 ATG8g        79.3%  31.9%     TTTCTGTGCCTTGCTCGAGCATCACACACATCA-TATGTTGCACCTTCACAGACCACACGAGCTACAAGAGCCAGGTACA
8 ATG8h        72.5%  35.7%     ---------------------CAGAAAAGCTAA------------------------------------------AAAAA
9 ATG8i        74.7%  29.5%     TTTGTGCTTCCTCTC------CAGCAAGCACCACGAAGTGGCCACCAACCG------------------------GCAAA

                 cov    pid 1761         .         .         .         8         .         .         .         . 1840
1 ATG8a       100.0% 100.0%     AAACT----TCTCCTTTTCTTACTTTCCTCTTT----TGCTTTTAC------------------------------TTAA
2 ATG8b        92.8%  44.5%     AGATTCTAATTTGACTCTCTAGTTTCTTTGTTT----TGTTTCCTC------------------------------T---
3 ATG8c        85.1%  38.8%     TAATTCTCTTTTA----CCATTTTTTGATCATC----TATTAATAT------------------------------TTAG
4 ATG8d        82.3%  45.0%     TGGTGTTAATTTGGCTTTATGCTACACTTAATT-------TCAAAC------------------------------TTAG
5 ATG8e        74.6%  43.4%     GGCTTTCTTTCTCCCTCAAAGATTTCATTCATTAGACTGGTATCAT------------------------------TTGA
6 ATG8f        90.0%  31.7%     TGTATATAATCTTCTTTCAAGGATCTAAAAACC----TACT-----------------------------------TTAG
7 ATG8g        79.3%  31.9%     AAGTGTTAGTTTGGTTTT--GGTTTGAGAAATTAGAGTTTTGTCCCGGCAATTCCGGGTCGAGCCCACGGAGGCTGTTGG
8 ATG8h        72.5%  35.7%     AGAAAGACATTTGACTTTGTAAATTATGTA-------TAATCCAAC------------------------------TATG
9 ATG8i        74.7%  29.5%     TAGTTCTGGCTACTTTTGGTATCTTTATTAGAT----TCTGGTTCAGGCTCTGGGCTACAAACATAAGAAATAAGACTTG

                 cov    pid 1841         :         .         .         .         .         9         .         . 1920
1 ATG8a       100.0% 100.0%     ATAACAT-------------------------------------------------------------------------
2 ATG8b        92.8%  44.5%     ---GCAT-------------------------------------------------------------------------
3 ATG8c        85.1%  38.8%     GCAATATATTTGTGGTAAAAAAGACATC----------------------------------------------------
4 ATG8d        82.3%  45.0%     TCGCTATA------------------------------------------------------------------------
5 ATG8e        74.6%  43.4%     --------------------------------------------------------------------------------
6 ATG8f        90.0%  31.7%     G-------------------------------------------------------------------------------
7 ATG8g        79.3%  31.9%     GCTTTATCTAGACCACAATGAAGAACGATTGTGGGCTAGGCCGACGTGGCGGTAAAAGACAAATCACATGGGGAAGCGCC
8 ATG8h        72.5%  35.7%     GCAA----------------------------------------------------------------------------
9 ATG8i        74.7%  29.5%     ACATTGTAAAGGTAAAACACTAAACAAA----AACTTGGAAGAATATAGAGGAAACAGTGTGGAAGAGTATACACATGTA

                 cov    pid 1921         .         .         :         .         .         .         .       0 2000
1 ATG8a       100.0% 100.0%     -----------------------------TGATTTTGGGGCTA-------------------------------------
2 ATG8b        92.8%  44.5%     -----------------------------CTTCTCTATTGCCA-------------------------------------
3 ATG8c        85.1%  38.8%     -------------TACTAAATAAGTAGTGTATCTTTGGTGTTA-------------------------------------
4 ATG8d        82.3%  45.0%     -----------------------------TATAGTTGAGGTCA-------------------------------------
5 ATG8e        74.6%  43.4%     -----------------------------------TGATGCTA-------------------------------------
6 ATG8f        90.0%  31.7%     -----------------------------------TGAATCTA-------------------------------------
7 ATG8g        79.3%  31.9%     ACGTGTTAAAGTTAGCGGCAATGGCGGCTTGGGGTTGCGGTTAGGGCACGACCTTCACGAACAACACAAAAACAGAGCGG
8 ATG8h        72.5%  35.7%     -----------------------------TAATAATAAGACCA-------------------------------------
9 ATG8i        74.7%  29.5%     TTGTCTGTCTGATATCTGGATAATCTTTCCAAGTATAGAGTTA-------------------------------------

                 cov    pid 2001         .         .         .         .         :         .         .         . 2080
1 ATG8a       100.0% 100.0%     -----------------------------------------------ATGAGTAATGAGTCAC------TTTC-------
2 ATG8b        92.8%  44.5%     -----------------------------------------------TTGAATAA--AATAAC------CTCTATAGTAA
3 ATG8c        85.1%  38.8%     -----------------------------------------------TTTAAAATGAATTAAG------TTCTCTTAC--
4 ATG8d        82.3%  45.0%     -----------------------------------------------CTTGGTGT-------C------CTCCTTGAATA
5 ATG8e        74.6%  43.4%     -----------------------------------------------CT--ACATGAATT--C------TTTCTTGGATA
6 ATG8f        90.0%  31.7%     ------------------------------------------------------TCACATCTC------CCTCTTG----
7 ATG8g        79.3%  31.9%     TGATGGTCCACGCACACTTGTCTGCTGTTTCTCGACCCCACACGTTCTTCACTCTGCATTTTC------CTTCCATAATG
8 ATG8h        72.5%  35.7%     -----------------------------------------------AGTGATGATGATTCACACGAAACTT--------
9 ATG8i        74.7%  29.5%     ---------------------------------ACATATTCGCAATTTCTGATGGTAAATGCC------TAGTGTTTATA
```

		cov	pid	2081] 2089
1	ATG8a	100.0%	100.0%	**---------**
2	ATG8b	92.8%	44.5%	**---------**
3	ATG8c	85.1%	38.8%	**---------**
4	ATG8d	82.3%	45.0%	**G--------**
5	ATG8e	74.6%	43.4%	**---------**
6	ATG8f	90.0%	31.7%	**---------**
7	ATG8g	79.3%	31.9%	**TATTCTCCC**
8	ATG8h	72.5%	35.7%	**---------**
9	ATG8i	74.7%	29.5%	**TATTTGTAT**

Supplementary Note 2

The alignment of *AtTUA* gene mRNAs with denoted primers location:

Forward primer - ---
Reverse primer - ---

```
                        cov    pid     1 [         .         .         .         .         :         .         .         . 80
 1 TUA1              100.0% 100.0%       --------------------------------------------------------------------------------
 2 TUA3               99.3%  62.6%       ----------------------------------GTTATTTAAACCGGATAAAAATCGACACCTTCCAGATCTAGATCCA
 3 TUA5               96.7%  66.2%       --------------------------------------------------------------------------------
 4 TUA2               95.0%  61.6%       --------------------------------------------------------------------------------
 5 TUA4               98.8%  58.4%       TTCAAAAATAAGAGAAACGGTCACAAAACGAGCAAAAATTCAAAATTAGAAAATACGCAGCTCACGAGTATCGTGATATT
 6 TUA6               98.8%  60.7%       --------------------------------------------------------------------------------

                        cov    pid    81         .         1         .         .         .         .         :         . 160
 1 TUA1              100.0% 100.0%       -------------------------TTGTCCTTTCACATCTAAAAGCGGGAGAGCC------------AACAAAACAACC
 2 TUA3               99.3%  62.6%       GATCGAATCTCACGTATTTTG-----TCTATGCCCAGTCCCAAAGGCAGGCTGTCACAACTTGGCGGACCCCAACCAATT
 3 TUA5               96.7%  66.2%       --------------------------------ATCAAATCCAATTC----------------------AATAAATTAATC
 4 TUA2               95.0%  61.6%       --------------------------------------------------------------------ATAGCATCAAAC
 5 TUA4               98.8%  58.4%       AACAACAACCCAATTACATCATTACACGTGTCGTTATATACAGATGGGTACTTTCG----TTTATAAATACACATCAAAC
 6 TUA6               98.8%  60.7%       ---------------------------CCTGCCGTTACATACAGATGGGATAATTTCGCTTATAAAATACAGCATCAAAC

                        cov    pid   161         .         .         .         2         .         .         .         . 240
 1 TUA1              100.0% 100.0%       TTC------------------------TTCCTCGCGAAAA-CAGA----------------GAGAAAGAGGAAGAA----
 2 TUA3               99.3%  62.6%       ATCAAATCTCATTCAATAAATAATCAATTTCCCACAAAAATCATG----------------GAAATTAGG--AAAA----
 3 TUA5               96.7%  66.2%       ATT------------------------TTCCCTTCACAAAATATAAACCTAATTTGCCAACGAAGTTGAACCAAAA----
 4 TUA2               95.0%  61.6%       AGC---------------------------TCTGTAAAAA-CCTA----------------GAAATCGAA--AAAATCAG
 5 TUA4               98.8%  58.4%       GCC------------------------TTAC---TAAAAA-CCTA----------------GAAATAGA---AAAATCAG
 6 TUA6               98.8%  60.7%       ACC------------------------TTCCTCATAA----CCTA----------------GAAATCGAA--AATATCAG

                        cov    pid   241         :         .         .         .         .         3         .         . 320
 1 TUA1              100.0% 100.0%       -----------------------GAAGAAGAA------------------------------------------------
 2 TUA3               99.3%  62.6%       -----------------------AAAGAAGATCTCG--CTCACCGCTCTCATTTCCTCAA---------------TTCTC
 3 TUA5               96.7%  66.2%       -----------------------AAAGAAGATCTCACTCTCACCGCCTTCATTTCCTCGATCTCCGTCTTCCTCCTTCTC
 4 TUA2               95.0%  61.6%       ATCTAGATCTA------------AAAGAAGAG-----------------CGTCTTCATAAACGCCCTTCTTCTTCTTCGT
 5 TUA4               98.8%  58.4%       ATCTAGATTCAGTTTTTTTCATCAAAAAAGAG-----------------CGTCTTCATAAACGCC---------CTTCGT
 6 TUA6               98.8%  60.7%       ATCT-------------------AAAGAAGAG-----------------CGTCTTCATAAACGCC---------CTTCGT

                        cov    pid   321         .         .         :         .         .         .         .         4 400
 1 TUA1              100.0% 100.0%       ---GTCTCTGAGAAAT---------------TTTC---------------GAACATGAGGGAGATCATTAGCATTCATAT
 2 TUA3               99.3%  62.6%       CGTCCGTCGAAGAAGCGAATCGCTCGAA---ATTAGGGTTTCTACTGAGAGAAGATGCGAGAAATCATAAGCATTCACAT
 3 TUA5               96.7%  66.2%       CGTCTGTCGCAGAG---CATCGCTCGA----ATTAGGGTTTCTGCTGAGAGAAGATGAGGGAAATTATTAGCATTCATAT
 4 TUA2               95.0%  61.6%       CTTCTTCCCTGCAAATCTAGTTTCTAGATCTTTTCTTTCTTCCGCGAAACGAAAATGAGAGAGTGCATTTCGATCCACAT
 5 TUA4               98.8%  58.4%       CTTCTTCCCTGGAAATCTAG-----------TTTCTTTCTTCCAC-----GAAAATGAGAGAGTGCATTTCGATCCACAT
 6 TUA6               98.8%  60.7%       CTTCTTCCCTGCAAATCTATTTTCTAGATC-TTTCTTTCTTCCGCGAAACGACAATGAGAGAGTGCATTTCGATCCACAT

                        cov    pid   401         .         .         .         .         :         .         .         . 480
 1 TUA1              100.0% 100.0%       CGGACAAGCTGGTATTCAGGTCGGAAATTCTTGTTGGGAGCTTTATTGTCTCGAACATGGTATCCAACCCGATGGCACCA
 2 TUA3               99.3%  62.6%       CGGACAAGCTGGGATCCAGGTCGGAAATTCGTGTTGGGAGCTTTACTGTCTCGAACATGGAATCCAGCCGGACGGAATGA
 3 TUA5               96.7%  66.2%       CGGACAAGCCGGGATCCAAGTCGGAAATTCCTGCTGGGAGCTTTACTGTCTCGAACATGGAATCCAGCCCGACGGAATGA
 4 TUA2               95.0%  61.6%       TGGTCAGGCCGGTATCCAGGTCGGAAATGCTTGCTGGGAGCTTTACTGTCTTGAACATGGCATTCAGCCTGATGGCCAGA
 5 TUA4               98.8%  58.4%       TGGTCAGGCTGGTATCCAGGTCGGAAATGCTTGCTGGGAGCTGTACTGTCTTGAACATGGCATTCAGCCTGATGGCCAGA
 6 TUA6               98.8%  60.7%       TGGTCAGGCTGGTATTCAAGTCGGAAATGCTTGCTGGGAGCTTTACTGTCTTGAACATGGCATTCAGCCTGATGGCCAGA

                        cov    pid   481         .         5         .         .         .         .         :         . 560
 1 TUA1              100.0% 100.0%       TGCCCAGTGATAGTACTGTTGGTGCATGTCATGATGCATTCAACACATTCTTTAGCGAGACAAGCTCAGGCCAACATGTG
 2 TUA3               99.3%  62.6%       TGCCGAGTGATACTACAGTTGGTGTTGCACACGATGCGTTCAATACTTTCTTTAGCGAGACTGGTGCTGGAAGCATGTC
 3 TUA5               96.7%  66.2%       TGCCGAGTGATACTACAGTTGGTGTTGCACACGATGCGTTCAATACTTTCTTTAGCGAGACTGGAGCTGGAAGCATGTT
 4 TUA2               95.0%  61.6%       TGCCGAGTGACAAGACTGTTGGCGGAGGTGACGATGCTTTCAACACCTTCTTCAGTGAGACCGGTGCAGGGAAGCACGTC
 5 TUA4               98.8%  58.4%       TGCCGAGTGACAAGACTGTTGGTGGAGGTGACGATGCTTTCAACACCTTCTTCAGTGAAACCGGTGCAGGTAAGCACGTC
 6 TUA6               98.8%  60.7%       TGCCTGGTGACAAGACTGTTGGTGGAGGTGATGATGCTTTCAACACCTTCTTCAGTGAAACCGGTGCAGGTAAGCACGTC

                        cov    pid   561         .         .         .         6         .         .         .         . 640
 1 TUA1              100.0% 100.0%       CCTCGAGCTGTGTTTCTTGATCTCGAACCAACCGTCATTGACGAAGTAAGAACCGGCACTTACAGACAGCTTTTCCATCC
 2 TUA3               99.3%  62.6%       CCTAGGGCTGTCTTCGTTGATCTCGAGCCTACCGTTATCGACGAAGTTCGTACTGGTACTTACCGTCAACTTTTCCATCC
 3 TUA5               96.7%  66.2%       CCTAGGGCTGTCTTCGTTGATCTCGAGCCTACCGTTATCGACGAAGTTCGTACTGGTACTTACCGTCAACTTTTCCATCC
 4 TUA2               95.0%  61.6%       CCACGTGCTGTCTTTGTTGATCTTGAGCCAACTGTGATCGATGAGGTCAGGACTGGTACTTACCGTCAGCTTTTCCACCC
 5 TUA4               98.8%  58.4%       CCACGTGCTGTCTTTGTTGATCTTGAGCCAACTGTGATCGACGAGGTCAGGACTGGTACTTACCGTCAGCTTTTCCACCC
 6 TUA6               98.8%  60.7%       CCACGTGCTGTCTTTGTTGATCTTGAGCCAACTGTGATCGACGAGGTCAGGACTGGTACTTACCGTCAGCTTTTCCACCC

                        cov    pid   641         :         .         .         .         .         7         .         . 720
 1 TUA1              100.0% 100.0%       CGAGCAACTTATCTCTGGCAAAGAAGATGCAGCCAATAACTTTGCTAGAGGCCATTACACTGTTGGAAGGGAAATTGTTG
 2 TUA3               99.3%  62.6%       AGAGCAGCTCATTTCTGGGAAAGAAGATGCTGCTAACAACTTCGCTAGAGGACATTACACTGTTGGGAAGGAAATTGTGG
 3 TUA5               96.7%  66.2%       AGAGCAGCTCATTTCTGGGAAAGAAGATGCTGCTAACAACTTCGCTAGAGGACATTACACTGTTGGAAAGGAAATTGTGG
 4 TUA2               95.0%  61.6%       TGAGCAACTCATCAGCGGTAAAGAAGACGCAGCTAACAATTTCGCCCGTGGGCATTACACCATTGGGAAAGAGATTGTTG
 5 TUA4               98.8%  58.4%       TGAACAACTCATCAGCGGTAAAGAAGATGCAGCTAACAATTTCGCCCGTGGTCATTACACCATTGGGAAAGAGATTGTTG
 6 TUA6               98.8%  60.7%       TGAACAACTCATCAGTGGTAAAGAAGACGCTGCTAACAATTTCGCCCGTGGTCATTACACCATTGGGAAAGAGATTGTTG

                        cov    pid   721         .         .         :         .         .         .         .         8 800
 1 TUA1              100.0% 100.0%       ACACCTGCCTTGAAAGGCTGAGGAAATTGGCAGACAATTGCACAGGGTTACAAGGATTTTTGGTATTCAATGCTGTTGGG
 2 TUA3               99.3%  62.6%       ATCTATGTCTTGACCGTGTGAGGAAACTTGCCGACAACTGTACTGGCTTACAAGGGTTTTTGGTGTTCAATGCTGTTGGT
 3 TUA5               96.7%  66.2%       ATCTATGTCTTGACCGTGTGAGGAAGCTTGCCGACAACTGTACTGGCTTACAAGGGTTTTTGGTGTTCAATGCTGTTGGT
 4 TUA2               95.0%  61.6%       ATCTGTGCTTGGACCGTATCAGAAAGCTCGCTGATAACTGTACTGGTCTCCAAGGATTCCTCGTCTTCAACGCTGTTGGT
 5 TUA4               98.8%  58.4%       ACCTGTGCTTAGACCGTATCAGAAAGCTTGCTGATAACTGTACTGGTCTCCAAGGATTCCTCGTCTTCAACGCTGTTGGT
 6 TUA6               98.8%  60.7%       ATCTCTGCTTGGACCGGATCAGAAAGCTCGCTGATAACTGTACTGGTCTTCAAGGATTCCTTGTTTTCAACGCTGTTGGT
GGTGG
                        cov    pid   801         .         .         .         .         :         .         .         . 880
 1 TUA1              100.0% 100.0%       GGTGGAACAGGTTCTGGTTTGGGTTCTTTACTGTTGGAGCGCTTGTCCGTAGATTTTGGAAAAAAATCAAAACTCGGTTT
 2 TUA3               99.3%  62.6%       GGTGGAACTGGTTCTGGATTGGGTTCTCTGTTGCTAGAGCGTTTGTCTGTAGATTATGGAAAGAAGTCTAAGCTTGGTTT
 3 TUA5               96.7%  66.2%       GGTGGAACTGGTTCTGGATTGGGTTCTCTGTTGCTAGAGCGTTTGTCTGTAGATTACGGAAAGAAGTCTAAGCTTGGTTT
 4 TUA2               95.0%  61.6%       GGAGGGACTGGATCTGGTCTTGGATCTCTCCTCCTTGAGAGACTTTCTGTTGACTACGGGAAAAAGTCCAAGTTGGGTTT
 5 TUA4               98.8%  58.4%       GGAGGGACTGGATCTGGTCTTGGATCTCTCCTCCTTGAGAGACTTTCTGTTGACTACGGGAAAAAGTCCAAGTTGGGTTT
 6 TUA6               98.8%  60.7%       GGTGGGACTGGATCTGGTCTTGGATCTCTTCTCCTTGAGAGACTTTCTGTTGACTATGGGAAAAAGTCCAAGTTGGGTTT
```

```
                cov    pid  881          .         9         .         .         .         .         :         . 960
1 TUA1       100.0% 100.0%      CACCATCTACCCTTCTCCTCAGGTTTCTACAGCAGTTGTGGAGCCGTATAACAGTGTTCTCTCGACTCATTCTCTTCTCG
2 TUA3        99.3%  62.6%      TACCATATACCCTTCTCCTCAGGTTTCTACTGCTGTTGTAGAACCTTACAACAGTGTTCTCTCAACGCATTCCCTTCTTG
3 TUA5        96.7%  66.2%      TACCATATACCCTTCTCCTCAGGTTTCTACTGCTGTTGTAGAGCCTTACAACAGTGTGCTTTCAACGCATTCCCTTCTTG
4 TUA2        95.0%  61.6%      CACAGTTTACCCATCTCCACAGGTCTCCACCTCTGTTGTTGAGCCTTACAACAGTGTCCTCTCCACTCACTCACTCTTGG
5 TUA4        98.8%  58.4%      CACAGTTTACCCATCTCCACAGGTCTCTACCTCCGTTGTTGAGCCTTACAACAGTGTCCTCTCCACTCACTCTCTCTTGG
6 TUA6        98.8%  60.7%      CACTGTTTACCCATCTCCACAGGTGTCTACCTCTGTGGTTGAGCCTTACAACAGTGTCCTCTCCACTCACTCTCTCTTGG

                cov    pid  961          .         .         .         0         .         .         .         . 1040
1 TUA1       100.0% 100.0%      AACACACAGATGTTGTTGTTCTTTTGGACAATGAAGCCATATACGATATCTGCAGGAGATCTTTAGACATTGAGAGGCCT
2 TUA3        99.3%  62.6%      AACATACGGATGTAGCTGTCCTCTTGGATAACGAAGCCATCTATGACATTTGCCGCAGATCCCTAGATATCGAGAGGCCA
3 TUA5        96.7%  66.2%      AACATACCGATGTAGCTGTCCTCTTGGATAACGAAGCCATCTATGACATTTGCCGCAGATCCCTAGATATCGAGAGGCCA
4 TUA2        95.0%  61.6%      AACACACTGATGTCTCTATCCTCCTCGACAATGAAGCTATCTATGACATCTGCAGACGTTCCCTAAGCATTGAGAGACCC
5 TUA4        98.8%  58.4%      AACACACTGATGTCTCCATCCTCCTCGACAATGAAGCTATCTATGACATCTGCAGACGCTCCCTAAGCATTGAGAGACCA
6 TUA6        98.8%  60.7%      AACACACTGATGTCTCCATCCTCCTCGACAATGAAGCTATCTATGACATCTGTAGACGCTCCCTTAACATTGAGAGACCT

                cov    pid 1041          :         .         .         .         .         1         .         . 1120
1 TUA1       100.0% 100.0%      ACATACTCCAATTTGAACCGCTTGATCTCCCAAACCATCTCTTCGCTTACAACTTCTCTAAGATTTGACGGTGCCATCAA
2 TUA3        99.3%  62.6%      ACCTACACAAACTTGAACAGGTTGATATCACAGATCATTTCATCCTTGACAACATCTTTGAGGTTTGATGGTGCCATCAA
3 TUA5        96.7%  66.2%      ACCTACACAAACTTGAACAGGTTGATATCACAGATCATTTCATCCTTGACAACATCTTTGAGGTTTGATGGTGCCATCAA
4 TUA2        95.0%  61.6%      ACCTACACCAACCTCAACCGTCTCGTCTCTCAGGTTATCTCATCCTTGACTGCTTCTCTGAGGTTCGATGGTGCCTTGAA
5 TUA4        98.8%  58.4%      ACCTACACCAACCTCAACCGCCTCGTCTCTCAGGTTATTTCTTCCTTGACTGCTTCTCTGAGGTTTGATGGTGCCTTAAA
6 TUA6        98.8%  60.7%      ACCTACACCAACCTCAACCGTCTCGTCTCTCAGGTTATTTCTTCCTTGACTGCTTCTCTGAGGTTCGATGGTGCCTTGAA

                cov    pid 1121          .         .         :         .         .         .         .         2 1200
1 TUA1       100.0% 100.0%      TGTGGACATCACCGAGTTCCAGACCAACCTTGTACCTTACCCTCGGATCCACTTCATGCTCTCCTCTTATGCACCTGTGA
2 TUA3        99.3%  62.6%      CGTGGATATCACTGAGTTCCAGACCAATCTTGTCCCATATCCCCGTATCCATTTCATGCTGTCATCTTATGCACCAGTCA
3 TUA5        96.7%  66.2%      CGTGGATATCACTGAGTTCCAGACCAATCTTGTCCCATATCCCCGTATCCATTTCATGCTGTCATCTTATGCACCAGTCA
4 TUA2        95.0%  61.6%      TGTTGATGTGACTGAGTTCCAAACCAACTTGGTCCCATACCCAAGAATCCACTTCATGCTTTCCTCCTATGCCCCAGTCA
5 TUA4        98.8%  58.4%      TGTTGATGTCACTGAGTTCCAAACCAACTTGGTCCCATACCCAAGAATCCACTTCATGCTTTCCTCCTATGCCCCAGTCA
6 TUA6        98.8%  60.7%      TGTTGATGTTACTGAGTTCCAAACCAACTTGGTTCCATACCCAAGGATCCACTTCATGCTTTCGTCCTATGCACCAGTCA

                cov    pid 1201          .         .         .         .         :         .         .         . 1280
1 TUA1       100.0% 100.0%      TTTCATCTGCAAAGGCCTACCATGAGCAGTTTTCGGTCCCTGAGATCACGACTTCCGTCTTTGAACCCTCCAATATGATG
2 TUA3        99.3%  62.6%      TCTCAGCCGCCAAGGCTTACCACGAGCAGCTATCAGTCCCTGAGATCACCAATGCCGTTTTTGAGCCAGCTAGCATGATG
3 TUA5        96.7%  66.2%      TCTCAGCCGCCAAGGCTTACCACGAGCAGCTATCAGTCCCTGAGATCACCAATGCCGTGTTTGAGCCAGCGAGCATGATG
4 TUA2        95.0%  61.6%      TCTCCGCAGAGAAGGCCTTCCATGAGCAGCTCTCGGTTGCTGAGATCACAAACAGTGCTTTTGAGCCAGCATCCATGATG
5 TUA4        98.8%  58.4%      TCTCCGCAGAGAAAGCCTTCCATGAGCAACTCTCAGTTGCTGAGATCACAAACAGTGCTTTTGAGCCAGCTTCCATGATG
6 TUA6        98.8%  60.7%      TCTCCGCGGAGAAGGCCTTCCATGAGCAACTCTCAGTTGCTGAGATCACGAACAGTGCCTTTGAACCTGCTTCCATGATG

                cov    pid 1281          .         3         .         .         .         .         :         . 1360
1 TUA1       100.0% 100.0%      GCTAAATGTGACCCTAGGCATGGCAAGTACATGGCTTGCTGTTTGATGTATCGTGGTGACGTTGTGCCCAAGGATGTCAA
2 TUA3        99.3%  62.6%      GCAAAGTGTGACCCGAGACACGGAAAGTACATGGCCTGTTGTTTGATGTACCGAGGAGATGTTGTTCCCAAAGATGTTAA
3 TUA5        96.7%  66.2%      GCAAAGTGTGACCCAAGGCACGGAAAGTACATGGCATGTTGTTTGATGTACCGAGGAGATGTTGTTCCCAAAGATGTTAA
4 TUA2        95.0%  61.6%      GCAAAGTGTGACCCTCGTCACGGAAAGTACATGGCTTGCTGTTTGATGTACCGTGGTGATGTCGTCCCCAAGGATGTGAA
5 TUA4        98.8%  58.4%      GCTAAGTGTGACCCACGTCACGGAAAGTACATGGCTTGCTGTTTGATGTACCGTGGTGATGTTGTCCCCAAGGATGTAAA
6 TUA6        98.8%  60.7%      GCAAAGTGTGACCCGCGACATGGAAAGTACATGGCTTGCTGTTTGATGTACCGTGGTGATGTAGTCCCCAAGGATGTGAA

                cov    pid 1361          .         .         .         4         .         .         .         . 1440
1 TUA1       100.0% 100.0%      TACAGCCGTTGCTGCCATCAAAGCCAAACGAACAATTCAGTTTGTTGACTGGTGCCCAACTGGATTCAAGTGCGGATAA
2 TUA3        99.3%  62.6%      TGCTGCTGTTGGCACCATCAAGACAAAGAGGACTGTTCAGTTTGTTGACTGGTGCCCAACTGGGTTCAAATGTGGAATCA
3 TUA5        96.7%  66.2%      TGCTGCCGTTGGCACCATCAAGACAAAGAGGACTGTTCAGTTTGTTGACTGGTGCCCAACTGGGTTCAAATGTGGAATCA
4 TUA2        95.0%  61.6%      CGCAGCTGTTGGCACCATCAAGACCAAGCGTACTATTCAGTTTGTGGACTGGTGTCCTACTGGATTCAAGTGTGGAATCA
5 TUA4        98.8%  58.4%      CGCAGCTGTTGGCACCATCAAGACCAAGCGCACTATTCAGTTTGTTGACTGGTGTCCTACTGGATTCAAGTGTGGTATCA
6 TUA6        98.8%  60.7%      CGCAGCGGTTGGCACCATCAAGACCAAGCGAACTATTCAGTTTGTTGACTGGTGTCCTACTGGATTCAAGTGTGGTATCA

                cov    pid 1441          :         .         .         .         .         5         .         . 1520
1 TUA1       100.0% 100.0%      ACTACCAGCCGCCTTCCGTTGTTCCAGGTGGTGACCTGGCTAAGGTTCAACGAGCGGTTTGCATGATCAGCAATAATACT
2 TUA3        99.3%  62.6%      ACTACCAACCTCCAACAGTTGTTCCAGGTGGTGACCTCGCTAAGGTTCAGAGAGCTGTATGTATGATCAGTAACAACACA
3 TUA5        96.7%  66.2%      ACTACCAACCTCCAACAGTTGTTCCAGGTGGTGACCTCGCTAAGGTTCAGAGAGCTGTATGCATGATCAGTAACAACACA
4 TUA2        95.0%  61.6%      ACTACCAGCCACCAACTGTTGTTCCAGGAGGTGATCTTGCTAAAGTCCAGAGAGCTGTGTGTATGATCTCAAACTCCACC
5 TUA4        98.8%  58.4%      ACTACCAGCCACCAACAGTTGTTCCAGGAGGTGATCTTGCTAAAGTCCAGAGAGCTGTTTGCATGATCTCAAACTCGACC
6 TUA6        98.8%  60.7%      ACTACCAGCCACCAACTGTTGTTCCTGGAGGTGATCTTGCTAAAGTCCAGAGAGCTGTTTGTATGATCTCAAACTCAACC

                cov    pid 1521          .         .         :         .         .         .         .         6 1600
1 TUA1       100.0% 100.0%      GCAGTTGCTGAAGTGTTCTCACGTATAGACCATAAGTTTGATCTCATGTACTCCAAAAGAGCCTTTGTCCATTGGTATGT
2 TUA3        99.3%  62.6%      GCAGTTGCAGAGGTGTTCTCACGGATCGACCACAAGTTTGATCTCATGTATGCGAAGAGGGCATTTGTCCACTGGTACGT
3 TUA5        96.7%  66.2%      GCAGTTGCAGAGGTGTTCTCACGGATCGACCACAAGTTTGATCTCATGTATGCGAAGAGGGCATTCGTGCACTGGTACGT
4 TUA2        95.0%  61.6%      AGTGTTGCTGAGGTGTTCTCCCGTATTGATCACAAGTTTGATCTTATGTACGCAAAACGTGCTTTCGTTCACTGGTATGT
5 TUA4        98.8%  58.4%      AGTGTTGCTGAGGTGTTCTCCCGTATTGATCACAAGTTTGATCTTATGTACGCCAAACGTGCTTTCGTTCACTGGTATGT
6 TUA6        98.8%  60.7%      AGTGTTGCTGAGGTATTCTCCCGTATTGATCACAAGTTTGATCTTATGTACGCCAAACGTGCTTTCGTTCACTGGTATGT

                cov    pid 1601          .         .         .         .         :         .         .         . 1680
1 TUA1       100.0% 100.0%      AGGAGAGGGTATGGAAGAAGGAGAGTTCTCTGAGGCTCGTGAGGATCTTGCTGCCCTCGAGAAAGATTATGAGGAAGTTG
2 TUA3        99.3%  62.6%      TGGTGAAGGAATGGAGGAAGGTGAATTCTCTGAGGCACGTGAAGACTTGGCCGCACTGGAGAAAGATTACGAAGAAGTTG
3 TUA5        96.7%  66.2%      TGGTGAAGGAATGGAGGAAGGTGAATTCTCTGAGGCACGTGAAGACTTGGCCGCACTGGAGAAAGACTACGAAGAAGTTG
4 TUA2        95.0%  61.6%      GGGTGAGGGTATGGAAGAAGGAGAATTTTCAGAGGCTCGTGAGGATCTTGCAGCATTGGAGAAGGATTACGAAGAGGTTG
5 TUA4        98.8%  58.4%      GGGTGAGGGTATGGAAGAAGGAGAATTCTCAGAGGCACGTGAGGATCTTGCAGCATTGGAGAAGGATTATGAAGAGGTCG
6 TUA6        98.8%  60.7%      TGGTGAGGGTATGGAAGAAGGAGAATTCTCAGAGGCTCGTGAGGATCTTGCAGCATTGGAGAAGGATTATGAAGAGGTCG

                cov    pid 1681          .         7         .         .         .         .         :         . 1760
1 TUA1       100.0% 100.0%      GTGGAGAAGGAGCGGAGGACGACGACGAAGAAGGCGATGAGTATT-------------AGAAAGA---------------
2 TUA3        99.3%  62.6%      GTGCTGAAGGTGGAGACGATGAAGAAGATGAAGGTGAAGACTATTGACACCTTTTCTCAAAAAAACTGCAACCAAAAAAA
3 TUA5        96.7%  66.2%      GTGCTGAAGGTGGAGACGATGAAGAAGATGAAGGTGAAGACTATTGACACCTTTTCTCAAGCAATGTGCTACCAAAAAGT
4 TUA2        95.0%  61.6%      GTGCTGAAGGTGGTGACGATGAGGATGATGAAGGAGAGGAGTACT-------------AAGAAGGTTG------------
5 TUA4        98.8%  58.4%      GTGCTGAAGGTGGTGACGATGAAGATGATGAAGGAGAGGAATACT-------------AAGAAGAATGTTTCTAAAAACT
6 TUA6        98.8%  60.7%      GTGCTGAAGGTGGTGACGATGAGGATGATGAAGGAGAGGAATACT-------------AAGAAGAATGTTTCTTAAAAAT

                cov    pid 1761          .         .         .         8         .         .         .         . 1840
1 TUA1       100.0% 100.0%      -------CACAAGAGTGTGTGTTCCCTTGTTTCTTT-----------------------CTTGTGAAGTACAGTAATTAT
2 TUA3        99.3%  62.6%      GTTTTTTCGTTTATCTGTTTTCACTTTTGATTCTGT----------TGATTGTTTAAATCCTGTAAA-----ATATTCGT
3 TUA5        96.7%  66.2%      TT--TTTCGTTTATCTGTTTTCATTAATGATTCTTG-TTGATTACATGTTTGTTTTAATCCTGTACA-----ATATTCGA
4 TUA2        95.0%  61.6%      TCGGTTTTATGTGGGATT----TCTATTATCTCGTGTTTGTGTGAATAG--GCTCGAAACTCTTACGAGTC-GTACTTGT
5 TUA4        98.8%  58.4%      TTGGATTTGTGTGGGTTT---CTCTATAATCTCGTC-TTGTGAGAATGG--GCTCAAAACTCTTGGGAGTCTTTAATCGT
6 TUA6        98.8%  60.7%      TGGATTTTGTGTTGGGTTTTTCTCTATTATCTCGTGTTGTGATGAATGGGGGCTCGAAACTCTTACGAGTC-TTAGTTGT

                cov    pid 1841          :         .         .         .         .         9         .         . 1920
1 TUA1       100.0% 100.0%      ---GTCTTTGTGAATGTGATCTC-------TACAGTTATTTT------GTGATGTTTACG---GGTCTCTCCCCTTTTAT
2 TUA3        99.3%  62.6%      TCGGTCTTCGTG--TTTTATCTCGGTTGGATACATTTTCATTGGTAGAGCGACCATTGCG--AGTTCCCGAGTTCATTAT
3 TUA5        96.7%  66.2%      TCCTCCTTCGTG--TTTCATCTCGGTTAGATACATTTTCATTGGTAGAGCGACCATTGGGGAAGATCCCGAGTTCATTAT
4 TUA2        95.0%  61.6%      ---GTTTTCAAAAACCATATTTC-------TATCTCTTGCTT------GTGGCGGTAGCC-----------ATTTGT--T
5 TUA4        98.8%  58.4%      GTGTGTTTTAAAACCTACTTCTC-------TATCTTTTCGTA------GCCATGTTATCT-----------CTCTATTAT
6 TUA6        98.8%  60.7%      GTGTTTTTCTAAACCTATATTTC-------TATCTCTTGCTG------GTGGT--TTGAT-----------TTCTATTAT
```

```
                         cov    pid 1921          .         .         :         .         .         .         .         0 2000
1 TUA1                100.0% 100.0%     TTCTCATTTGTGTTTACTAAA---GGATTGCATTTCTAGTG---------CATCTCAATACATAGTT-------------
2 TUA3                 99.3%  62.6%     CAAGCTTTTGT-TGTACCAAACTTTGATAATCTTCTTACAACTGTC----CATTTTTCCTTGAAGTA---------TAA-
3 TUA5                 96.7%  66.2%     CAAGCTTTTGT-TGTACGAAACCTTCATTATCTTCTTA-CATTGTC----CATTTT------------------------
4 TUA2                 95.0%  61.6%     CTAGTTCGTTTG--------------------TTTCTGATAATATTTT--CATCTC--------------------CAAG
5 TUA4                 98.8%  58.4%     CTATTTCCTTTGTGTGTTAAATCGTTTCTGCCTTTCTGGTAATATCTTATCACTACTATCAAAACTA---------CAA-
6 TUA6                 98.8%  60.7%     CTAGTTCGTTTGTGTATTGAC------TCATGTTTCTGGTAATATC----TTTCTTACTATGTGATAAAGTAATCTCAAA

                         cov    pid 2001          .         .         .         .         :         .         .         . 2080
1 TUA1                100.0% 100.0%     --------------------------------------------------------------------------------
2 TUA3                 99.3%  62.6%     ------------------TCTCGTCTATC---GGTGCGCTAACGTTTATCAACGTTCTGTATATGCTTTGTTGTCGTATC
3 TUA5                 96.7%  66.2%     --------------------------------------------------------------------------------
4 TUA2                 95.0%  61.6%     AACTGAATTTAAGCTGGGTTTGGTTTAAATCGATTGTCCAAAATCTAAACAAA----TAGAAACGTCAAATTGTTATTTG
5 TUA4                 98.8%  58.4%     -----------------GGGTTATTTGAA---GGCATGGAAAAAATTCATGAGATGTTATATATTTTGCATTTTGACCTA
6 TUA6                 98.8%  60.7%     AACTGAATTTTAGCTGGGTATTTTTGTTA---GTTGAACTAGACTTCTCCTAAAGTCAACCTTTATCATATCTGCAT---

                         cov    pid 2081          .         1         .         .         .         .         :         . 2160
1 TUA1                100.0% 100.0%     -------TGAAGGATTTTTGGC------------------------ATTTC-----------------------------
2 TUA3                 99.3%  62.6%     AGC-TAATAATACATTTATGAG------------------------ATTCAACTAATGTTAACAAACC------------
3 TUA5                 96.7%  66.2%     ----------------------------------------------CTTTAGGTACAA----------------------
4 TUA2                 95.0%  61.6%     AG--TGGTGAAAGAT---TGAA------------------------ATTTTTGGT-------------------------
5 TUA4                 98.8%  58.4%     AGCATGATGATACTT---TGGG------------------------ATTCTTGAGAAATATGTGGCAGTGAGGATATAGC
6 TUA6                 98.8%  60.7%     AAGCTGCTAAGAACTATCTGGATACAAAAGATGGCACTGAAACATTATTTCAGTA-------------------------

                         cov    pid 2161    ] 2164
1 TUA1                100.0% 100.0%     ----
2 TUA3                 99.3%  62.6%     ----
3 TUA5                 96.7%  66.2%     ----
4 TUA2                 95.0%  61.6%     ----
5 TUA4                 98.8%  58.4%     AGAT
6 TUA6                 98.8%  60.7%     ----
```

Supplementary Note 3

The alignment of *AtTUB* gene mRNAs with denoted primers location:

Forward primer - ---
Reverse primer - ---

```
                  cov    pid   1 [       .         .         .         .         :         .         .         . 80
1 TUB1         100.0% 100.0%     --------------------------------------------------------ATTTTTTTAAAA-AGAGAGTA---
2 TUB2          95.8%  63.8%     TTTTTTTTTAAGGAAACTTACACAGTAGAGAAAGAAACGGACCATTTTAAAATTTTGAATTTCGTAAACAAACAAAA---
3 TUB3          97.2%  64.4%     ----------------------------------AAACGGACCATTTTTAAATTTTGAATTTCGAGAA-AAACAAAA---
4 TUB4          93.5%  63.9%     ----------------------------------------------------------------AAGA-AGACGTAA---
5 TUB5          98.7%  66.8%     -----------------------------------TTGTTTTTGTATAGACATATTCATGTGTATACA-ACATGTCA---
6 TUB6          96.8%  61.6%     -----------------------------------------------------CTCCCACATCTTACC-CAACGCCA---
7 TUB7          92.0%  62.2%     ---------------------------------------------CTCCTCTTCTTCTCTCTCTCATA-AAACAAAAGAT
8 TUB8          91.3%  60.6%     ------------------------------------------CACTTCGTCTTCTTCATTTCTTCACA-AGTCATAA---
9 TUB9          92.5%  51.3%     ----------------------------------------------------TATACCCCTCAATCAT-AAACAATA---

                  cov    pid  81         .         1         .         .         .         .         :         . 160
1 TUB1         100.0% 100.0%     --------------------------------TAGTAAAAAAAACAGAGGATAAAAAACGAACAAACG-----GTTAAAG
2 TUB2          95.8%  63.8%     -----------------TAGTAGCCCTTATTAGATAATTGGGAAAAAAAAACAAGAAACAAACACAGAGAGGAGTGAGCA
3 TUB3          97.2%  64.4%     --------------TCGGCGCCTCTTATTAGAGATAATTGGGGGAAAAAAACAAAAAACAAACACAGA-----GAGAGCA
4 TUB4          93.5%  63.9%     --------------------------------------------------ACTATATATAGCTGATTA-----TTACCTA
5 TUB5          98.7%  66.8%     ----------ACAACATACATTGGCAATATATGCACATGCATGCTCCAAGTTGCTAATTAAACACTGA-----ATCTGAA
6 TUB6          96.8%  61.6%     --------------------------------------------------------------------------TCCTCA
7 TUB7          92.0%  62.2%     CCTCTCTGCTTCTCCT----------------------------------------------------------TTCCCT
8 TUB8          91.3%  60.6%     ------------------------------------------------------------------------------CC
9 TUB9          92.5%  51.3%     ----------------------------------------------------------------AACA-----GAGGGAG

                  cov    pid 161         .         .         .         2         .         .         .         . 240
1 TUB1         100.0% 100.0%     CTGGCAAAGATCCCATCTTC------CCCACGCTGTCAAATTTTGTCACTACTTCCCAAAAAAAACTCTCCCGCCTCTCT
2 TUB2          95.8%  63.8%     ATAATAAAAGCTCACTCACT------CAC----------ACTTTTATTCATTTCTCTGAAAAC-----------------
3 TUB3          97.2%  64.4%     ATAATAAAAACTCACTCACT------CTC-----------ACTTTTTTCATTTCTTCAACAAAAAAAACCTAAAGCTCTC
4 TUB4          93.5%  63.9%     TTCAACAAATCCCTCTTTTT------AAA---------------------------------------------------
5 TUB5          98.7%  66.8%     ATCTGAAATTATGCCAATCTAAAAACCTTTCGGCGGTAAATTTTGAGTGAATAAGACAATGTAAAATGACTAAGATAAAA
6 TUB6          96.8%  61.6%     ACGTTGATATCTCACCATCC------AAC-GGCATCCAGCTTCCGATAGATTAAACCAATCACAGCCGTTGGATCAAATT
7 TUB7          92.0%  62.2%     TTTTCAAAATCTCTCTCTCT------CTC---------------------------------------------------
8 TUB8          91.3%  60.6%     GTTTCAAATTCTCTCTCTCT------TTC--------------------------------------------------T
9 TUB9          92.5%  51.3%     TCGCCAAATTTTCTCATCAT------TTT------------CTCGAGAAAATAGACTAAAAGCGA---------------

                  cov    pid 241         :         .         .         .         .         3         .         . 320
1 TUB1         100.0% 100.0%     CTCTCCC---------------------------------TCTCCCTTCTT---ATTCTC--------------------
2 TUB2          95.8%  63.8%     ---CCTT----------------------------AAATCTCCGGATTTCG---ATTCCCGAGGCTCGAAAAAACCCTAA
3 TUB3          97.2%  64.4%     TTTTCTC---------------------------------TCCAGATTTCG---ATTCCCGAGGCTCGAGAGAAAACCGT
4 TUB4          93.5%  63.9%     ---CCTA---------------------------------TTCAAAGCCAA---GGACAAGAAAAAAAAAAAAGAGTCGC
5 TUB5          98.7%  66.8%     AAGTGTTAGGTGGGGTGAAAAAAAACAGAGAATAAAAACCTCGCAAGCGAGGGAAGTGATGAATAACAGAGACAATTGGT
6 TUB6          96.8%  61.6%     TTACCTT---------------------------------TCCCTCTTCTT---ACTCTTTCATAAATAAATCCAACTTC
7 TUB7          92.0%  62.2%     -TCTCTT---------------------------------TTTAGATCCAG---TTTTAGGGT-----------------
8 TUB8          91.3%  60.6%     CTTTCTT---------------------------------TCTCAATTTTTGTAGT------------------------
9 TUB9          92.5%  51.3%     ------T---------------------------------TCCCGAGAGAA---AGAAAGAACAAAAAAAAAACATCCGA

                  cov    pid 321         .         .         :         .         .         .         .       4 400
1 TUB1         100.0% 100.0%     -----------------------------CTCCTTCTTCT--------CCTTCACCATCTCCAGATCCCAAAATCTTCAT
2 TUB2          95.8%  63.8%     A----------------------------TCTCTTCTCCG--------TTCACTCATCCAACC-----------------
3 TUB3          97.2%  64.4%     AAA--------------------------TCTCTTCTCCG--------TTCACTCAATCAACC-----------------
4 TUB4          93.5%  63.9%     CCAT-------------------------TTTCTTCTCAT--------TTTTTTTTTGCTCTTGACGAAGAAACCAAAAA
5 TUB5          98.7%  66.8%     TATATTAGCTGTCACTCCCATCTTTCATATTCCTTCACCA---------TCTCTCTCTCTCTCGATCTTGTGAACCACTA
6 TUB6          96.8%  61.6%     T---TCACTCTCCACCCGTCTCACTTCCTCTCCTACTCCGGTCGACGATTATCCCAATCTCGCCGGGATTCTCCTCTGCA
7 TUB7          92.0%  62.2%     -----------------------------TTTCTTCTCTG--------TGAGC---------------------------
8 TUB8          91.3%  60.6%     -----------------------------TTCATACTCTA--------GCTTACTAATC---------------------
9 TUB9          92.5%  51.3%     G--T----------------------------------------------------------------------------

                  cov    pid 401         .         .         .         .         :         .         .         . 480
1 TUB1         100.0% 100.0%     CGATCATCATCATCATGAGAGAAATCCTCCACGTCCAAGGCGGCCAATGCGGTAACCAAATCGGTTCCAAATTCTGGGAA
2 TUB2          95.8%  63.8%     ----------GAAGATGCGTGAGATTCTTCACATCCAGGGTGGTCAATGCGGTAACCAGATCGGCGCCAAGTTCTGGGAA
3 TUB3          97.2%  64.4%     ----------AAAGATGCGTGAGATTCTTCACATCCAGGGTGGTCAATGCGGTAACCAGATCGGAGCCAAGTTCTGGGAA
4 TUB4          93.5%  63.9%     AAAA------AAAAATGAGAGAGATCCTTCATATCCAAGGCGGTCAATGTGGAAACCAGATCGGAGCAAAGTTCTGGGAA
5 TUB5          98.7%  66.8%     CACACACTAACACAATGAGAGAGATCCTTCACATTCAAGGTGGTCAATGCGGTAACCAGATTGGTTCCAAGTTCTGGGAA
6 TUB6          96.8%  61.6%     CCATAAAATAGAAGATGAGAGAAATCCTTCACATTCAAGGTGGTCAATGTGGGAACCAGATTGGTTCCAAGTTCTGGGAA
7 TUB7          92.0%  62.2%     ----------GAAGATGCGTGAGATTCTTCATATTCAAGGAGGTCAATGCGGAAACCAAATCGGTTCCAAGTTTTGGGAA
8 TUB8          91.3%  60.6%     ----------AAAGATGCGAGAGATTCTTCACATACAAGGTGGCCAATGCGGAAACCAGATCGGAGCTAAGTTCTGGGAA
9 TUB9          92.5%  51.3%     ----------GAAAATGAGAGAAATTCTTCATATTCAAGGAGGTCAGTGCGGAAACCAGATCGGAGCTAAGTTCTGGGAA

                  cov    pid 481         .         5         .         .         .         .         :         . 560
1 TUB1         100.0% 100.0%     GTTATCTGCGACGAACACGGCGTTGATCCCACCGGACGTTACAACGGTGATTCCGCCGATCTTCAGCTCGAACGTATCAA
2 TUB2          95.8%  63.8%     GTGGTTTGCGCCGAGCACGGCATCGATCCAACCGGAAGGTACACCGGAGACTCA---GATCTGCAACTTGAGCGCATCAA
3 TUB3          97.2%  64.4%     GTGGTTTGCGCCGAGCACGGCATCGATCCAACCGGAAGGTACACCGGAGACTCA---GATCTGCAACTTGAGCGCATCAA
4 TUB4          93.5%  63.9%     GTGATCTGCGACGAACACGGCATTGATCACACCGGTCAATACGTCGGCGATTCT---CCGTTACAGCTTGAACGTATCGA
5 TUB5          98.7%  66.8%     GTCATCTGCGACGAGCACGGCATCGATTCCACCGGACGTTACAGTGGAGACACTGCAGATCTCCAGCTTGAACGTATCAA
6 TUB6          96.8%  61.6%     GTTGTATGTGATGAGCATGGTATTGATCCCACTGGTCGTTACGTTGGAAACTCT---GATCTGCAGTTGGAGCGTGTCAA
7 TUB7          92.0%  62.2%     GTGGTTAATCTCGAGCACGGGATTGATCAGACGGGACGGTATGTAGGAGATTCC---GAGTTACAGCTTGAGAGGGTTAA
8 TUB8          91.3%  60.6%     GTAGTTTGCGCCGAGCACGGGATCGATTCGACGGGAAGATACCAAGGAGAGAAC---GATTTGCAACTCGAACGAGTCAA
9 TUB9          92.5%  51.3%     GTTATTTGCGGCGAGCACGGTATTGATCAAACCGGTCAGTCATGTGGCGATACA---GATCTACAGCTTGAAAGGATCAA

                  cov    pid 561         .         .         .         6         .         .         .         . 640
1 TUB1         100.0% 100.0%     TGTTTATTACAATGAAGCTTCTGGTGGTCGTTACGTTCCTCGTGCTGTTCTCATGGATCTCGAGCCTGGTACTATGGATA
2 TUB2          95.8%  63.8%     CGTTTACTACAATGAAGCGAGTTGCGGTAGATTCGTTCCTCGTGCAGTGCTCATGGATTTGGAGCCTGGGACTATGGATA
3 TUB3          97.2%  64.4%     CGTTTACTACAATGAAGCGAGTTGCGGTAGATTCGTTCCTCGTGCAGTGCTCATGGATTTGGAGCCTGGGACTATGGATA
4 TUB4          93.5%  63.9%     TGTCTATTTCAACGAAGCTAGCGGTGGAAAGTACGTTCCTCGCGCTGTTCTTATGGATCTGGAGCCTGGTACCATGGATT
5 TUB5          98.7%  66.8%     TGTCTATTACAATGAAGCTTCAGGTGGAAGATACGTTCCTCGTGCTGTTCTTATGGATCTTGAGCCTGGTACTATGGATA
6 TUB6          96.8%  61.6%     TGTTTACTATAACGAGGCATCCTGCGGAAGATATGTTCCCCGTGCAATTCTCATGGATCTTGAGCCTGGTACTATGGACA
7 TUB7          92.0%  62.2%     TGTATACTACAACGAGGCGAGTTGTGGAAGGTACGTTCCTCGCGCTGTTCTCATGGATTTGGAGCCTGGAACCATGGACA
8 TUB8          91.3%  60.6%     TGTCTACTACAACGAAGCGAGTTGCGGAAGATTCGTCCCACGCGCCGTCCTCATGGATCTAGAGCCTGGTACTATGGATA
9 TUB9          92.5%  51.3%     TGTCTATTTTAACGAAGCTAGCGGAGGCAAATACGTTCCTCGTGCTGTTCTTATGGATCTGGAGCCTGGAACTATGGATT
```

```
                    cov    pid  641          :         .         .         .         .         7         .         . 720
1 TUB1           100.0% 100.0%      GTATCAGATCTGGTCCCTACGGTCAGATTTTCCGTCCTGATAACTTCGTCTTTGGTCAATCCGGTGCCGGAAATAATTGG
2 TUB2            95.8%  63.8%      GTCTCAGATCTGGACCGTACGGTCAGACCTTTCGACCTGATAACTTCGTCTTTGGCCAATCCGGTGCTGGTAACAACTGG
3 TUB3            97.2%  64.4%      GTCTCAGATCTGGACCGTACGGTCAGACCTTTCGACCTGATAACTTCGTCTTTGGTCAATCCGGTGCGGGTAACAACTGG
4 TUB4            93.5%  63.9%      CTCTCAGATCTGGTCCGTTCGGTCAGATTTTCCGTCCTGATAACTTCGTCTTTGGTCAATCTGGTGCCGGAAATAACTGG
5 TUB5            98.7%  66.8%      GTATCAGATCCGGACCGTTTGGTCAGATCTTCCGTCCTGATAACTTTGTCTTTGGTCAGTCTGGTGCTGGTAATAATTGG
6 TUB6            96.8%  61.6%      GTGTCAGAACTGGACCTTATGGTCAAATCTTCAGGCCTGACAACTTTGTTTTCGGGCAATCTGGTGCTGGAAACAACTGG
7 TUB7            92.0%  62.2%      GTGTCAGATCTGGTCCGTACGGTCAGATCTTTCGACCGGATAACTTCGTCTTCGGTCAGTCCGGTGCTGGGAATAACTGG
8 TUB8            91.3%  60.6%      GTGTCAGATCCGGTCCGTACGGTCAGATCTTCCGTCCTGATAACTTCGTATTTGGTCAATCCGGTGCCGGTAACAATTGG
9 TUB9            92.5%  51.3%      CTCTCAGATCTGGGCCGTTTGGTCAGATTTTCCGGCCTGATAACTTCGTTTTTGGTCAGTCTGGTGCTGGGAATAACTGG
```

```
                    cov    pid  721          .         .         :         .         .         .         .         8 800
1 TUB1           100.0% 100.0%      GCGAAAGGTCATTATACTGAAGGTGCTGAACTTATTGATGCTGTTCTCGATGTTGTTCGTAAGGAAGCTGAGAACTGTGA
2 TUB2            95.8%  63.8%      GCCAAGGGACACTACACGGAAGGAGCTGAACTAATCGATTCCGTTCTCGATGTTGTTCGTAAGGAAGCTGAGAACTGTGA
3 TUB3            97.2%  64.4%      GCCAAGGGACACTACACTGAAGGAGCTGAACTAATCGATTCCGTTCTCGATGTTGTTCGTAAGGAAGCTGAGAACTGTGA
4 TUB4            93.5%  63.9%      GCGAAAGGTCATTACACCGAAGGTGCTGAGTTGATTGATTCTGTTCTCGATGTTGTGAGGAAGGAAGCTGAGAACAGCGA
5 TUB5            98.7%  66.8%      GCTAAAGGTCATTACACTGAAGGTGCTGAACTTATTGATGCTGTTCTTGATGTTGTTCGTAAAGAAGCTGAGAACTGCGA
6 TUB6            96.8%  61.6%      GCTAAAGGGCATTACACTGAAGGAGCTGAGCTTATTGATGCTGTACTCGATGTTGTACGCAAAGAGGCTGAGAATTGCGA
7 TUB7            92.0%  62.2%      GCTAAAGGTCACTACACTGAAGGTGCTGAGCTTATTGATTCGGTCCTTGATGTTGTTCGCAAAGAGGCTGAGAACTGTGA
8 TUB8            91.3%  60.6%      GCTAAAGGACACTACACTGAAGGTGCTGAGCTTATCGATTCCGTTCTCGATGTTGTTCGTAAGGAAGCTGAGAATTGTGA
9 TUB9            92.5%  51.3%      GCGAAAGGTCATTACACGGAGGGAGCTGAGTTGATTGATTCTGTGCTTGATGTTGTGAGGAAGGAAGCTGAGAACTGTGA
```

```
                    cov    pid  801          .         .         .         .         :         .         .         . 880
1 TUB1           100.0% 100.0%      TTGCCTTCAAGGGTTTCAAGTATGTCACTCTCTTGGAGGAGGCACGGGTTCAGGAATGGGAACTCTCTTGATATCAAAGA
2 TUB2            95.8%  63.8%      CTGTCTCCAAGGGTTCCAGGTTTGTCACTCGTTGGGAGGAGGAACTGGATCTGGTATGGGAACATTGTTGATCTCTAAGA
3 TUB3            97.2%  64.4%      CTGCCTCCAAGGGTTCCAGGTTTGTCACTCATTGGGAGGAGGAACTGGATCTGGTATGGGAACATTGTTGATCTCTAAGA
4 TUB4            93.5%  63.9%      TTGTCTTCAAGGTTTCCAAGTGTGTCATTCATTGGGAGGAGGAACTGGATCTGGAATGGGAACTCTATTGATTTCTAAGA
5 TUB5            98.7%  66.8%      TTGCCTTCAAGGGTTTCAGGTGTGCCATTCGTTGGGAGGAGGCACAGGTTCAGGAATGGGAACACTATTGATATCGAAGA
6 TUB6            96.8%  61.6%      CTGTCTTCAAGGTTTCCAAGTATGTCACTCACTTGGTGGAGGCACCGGGTCTGGAATGGGAACTCTGCTCATATCTAAGA
7 TUB7            92.0%  62.2%      CTGCCTTCAAGGATTTCAGGTGTGCCATTCCTTGGGAGGAGGAACTGGTTCTGGAATGGGAACTTTGTTGATATCTAAGA
8 TUB8            91.3%  60.6%      CTGCTTGCAAGGATTCCAAGTGTGTCACTCATTAGGAGGAGGAACTGGATCTGGAATGGGAACGTTGTTGATTTCAAAGA
9 TUB9            92.5%  51.3%      TTGTCTTCAAGGTTTTCAAGTTTGTCATTCTTTGGGAGGAGGAACTGGTTCTGGAATGGGAACACTTCTTATTTCTAAGA
```

```
                    cov    pid  881          .         9         .         .         .         .         :         . 960
1 TUB1           100.0% 100.0%      TCCGTGAGGAATACCCAGACAGAATGATGCTCACATTCTCTGTTTTCCCATCTCCAAAGGTCTCAGACACAGTCGTTGAG
2 TUB2            95.8%  63.8%      TCCGTGAAGAGTACCCAGATCGCATGATGCTTACCTTCTCGGTGTTCCCTTCACCAAAGGTTTCTGATACTGTGGTTGAG
3 TUB3            97.2%  64.4%      TCCGTGAAGAGTACCCAGATCGCATGATGCTTACCTTCTCGGTGTTCCCTTCACCAAAGGTTTCTGATACTGTGGTGGAG
4 TUB4            93.5%  63.9%      TAAGAGAAGAGTATCCAGATCGTATGATGATGACTTTCTCAGTGTTTCCTTCTCCTAAGGTCTCTGACACTGTTGTTGAG
5 TUB5            98.7%  66.8%      TCCGAGAGGAGTATCCAGACAGAATGATGCTCACTTTCTCTGTTTTCCCATCTCCTAAAGTCTCTGACACAGTCGTTGAG
6 TUB6            96.8%  61.6%      TCAGGGAAGAGTATCCTGATAGAATGATGCTTACATTTTCTGTCTTCCCATCACCAAAGGTCTCTGACACAGTGGTTGAG
7 TUB7            92.0%  62.2%      TCAGGGAGGAGTACCCAGACCGTATGATGATGACTTTCTCTGTGTTCCCATCGCCTAAGGTGTCTGACACTGTTGTGGAG
8 TUB8            91.3%  60.6%      TTAGGGAAGAGTATCCAGATAGGATGATGCTGACGTTTTCTGTATTCCCATCACCGAAAGTGTCAGACACTGTTGTTGAG
9 TUB9            92.5%  51.3%      TTAGAGAGGAATATCCAGATCGTATGATGATGACCTTTTCGGTCTTCCCATCTCCTAAGGTCTCTGATACAGTTGTTGAG
```

```
                    cov    pid  961          .         .         .         0         .         .         .         . 1040
1 TUB1           100.0% 100.0%      CCGTACAATGCAACTCTCTCAGTCCACCAACTTGTAGAGAATGCTGATGAATGCATGGTCCTCGACAACGAAGCTCTTTA
2 TUB2            95.8%  63.8%      CCTTACAACGCTACTCTGTCTGTCCATCAGCTTGTTGAGAATGCTGATGAGTGCATGGTTCTTGATAATGAGGCCTTGTA
3 TUB3            97.2%  64.4%      CCTTACAACGCTACTTTATCTGTTCATCAGCTTGTTGAGAATGCTGATGAGTGCATGGTTCTTGATAACGAGGCCTTGTA
4 TUB4            93.5%  63.9%      CCATACAATGCAACTCTCTCTGTGCATCAGCTTGTCGAAAACGCTGACGAGTGTATGGTTTTGGACAATGAGGCTCTCTA
5 TUB5            98.7%  66.8%      CCTTACAATGCAACTCTCTCTGTCCATCAGCTTGTAGAGAACGCTGATGAATGCATGGTCCTCGACAATGAAGCTCTCTA
6 TUB6            96.8%  61.6%      CCATACAATGCGACACTTTCAGTTCATCAGCTGGTGGAAAATGCTGATGAGTGTATGGTTTTGGACAATGAAGCCCTTTA
7 TUB7            92.0%  62.2%      CCTTACAACGCAACACTATCTGTGCACCAGCTTGTTGAGAACGCTGATGAGTGTATGGTTCTTGACAACGAAGCTTTGTA
8 TUB8            91.3%  60.6%      CCTTATAATGCAACTCTCTCTGTTCATCAGCTTGTGGAGAATGCAGATGAGTGTATGGTTCTTGACAATGAAGCTCTCTA
9 TUB9            92.5%  51.3%      CCATACAATGCTACTCTTTCCGTTCACCAGCTCGTTGAGAATGCTGATGAATGTATGGTTTTGGATAACGAAGCTCTTTA
```

```
                    cov    pid 1041          :         .         .         .         .         1         .         . 1120
1 TUB1           100.0% 100.0%      CGATATCTGTTTCCGCACTCTCAAACTCAGCACTCCTAGCTTTGGAGACTTGAACCACTTGATCTCCGCAACTATGAGTG
2 TUB2            95.8%  63.8%      CGATATTTGCTTCAGGACTCTCAAACTCACTACCCCCAGCTTTGGTGATTTGAACCACTTGATTTCTGCTACTATGTCTG
3 TUB3            97.2%  64.4%      CGATATTTGCTTCAGGACTCTCAAACTCACTACCCCCAGCTTTGGTGATTTGAACCATTTGATATCTGCCACTATGTCTG
4 TUB4            93.5%  63.9%      CGATATCTGTTTCCGTACCCTCAAGCTCGCTAATCCTACCTTTGGTGATCTTAACCATCTCATCTCTGCTACAATGAGTG
5 TUB5            98.7%  66.8%      CGATATCTGCTTCCGTACCCTCAAACTCAGCACTCCTAGCTTTGGAGACTTGAACCATTTGATCTCCGCAACAATGAGCG
6 TUB6            96.8%  61.6%      CGACATCTGTTTTAGAACACTTAAGCTTACCACTCCTAGCTTTGGTGATCTGAATCATCTTATCTCTGCAACCATGAGTG
7 TUB7            92.0%  62.2%      TGACATTTGCTTCCGTACACTCAAGCTTAGCACTCCAAGTTTTGGTGATCTGAACCACTTGATCTCTGCAACCATGTCTG
8 TUB8            91.3%  60.6%      TGACATTTGCTTCAGAACTCTTAAGCTCACCACTCCAAGCTTTGGAGACTTGAATCACCTCATCTCTGCAACCATGAGTG
9 TUB9            92.5%  51.3%      CGATATCTGTTTCCGTACCTTGAAGCTTGCTAATCCTACATTTGGTGACCTTAACCATCTCATCTCAGCTACAATGAGTG
```

```
                    cov    pid 1121          .         .         :         .         .         .         .         2 1200
1 TUB1           100.0% 100.0%      GTGTGACTTGCTCTTTAAGATTCCCTGGACAACTCAACTCTGACCTTAGGAAACTCGCCGTGAACCTTATCCCATTCCCT
2 TUB2            95.8%  63.8%      GTGTGACTTGCTGTCTGAGGTTCCCTGGTCAACTCAACTCTGACCTCCGAAAGCTTGCTGTGAATCTCATCCCATTCCCC
3 TUB3            97.2%  64.4%      GTGTGACTTGCTGTCTGAGGTTCCCTGGTCAACTCAACTCTGACCTCCGTAAGCTTGCTGTGAATCTCATCCCATTCCCT
4 TUB4            93.5%  63.9%      GTGTCACTTGCTGTCTTCGTTTCCCTGGCCAGCTTAACTCTGACCTTAGGAAACTCGCTGTGAACCTTATCCCATTCCCA
5 TUB5            98.7%  66.8%      GTGTGACTTGCTCCTTAAGGTTCCCCGGACAACTAAACTCTGACCTCAGGAAACTCGCGGTGAACCTAATCCCATTCCCT
6 TUB6            96.8%  61.6%      GAGTTACATGCTGTCTTAGGTTCCCGGGTCAGCTCAACTCTGATCTGAGGAAGCTCGCAGTGAACCTCATTCCTTTCCCT
7 TUB7            92.0%  62.2%      GTGTAACCTGCTGTCTTAGGTTCCCTGGTCAACTGAACTCTGATCTGCGAAAGCTAGCTGTGAATCTGATCCCATTCCCT
8 TUB8            91.3%  60.6%      GTGTCACTTGCTGCCTTCGTTTCCCGGGTCAGCTAAACTCTGACCTGCGCAAGCTTGCAGTGAATTTAATCCCATTCCCT
9 TUB9            92.5%  51.3%      GTGTTACATGCTGTCTTCGGTTCCCTGGTCAACTAAACTCTGACCTTAGAAAACTTGCTGTGAACCTTATTCCTTTCCCA
```

```
                    cov    pid 1201          .         .         .         .         :         .         .         . 1280
1 TUB1           100.0% 100.0%      CGTCTACATTTCTTCATGGTTGGTTTCGCCCCTCTCACTTCCCGTGGCTCTCAACAGTACATCTCTCTCACAGTCCCGGA
2 TUB2            95.8%  63.8%      CGTCTTCACTTCTTCATGGTGGGTTTTGCTCCTCTCACCTCAAGAGGTTCTCAGCAGTACCGTTCCCTCACAGTCCCTGA
3 TUB3            97.2%  64.4%      CGTCTTCACTTCTTCATGGTTGGTTTTGCTCCTCTCACCTCAAGAGGTTCACAGCAGTACCGCTCCCTCACAGTCCCTGA
4 TUB4            93.5%  63.9%      AGGCTTCACTTCTTCATGGTTGGTTTCGCACCATTGACATCGAGAGGATCACAGCAATACAGTGCCTTGAGTGTTCCTGA
5 TUB5            98.7%  66.8%      CGTCTCCATTTCTTCATGGTGGGTTTTGCGCCCCTCACTTCCCGTGGCTCGCAACAATACATCTCACTCACCGTTCCTGA
6 TUB6            96.8%  61.6%      CGTCTCCACTTTTTCATGGTTGGTTTTGCCCCTCTCACCTCCCGTGGGTCTCAGCAGTACCGTGCACTCACTGTCCCTGA
7 TUB7            92.0%  62.2%      CGTCTCCACTTCTTCATGGTGGGATTTGCTCCTCTCACCTCTCGTGGATCTCAGCAGTACCGTAACCTCACTGTCCCAGA
8 TUB8            91.3%  60.6%      CGTCTCCACTTCTTCATGGTTGGGTTTGCACCACTCACATCTCGTGGATCACAGCAATACAGAGCCTTAACCGTCCCTGA
9 TUB9            92.5%  51.3%      CGACTCCACTTCTTCATGGTTGGTTTTGCACCTTTGACATCAAGAGGATCTCAACAATACAGTGCCTTGAGTGTCCCTGA
```

```
                    cov    pid 1281          .         3         .         .         .         .         :         . 1360
1 TUB1           100.0% 100.0%      GCTGACACAGCAAATGTGGGACGCTAAGAACATGATGTGTGCAGCTGATCCACGCCACGGACGTTACCTCACAGCTTCAG
2 TUB2            95.8%  63.8%      GCTCACCCAGCAAATGTGGGACTCCAAGAACATGATGTGTGCTGCAGACCCAAGGCACGGACGCTACCTCACAGCCTCTG
3 TUB3            97.2%  64.4%      GCTCACCCAGCAAATGTGGGACTCCAAGAACATGATGTGTGCTGCAGACCCAAGGCACGGACGCTACCTCACAGCCTCTG
4 TUB4            93.5%  63.9%      ACTGACCCAGCAGATGTGGGATGCAAAGAACATGATGTGTGCTGCTGACCCTCGTCATGGACGTTACTTGACTGCATCCG
5 TUB5            98.7%  66.8%      GCTGACTCAACAAATGTGGGACTCCAAAAACATGATGTGTGCAGCTGATCCACGTCACGGACGTTACCTCACAGCCTCTG
6 TUB6            96.8%  61.6%      GCTGACCCAACAGATGTGGGATTCAAAGAACATGATGTGCGCAGCAGACCCGCGCCACGGGCGGTACCTAACTGCCTCGG
7 TUB7            92.0%  62.2%      GCTCACTCAGCAAATGTGGGATGCTAAGAACATGATGTGTGCTGCTGACCCAAGACACGGTCGCTACCTCACTGCTTCAG
8 TUB8            91.3%  60.6%      GCTCACTCAACAGATGTGGGATGCCAAGAATATGATGTGTGCAGCTGATCCACGCCACGGCAGGTACTTGACAGCCTCAG
9 TUB9            92.5%  51.3%      GCTAACTCAACAGATGTGGGATGCCAAAAACATGATGTGTGCAGCTGATCCTCGTCATGGTCGTTACTTGACAGCTTCTG
```

```
                 cov    pid 1361         .         .         .         4         .         .         .         . 1440
1 TUB1        100.0% 100.0%      CCATGTTCCGAGGAAAAATGAGCACAAAGGAAGTGGACGAACAGATCCTAAATGTCCAGAACAAGAACTCATCCTACTTT
2 TUB2         95.8%  63.8%      CCATGTTCCGTGGCAAGATGAGCACAAAGGAAGTTGACGAGCAGATGCTGAATGTGCAGAACAAGAACTCGTCCTACTTT
3 TUB3         97.2%  64.4%      CCATGTTCCGTGGCAAGATGAGCACAAAGGAAGTTGACGAGCAGATGCTGAATGTTCAGAACAAGAACTCGTCCTACTTT
4 TUB4         93.5%  63.9%      CTGTGTTCCGTGGAAAGCTGAGCACCAAAGAGGTTGACGAGCAGATGATGAACATTCAGAACAAGAACTCATCCTACTTT
5 TUB5         98.7%  66.8%      CTATTTTCAGAGGACAGATGAGCACAAAAGAAGTCGACGAGCAAATCCTAAACATACAGAACAAGAACTCGTCTTACTTC
6 TUB6         96.8%  61.6%      CCATGTTCCGTGGCAAGATGAGCACAAAAGAAGTGGACGAGCAGATGATAAACGTACAGAACAAAAACTCTTCCTACTTT
7 TUB7         92.0%  62.2%      CCATGTTCCGTGGAAAGATGAGCACCAAGGAGGTCGATGAACAGATGCTGAACGTACAGAACAAGAACTCCTCCTACTTT
8 TUB8         91.3%  60.6%      CCATGTTCAGAGGCAAAATGAGCACGAAAGAAGTTGACGAACAGATGATCAACGTTCAGAACAAGAACTCGTCATACTTT
9 TUB9         92.5%  51.3%      CTGTCTTCCGTGGAAAGATGAGTACCAAAGAGGTTGATGAACAGATGATGAACGTCCAGAACAAAAACTCCTCATACTTT

                 cov    pid 1441         :         .         .         .         .         5         .         . 1520
1 TUB1        100.0% 100.0%      GTTGAATGGATCCCAAACAATGTGAAGTCCAGCGTCTGCGACATTCCACCAACTGGAATCAAAATGGCTTCCACGTTTGT
2 TUB2         95.8%  63.8%      GTGGAGTGGATCCCCAACAACGTGAAATCGACAGTCTGTGACATCCCACCTACTGGTCTGAAGATGGCATCAACTTTCAT
3 TUB3         97.2%  64.4%      GTGGAGTGGATCCCCAACAACGTGAAATCAACAGTCTGTGATATCCCACCTACTGGTCTGAAGATGGCTTCCACTTTCAT
4 TUB4         93.5%  63.9%      GTGGAATGGATCCCAAACAACGTCAAGTCCAGTGTCTGTGATATTGCACCAAAGGGTTTGAAAATGGCGTCTACTTTCAT
5 TUB5         98.7%  66.8%      GTTGAGTGGATCCCAAACAACGTGAAATCCAGCGTTTGTGATATCCCACCAAAGGGGCTCAAAATGGCGGCGACGTTTGT
6 TUB6         96.8%  61.6%      GTGGAATGGATTCCAAACAACGTGAAGTCAAGCGTCTGTGACATAGCTCCCCGAGGTCTCTCAATGGCATCGACATTTAT
7 TUB7         92.0%  62.2%      GTGGAATGGATCCCTAACAACGTCAAATCAACCGTCTGTGACATTCCCCCTACTGGTCTGAAGATGGCTTCAACGTTCAT
8 TUB8         91.3%  60.6%      GTGGAATGGATCCCAAACAACGTGAAATCAACCGTGTGTGACATTCCACCAACAGGTCTGAAAATGGCTTCGACTTTCAT
9 TUB9         92.5%  51.3%      GTTGAATGGATTCCAAATAACGTGAAATCAAGCGTCTGCGATATAGCTCCCACTGGTTTGAAAATGGCATCAACTTTCAT

                 cov    pid 1521         .         .         :         .         .         .         .         6 1600
1 TUB1        100.0% 100.0%      CGGAAACTCGACTTCGATCCAGGAAATGTTCAGGAGAGTAAGCGAACAGTTCACGGCTATGTTCAGGCGTAAAGCTTTCT
2 TUB2         95.8%  63.8%      TGGAAACTCAACATCAATCCAAGAGATGTTCAGGCGAGTGAGTGAGCAGTTCACAGCTATGTTCAGGAGAAAGGCTTTCT
3 TUB3         97.2%  64.4%      TGGAAACTCAACATCGATCCAAGAGATGTTCAGGCGAGTGAGTGAGCAGTTCACAGCTATGTTCAGGAGGAAGGCTTTCT
4 TUB4         93.5%  63.9%      TGGTAACTCAACCTCAATCCAGGAGATGTTTAGGCGTGTGAGCGAACAGTTCACAGCTATGTTCAGGAGAAAGGCTTTCC
5 TUB5         98.7%  66.8%      GGGTAACTCGACGTCGATTCAAGAAATGTTCAGGAGAGTGAGTGAGCAGTTCACGGCTATGTTTAGGCGTAAAGCGTTTC
6 TUB6         96.8%  61.6%      TGGGAATTCCACATCGATCCAAGAGATGTTTAGGCGGGTGAGCGAGCAGTTCACTGCTATGTTCAGGAGGAAAGCTTTCT
7 TUB7         92.0%  62.2%      TGGTAACTCAACTTCGATCCAAGAGATGTTCAGGCGTGTCAGTGAGCAATTCACTGCTATGTTCAGGAGAAAGGCTTTCT
8 TUB8         91.3%  60.6%      CGGGAACTCGACATCGATCCAGGAGATGTTTAGGCGTGTGAGCGAACAGTTCACAGCGATGTTCAGAAGAAAAGCTTTCT
9 TUB9         92.5%  51.3%      TGGAAACTCAACTTCAATCCAGGAAATGTTCAGGCGTGTGAGTGAACAGTTCACTGCCATGTTCAGGAGAAAGGCTTTCC

                 cov    pid 1601         .         .         .         .         :         .         .         . 1680
1 TUB1        100.0% 100.0%      TGCATTGGTACACAGGAGAAGGAATGGACGAGATGGAGTTCACTGAAGCAGAGAGCAACATGAATGATCTGGTCTCTGAA
2 TUB2         95.8%  63.8%      TGCATTGGTACACAGGTGAGGGAATGGACGAGATGGAATTCACAGAAGCGGAGAGCAACATGAACGATCTTGTGTCAGAG
3 TUB3         97.2%  64.4%      TGCATTGGTACACAGGTGAGGGAATGGACGAGATGGAGTTCACAGAAGCAGAGAGCAACATGAACGATCTTGTGTCAGAG
4 TUB4         93.5%  63.9%      TTCATTGGTACACAGGAGAAGGCATGGACGAGATGGAGTTCACTGAAGCAGAGAGTAACATGAATGATCTTGTCGCAGAG
5 TUB5         98.7%  66.8%      TGCATTGGTACACAGGAGAAGGAATGGACGAAATGGAGTTTACGGAAGCTGAGAGCAATATGAATGATTTGGTCGCTGAA
6 TUB6         96.8%  61.6%      TGCATTGGTACACAGGTGAAGGAATGGACGAGATGGAGTTTACTGAAGCTGAGAGCAACATGAACGATCTAGTCTCAGAG
7 TUB7         92.0%  62.2%      TGCATTGGTACACAGGTGAAGGTATGGATGAGATGGAGTTCACTGAAGCTGAAAGCAACATGAACGATCTTGTCTCTGAG
8 TUB8         91.3%  60.6%      TGCATTGGTACACAGGTGAAGGAATGGACGAGATGGAGTTCACAGAGGCAGAGAGCAACATGAATGATTTGGTATCAGAG
9 TUB9         92.5%  51.3%      TTCATTGGTACACAGGAGAAGGAATGGACGAGATGGAGTTCACTGAAGCAGAGAGTAACATGAATGATCTTGTTGCAGAG

                 cov    pid 1681         .         7         .         .         .         .         :         . 1760
1 TUB1        100.0% 100.0%      TACCAACAATACCAAGATGCTACCGCTGATGAAGAAGACGAATATGATGAAGAAGAAGA------------ACAAGTTTA
2 TUB2         95.8%  63.8%      TACCAGCAATACCAAGATGCAACTGCGGATGAAGAAGGTGACTACGAGGATGAGGAAGAAGGTGAATATCAACAGGAGGA
3 TUB3         97.2%  64.4%      TACCAGCAATACCAAGATGCAACGGCTGATGAAGAAGGTGACTACGAGGATGAGGAAGAAGGTGAATACCAACAGGAAGA
4 TUB4         93.5%  63.9%      TACCAGCAGTACCAAGATGCTACAGCCGGAGAGGAA---GAGTACGAGGAGGAAGAAGAGGAGTACGAGACTTAAGATGT
5 TUB5         98.7%  66.8%      TACCAACAGTACCAAGATGCTACTGCTGATGAGGAAGGTGAGTATGATGTTGAAGAAGAAGAGGAGGG------AGATTA
6 TUB6         96.8%  61.6%      TACCAGCAATACCAAGACGCAACTGCAGATGACGAAGGCGAGTATGAAGAAGACGAGGATGAAGAAGAGATATTGGATCA
7 TUB7         92.0%  62.2%      TATCAGCAGTATCAAGATGCAACGGCTGATGAAGAAGGAGAATATGAAGAGGAAGAAGCTGAGTACGAGCAAGAAGAAA-
8 TUB8         91.3%  60.6%      TATCAACAGTACCAAGACGCAACGGCTGATGAGGAAGAAGGTATGAGTATGAAGAAGACGAAGTGGAAGTGCAGGAGGA
9 TUB9         92.5%  51.3%      TATCAGCAATATCAAGATGCTACAGTCGGTGAAGAG---GAGTACGAGGAGGACGAAGAAGAAGAAGAAGCCTAAAGAAA

                 cov    pid 1761         .         .         .         8         .         .         .         . 1840
1 TUB1        100.0% 100.0%      CGAATCTTGAT-------CTTACAACACAAACCAAAAGATGAAAAAACACAGAAGTCA--------CATCTCTA-----T
2 TUB2         95.8%  63.8%      AGAGTACTGAGAATAAT-TTAGTTATAACTGCTT-----TAAAAACAAAAACAATTTAGTCGTTTGCTACTTT------T
3 TUB3         97.2%  64.4%      AGAGTACTGAGAGTAAT-TTAGTTATAAAAACCGCTTGAAAAAATCA------ATTTAGTCGTTTGCTACTTTT-----T
4 TUB4         93.5%  63.9%      TG----TCAATGGCTCCCTCGGATTCGTAAGCTG-----TGTAAGCAAGCAGCATTCACTTTCTTCTTTCCCCT-----T
5 TUB5         98.7%  66.8%      TGAGACTTGAT-------CCACCAAATCAAACATTTTACTTAAAAGACAAGGAAACGACTCTTGGAAATCTCT------C
6 TUB6         96.8%  61.6%      TGAG--------------TGAGTGAAAAGAGCTGAT-------ATTACCGATTTTTAAATACCTCTCTTATCT------T
7 TUB7         92.0%  62.2%      ------------------CTTATTGAAGAAACAGATGATAAGAACACCTCAACTTCCAGTTGCTGTTTACTTTTAAAACT
8 TUB8         91.3%  60.6%      GCAATAACAACAGCTTCTCTGTTT----------------------------CTCTTAATTCCTTTTTTTTTTTTC---T
9 TUB9         92.5%  51.3%      AGATGATTGATTATTACTTTTGTAA-----------GGCTGTAAGTTCGTCGCACTCATTTCCTTTTACCTCT------T

                 cov    pid 1841         :         .         .         .         .         9         .         . 1920
1 TUB1        100.0% 100.0%      CTCTTTTATTGAAATCTCTATTCCT-----ACTATTACCACTATATATATATAT--------------------------
2 TUB2         95.8%  63.8%      CTTTTTTTTAAAAGGAACACCTC------AACTACCAGTTGTAGTTTTTTT-----------------------------
3 TUB3         97.2%  64.4%      TTCTTAAAAAAAAAAATGAGAACCTC---AACTACCAGTTGCAGGTTTATTT----------------------------
4 TUB4         93.5%  63.9%      ATCCTGAA---------TTTTTTTCTTCGTAATATCTCTTTTATTGTTTCGTTC--------------------------
5 TUB5         98.7%  66.8%      TATTTAAATTTGATTGAAAAACATTGAAAAAATGGGAAATTTGTTTAAATGTGT--------------------------
6 TUB6         96.8%  61.6%      CTTTTCGTTTGGTCGGTATATGTTTATTGAGTTTCATGTATTGTTTGTGATG----------------------------
7 TUB7         92.0%  62.2%      CTCTTCTATTAAGTTCTTATAGTTTCTGTAACTATCGCTTGCGTTATTAGTAGTTATGATACTCT---------------
8 TUB8         91.3%  60.6%      CTTTTGTGTGAGTTTAGTGTCTTCTTATTCATTTTCTCTTCTGGTTTTGGTTTTCTTGTGTTGACATTTGGCTATGTGTG
9 TUB9         92.5%  51.3%      TTTCTGAATTGGGATTCTCTCTTTTTGTGCTTTGTGTGTTTTGTTATTGAATTTC-TTTCCCAAACCTAAGATTTGTTCC

                 cov    pid 1921         .         .         :         .         .         .         .         0 2000
1 TUB1        100.0% 100.0%      ------------------------------ACATGTATCAAGTTGAAGAATCTGG----------------GAAATGTGT
2 TUB2         95.8%  63.8%      ------------------------------CTCTGCTTGTATTTGACATATCTGG----------------TTGATGTTT
3 TUB3         97.2%  64.4%      ------------------------------CTATGCTTGTATTTGACTTATCTGG----------------ATGATGTTT
4 TUB4         93.5%  63.9%      ------------------------------ATGTGTGTTCGTTTTTGTTATTGAA------------------ACCCTAT
5 TUB5         98.7%  66.8%      -------------------------------TTTTTGTTTGAGTAATTTGTTCAA----------------AGAAAGTTT
6 TUB6         96.8%  61.6%      ------------------------------GTCTGTGTGTAATAGTGAGGTCGGA--------------TCTAAACTTTT
7 TUB7         92.0%  62.2%      ------------------------------GCGTAGGCTAAGAGGATTTATCAAT-----------------CAATATTC
8 TUB8         91.3%  60.6%      ATGGTGCATTTATGTCTAAGGCTTAGGAAAATATATGTTTATAAGTCTTTTCGGGTGAGATTGGATTTGAGATATTGTTC
9 TUB9         92.5%  51.3%      AGAGTGTAATCACATTT-------------GCCTATGCTTCTTTGTTTTGTCTATGAAAATATTTCAATGTCAAAAGCCT

                 cov    pid 2001         .         .         .         .         :         .         .         . 2080
1 TUB1        100.0% 100.0%      TTGTTTGTT -------------------------------------------TGTTACTTTATCTTTGTGGT--------
2 TUB2         95.8%  63.8%      CTGTACTTT--------------------------------------------TGTTCTTAATCTTGTTATTGCCTGTGA
3 TUB3         97.2%  64.4%      ATGTACTTT--------------------------------------------GTTTTTAAATTTGGTTCGGTCTTTAGT
4 TUB4         93.5%  63.9%      ATCGGTTCT-----------------------------------------GGATTTGTTAAACTTTTGCGTG--------
5 TUB5         98.7%  66.8%      GAAGTTTCC-------------------------------------------ATTTATT---------------------
6 TUB6         96.8%  61.6%      ATGCGTGGT-------------------------------------------TTTTATATGAAATTGTCCAT--------
7 TUB7         92.0%  62.2%      AAACAATCT------------------------------------------ATTTTGCTAAATCAAAATCCATCCATAGT
8 TUB8         91.3%  60.6%      GTTAGTTTT-----------------------------------------TTTTTCCCTAAACGTTGGTGATTCCATCTG
9 TUB9         92.5%  51.3%      ATACAATTTCTATGATCCAAAAAACTTGATAACCGGTACAAATAGATCCTACATCTCTGCGGTTTTAAGCTCTCCCTGCA
```

```
                   cov    pid 2081           .         1         .         .         .         .         :         . 2160
1 TUB1          100.0% 100.0%       ------------------------------------------------------TCTCTCTTATTGTATTTTGCAACTTA
2 TUB2           95.8%  63.8%       ------------------------------------------------------TGTTTCTATTAATATTTTCCAGTTGT
3 TUB3           97.2%  64.4%       TTGAATCTTCGTTTAAGC------------------------------------TATTATTGACTGTATTGTTCCTAAT-
4 TUB4           93.5%  63.9%       ------------------------------------------------------TATTGCTTATTGTTTTTGTCGGTGAA
5 TUB5           98.7%  66.8%       ------------------------------------------------------TTGGGTGTCTTGTATTTTCCATTTGA
6 TUB6           96.8%  61.6%       ------------------------------------------------------TGTGGTTTTTTATGCTCTTCCTACTT
7 TUB7           92.0%  62.2%       ATTTT-------------------------------------------------TTTTCTTTGTTTCATTATATAAC---
8 TUB8           91.3%  60.6%       ------------------------------------------------------TTTGCTATACCGTATTGTATTAATTA
9 TUB9           92.5%  51.3%       ATCTTTCTTCTTCTTCTTCTTCTGCTTCTTCCTCTTCTGCGATATCTGCCCAGCTTTTCTTTGATTCACTCTTCGGACCA

                   cov    pid 2161           .         .         .         2         .         .         .         . 2240
1 TUB1          100.0% 100.0%       --------------------------------------------------------------------------------
2 TUB2           95.8%  63.8%       A-------------------------------------------------------------------------------
3 TUB3           97.2%  64.4%       --------------------------------------------------------------------------------
4 TUB4           93.5%  63.9%       AAAAATATTGCTTTTG----------------------------------------------------------------
5 TUB5           98.7%  66.8%       GATAATACGTCCTT------------------------------------------------------------------
6 TUB6           96.8%  61.6%       CAATTAATTGGTTTCACAATTTTACAATGTTG------------------------------------------------
7 TUB7           92.0%  62.2%       --------------------------------------------------------------------------------
8 TUB8           91.3%  60.6%       CAC-----------------------------------------------------------------------------
9 TUB9           92.5%  51.3%       TCTGCTGTATCTTTGACGAATTCATGGAGATATTGAATCATGTTCTGGTCCAGTTTCTTTCGCTTCATGGGTTCTTCTTC

                   cov    pid 2241           :         .         .         .         .         3         .         . 2320
1 TUB1          100.0% 100.0%       ----GTTTCTT--------------------------------------------------CTTTTCT-----TCTCATT
2 TUB2           95.8%  63.8%       ----CTGTCTTCAATCTTTGGT---------------------------------------CTTCTAT--------CCTC
3 TUB3           97.2%  64.4%       ----CTCGTTAAAGACTTTGGGCTTATGATTGACTAACCTTTGGGCTTATGATTGACTAACCTTTGGG-----CTTTGGA
4 TUB4           93.5%  63.9%       ----TTCTCTTAAGTTTT--------------------------GTGTTGCA---------TTTCGCT---------CGG
5 TUB5           98.7%  66.8%       ----GCTTCTCAAGCTACTGTGTA--------------------GTATATAA---------ATTTTAC----------AC
6 TUB6           96.8%  61.6%       ----GTTTTTTTGACTGTCGCGGAACCAAGTTATCAATTTAAGGACGCGTGA---------TTTTCAT-----AATCACC
7 TUB7           92.0%  62.2%       ----CTTTTGTTAATTTTGA------------------------CTATGTGA---------ATTCGGTGAAGACTTCACC
8 TUB8           91.3%  60.6%       ----CATTATGGCGTTTTTGCTGGCAGAAAT-------------TCTTATAA---------TTCCTAT-----TTGGATT
9 TUB9           92.5%  51.3%       TCCTCTCTCTAAGATCTTCATCTGTTCAGAGCCAAAAGGTTGTGGTTTTCCAAATAGTAGCTTTCGAG-----CAGCACC

                   cov    pid 2321           .         .         :         .         .         .         .         4 2400
1 TUB1          100.0% 100.0%       GCCTTTATAAT---------------------------------------------------------------------
2 TUB2           95.8%  63.8%       ATCTTTCTGCTACCTGACTACCTC--------------------------------------------------------
3 TUB3           97.2%  64.4%       TTTCTTCATTTATTTTT---------------------------------------------------------------
4 TUB4           93.5%  63.9%       AGCTTTCATTTC--------------------------------------------------------------------
5 TUB5           98.7%  66.8%       TTCTCTCAATTGTCCCAC--------------------------------------------------------------
6 TUB6           96.8%  61.6%       ATTTTTGGATTCCCAATTTCCC----------------------------------------------------------
7 TUB7           92.0%  62.2%       ATCTTTCAAATAT-------------------------------------------------------------------
8 TUB8           91.3%  60.6%       CCCTCTCATATTTGAGGAGATCTT--------------------------------------------------------
9 TUB9           92.5%  51.3%       TCTCGTCTCTTCTTCAAACAATCTGCATCTGCGTGGTTGTGTATATAGACCTGCAGAGTTCGGTCTCACAGATGCTGGAG

                   cov    pid 2401           .         .         .         .         :         .         .         . 2480
1 TUB1          100.0% 100.0%       ---AAAGAGTCTGTCCACGTATC---------------------------------------------------------
2 TUB2           95.8%  63.8%       ---AGTACCTACTTAAGGACTTTAGGTTTGATCTGTT----------------------TGGAAAATA----GATTAAGG
3 TUB3           97.2%  64.4%       ---AAGAGCTCGGCCCATTTTCG------------------------------------TGGTGACTA----AATTACAC
4 TUB4           93.5%  63.9%       ---AGGAGATAAAGCCATACA--------------------------------------CGAAAAATA----AATTTCAG
5 TUB5           98.7%  66.8%       ---ATCGAGTAAATTAGTATAAAGGAAGCTAAACTTTTGCCT-----------------CTATAAATA----AAGATACT
6 TUB6           96.8%  61.6%       ---ATAGGCCATAACCATAGACCA-----------------------------------TAATATACAATACGTAGTTTG
7 TUB7           92.0%  62.2%       ---AAGAAGCAGAGTAGTAGTGACAATGG------------------------------TTATAAGCAATATAAGAATCT
8 TUB8           91.3%  60.6%       ---ATGAGTTTGTTTTGTGTTTTGTTGCTGCTATGTT----------------------TAGTAAACA----CAAATATT
9 TUB9           92.5%  51.3%       ACGAAGAAACATCTGAGTAGTTCTTGTTTGCGGTTCCAGGAGCTATATTTTCCTTCATCCTATTCACA----AATGAACA

                   cov    pid 2481           .         5         .   ] 2514
1 TUB1          100.0% 100.0%       ----------------------------------
2 TUB2           95.8%  63.8%       TTCGGG----------------------------
3 TUB3           97.2%  64.4%       TCTTCG----------------------------
4 TUB4           93.5%  63.9%       TTTCAAT---------------------------
5 TUB5           98.7%  66.8%       TCA-------------------------------
6 TUB6           96.8%  61.6%       TTCTAGTCTAAAATCAGATTAAAACTAAAAAG--
7 TUB7           92.0%  62.2%       TCTTGGTTAAATTTTGGTGTG-------------
8 TUB8           91.3%  60.6%       TCTTGTGGCAGAN---------------------
9 TUB9           92.5%  51.3%       TCCCGCGTAGAACTTCTCCGAAAGATCAGCTTCT
```
